# Combination of Hydrogen and Halogen Bonds in the Crystal Structures of 5-Halogeno-1*H*-isatin-3-oximes: Involvement of the Oxime Functionality in Halogen Bonding

**DOI:** 10.3390/molecules29051174

**Published:** 2024-03-06

**Authors:** Eric Meier, Wilhelm Seichter, Monika Mazik

**Affiliations:** Institut Für Organische Chemie, Technische Universität Bergakademie Freiberg, Leipziger Straße 29, 09596 Freiberg, Germany; eric.meier@chemie.tu-freiberg.de (E.M.); wilhelm.seichter@web.de (W.S.)

**Keywords:** molecular recognition, halogen bonds, supramolecular motifs, oxime group

## Abstract

Various functional groups have been considered as acceptors for halogen bonds, but the oxime functionality has received very little attention in this context. In this study, we focus on the analysis of the hydrogen and halogen bond preferences observed in the crystal structures of 5-halogeno-1*H*-isatin-3-oximes. These molecules can be involved in various non-covalent interactions, and the competition between these interactions has a decisive influence on their self-organization. In particular, we were interested to see whether the crystal structures of 5-halogeno-1*H*-isatin-3-oximes, especially bromine- and iodine-substituted ones, are characterized by the presence of halogen bonds formed with the oxime functionality. The oxime group proved its ability to compete with the other strong donor and acceptor sites by participating in the formation of cyclic hydrogen-bonded heterosynthons oxime∙∙∙amide and O_oxime_∙∙∙Br/I halogen bonds.

## 1. Introduction

Compared to hydrogen bonds, halogen-bonding interactions became the subject of intensive research relatively late, but the studies on this topic quickly led to an enormous increase in knowledge. The progress in this field is characterized by a steadily growing number of publications, including many review articles (for some examples, see [1,2,3,4,5,6,7,8,9,10,11,12,13,14,15]).

A considerable part of the scientific literature addresses how hydrogen and halogen bonds compete with each other [16,17,18,19], and tolerate [19,20,21] or even reinforce each other [22,23]. The knowledge gained from these and other studies can be used for the construction of targeted structures based on the combination of hydrogen and halogen bonds.

Various functional groups have been considered as acceptors for halogen bonds, but the oxime functionality has received very little attention in this context [24,25,26,27]. In contrast, the involvement of this group in the formation of hydrogen bonds (as hydrogen bond donor and acceptor) has been the subject of investigations; however, it should be noted that oximes have been studied in supramolecular chemistry [28,29] and crystal engineering to a far lesser extent than other compounds such as carboxylic acids and amides.

**Figure 1:**
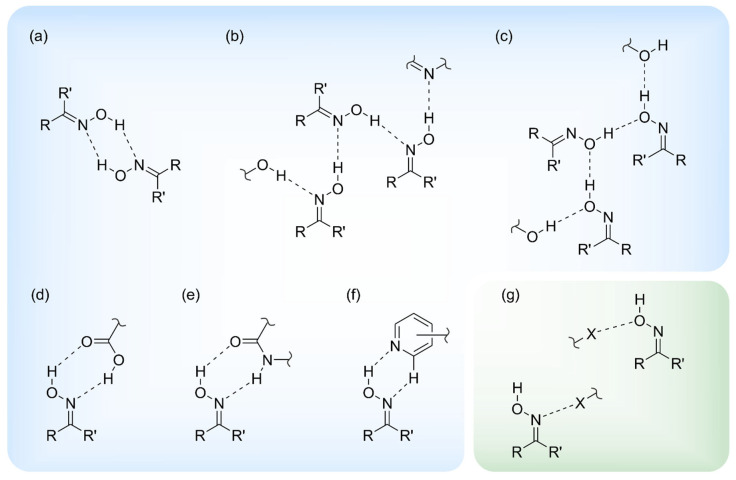
Schematic representation of hydrogen-bonding motifs (**a**–**f**) and halogen bonds (**g**) observed in the crystal structures of compounds with an oxime functionality [hydrogen-bonded heterosynthons are given in (**d**–**f**)].

In the absence of other hydrogen bond donors and acceptors, the oxime···oxime OH···N hydrogen-bonded dimer [Figure 1a; R_2_^2^(6) ring motif] or catemeric motifs directed by O-H∙∙∙N [Figure 1b; C(3) chain motif] or O-H∙∙∙O hydrogen bonds [Figure 1c; C(2) chain motif] can be observed in the crystal structures of oximes [25,30]. The designation of the ring and chain motifs is based on graph theory for categorizing hydrogen bond motifs in molecular crystals [31,32].

The formation of hydrogen-bonded ring motifs has also been observed in the case of the heterosynthons formed by the interactions of the oxime functionality with the carboxyl or amide group [R_2_^2^(7) ring motif], as shown in Figure 1d,e, respectively [33,34,35,36]. It should be noted that theoretical and experimental investigations [33,34,35] revealed a preference for the oxime···carboxyl interaction (heterosynthon shown in Figure 1d) compared to the homologous interactions, such as carboxyl∙∙∙carboxyl and oxime∙∙∙oxime.

In addition to the carboxyl and amide groups, nitrogen heterocycles such as pyridine and quinoline have been considered in the crystallographic investigations. Hydrogen-bonding patterns based on oxime···pyridine/quinoline interactions (see Figure 1f) lead to interesting supramolecular assemblies. For example, α,β-unsaturated ketoximes bearing a terminal pyridine subunit have proven to be valuable building blocks for various supramolecular structures. Depending on the substitution pattern of the molecules, discrete cyclic tetrameric aggregates [37,38] or linear motifs [38,39], including helically grown structures [39], were formed. Furthermore, the potential of the O-H_oxime_∙∙∙N_pyr_ hydrogen bonds for the crystal packing determination was also observed for other compounds containing both oxime and pyridinyl moieties, such as pyridine-aldoxime [40].

In the presence of halogen atoms, the formation of both OH_oxime_∙∙∙X hydrogen bonds and X∙∙∙O_oxime_/N_oxime_ halogen bonds is possible. In the case of hydrogen bonding, a search in the Cambridge Structural Database (CSD, version 5.44, September 2023) [41,42] indicates that, usually, the halogen atoms only act as a second acceptor in bifurcated arrangements involving the oxime functionality (see Figure S1 in Supporting Information). As already mentioned, only a few examples of halogen bonds formed with oxime-based acceptors (Figure 1g) are described in the literature, and, in these examples, the O_oxime_ atom seems to be preferred as the acceptor for halogen bonds. This is in contrast to the situation with hydrogen bonds where the N_oxime_ atom appears to be the favored acceptor site.

**Figure 2:**
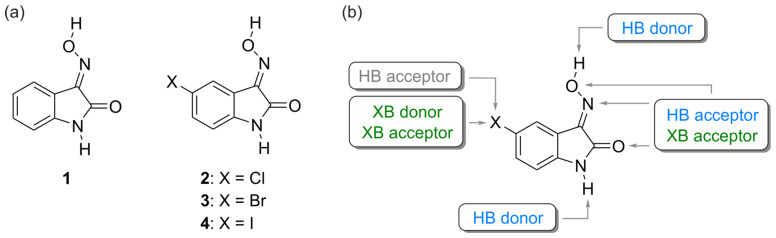
(**a**) Molecular structures of compounds **1**–**4** and (**b**) structure of 5-halogeno-1*H*-isatin-3-oxime with marked hydrogen- and halogen-bonding sites (HB = hydrogen bond, XB = halogen bond).

In this study, we focus on the analysis of the hydrogen and halogen bond preferences observed in the crystal structures of 5-halogeno-1*H*-isatin-3-oximes (see Figure 2a). These molecules can be involved in various non-covalent interactions (see Figure 2b), and the competition between these interactions has a decisive influence on their self-organization. In particular, we were interested in seeing whether the crystal structures of 5-halogeno-1*H*-isatin-3-oximes, especially bromine- and iodine-substituted ones, are characterized by the presence of halogen bonds formed with the oxime functionality. For comparative purposes, the crystal structure of 1*H*-isatin-3-oxime, in which the halogen substituent is lacking, is also considered in this work. 

## 2. Results and Discussion

The syntheses of 1*H*-isatin-3-oxime (**1**) and of 5-halogeno-1*H*-isatin-3-oximes (**2**–**4**) were carried out by reacting isatin (2,3-indolindion) or the corresponding 5-halogeno-1*H*-isatins with hydroxylamine according to the procedure described in the Experimental Section. As indicated by the results of ^1^H NMR spectroscopy, only the (*E*)-isomers are formed in all cases.

**Figure 3:**
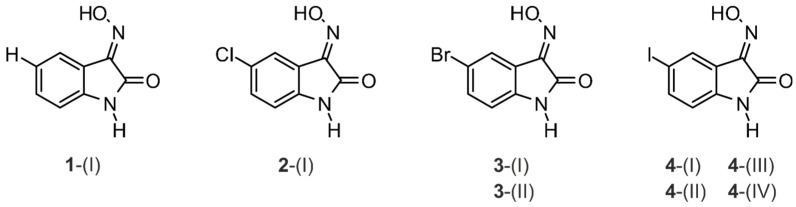
Overview of the eight crystal structures obtained for compounds **1**–**4**.

In the course of the crystallization experiments, single crystals suitable for X-ray diffraction analysis were obtained for all compounds. The crystal samples of compounds **3** and **4** were found to occur in two and four polymorphic forms, respectively, whereas compound **1** and its chlorine-substituted derivative **2** did not occur as polymorphs (for an introduction to polymorphs, see [43,44,45]). The X-ray analyses provided a total of eight crystal structures, an overview of which is shown in Figure 3. 

The characterization of the crystal structures includes the elucidation of the packing and co-ordination behavior of the molecules in the solid state, whereby the analysis of the influence of the halogen atoms on the pattern of non-covalent bonds between the molecules is of particular interest. The crystallographic data and relevant refinement parameters are summarized in Table S1, while information on the intermolecular interactions (hydrogen and halogen bonds, π∙∙∙π interactions) underlying the crystal structures is given in Table S2. To simplify the characterization of the crystal structures, the aromatic ring and the lactam unit of the isatin scaffold are denoted in the following by the abbreviations ‘*Bzn*’ and ‘*Lt*’.

Due to their rigid structure, the isatin oximes do not exhibit any conformational freedom, resulting in an approximately planar molecular geometry in all cases. The structures of the (*E*)-isomers are characterized by an intramolecular hydrogen bond between the oxime O atom and the H atom in the 4-position of the isatin framework (numbering according to the IUPAC nomenclature; the labelling used in the crystal structure analyses does not correspond to this nomenclature, but to the scheme shown in the respective figures).

**Figure 4:**
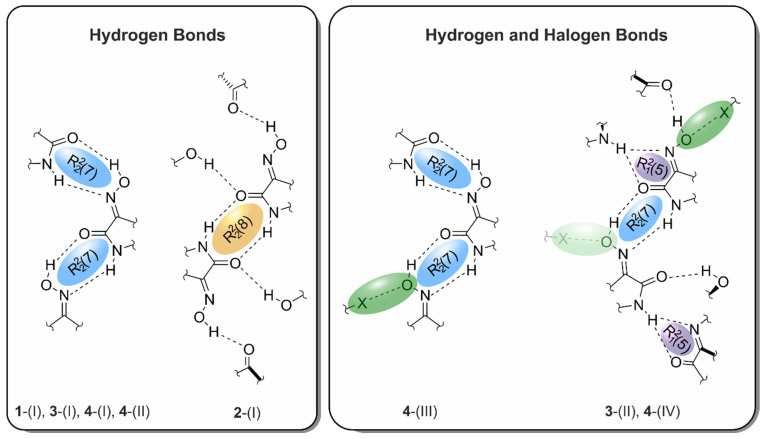
Schematic representation of hydrogen- and halogen-bonding patterns in the crystal structures **1**-(I), **2**-(I), **3**-(I), **3**-(II), and **4**-(I)–**4**-(IV). Faded colors signify interactions that are not included in all of the given crystal structures.

The detailed evaluation of the intermolecular interactions, which revealed the presence of interesting supramolecular motifs within the crystals (for examples, see Figure 4), was supported by the Hirshfeld analysis. The latter represents a suitable tool for visualizing and quantifying the intermolecular contacts present in a crystal structure [46]. This is carried out by a graphical representation of the three-dimensional surface plots of the molecules, as well as their associated two-dimensional fingerprint plots, as given in Section 2.5.

It should be reiterated that the analysis of the literature examples of halogen bonds with oxime-based acceptors suggested the preferred participation of the O_oxime_ atom in such interactions. Furthermore, in view of the results demonstrating the capacity of the oxime functionality to participate in hydrogen-bonded heterosynthons, the formation of the ring motif R_2_^2^(7) based on OH_oxime_∙∙∙O=C/NH∙∙∙N_oxime_ hydrogen bonds (see Figure 1e) and, thus, the absence of oxime∙∙∙oxime homosynthons could be assumed. As shown in Figure 4 and described in the following, crystallographic studies have confirmed this assumption.

### 2.1. Crystal Structure of 1H-isatin-3-oxime [Structure **1**-(I)]

**Figure 5:**
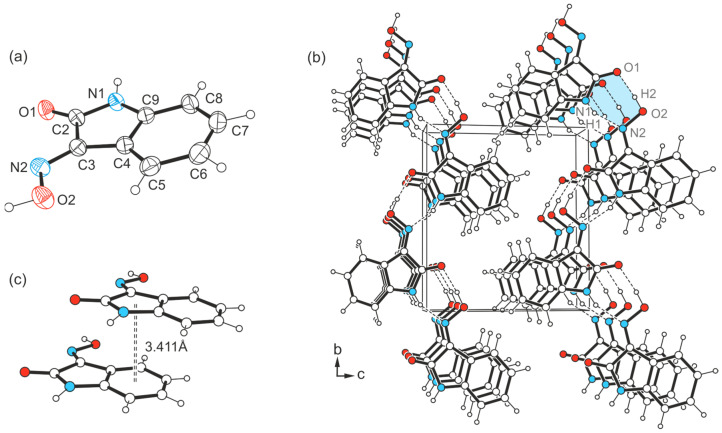
(**a**) Perspective view of the molecular structure of **1**. The displacement ellipsoids of the atoms are shown at a 50% probability level. (**b**) Packing diagram of **1**-(I) viewed in the direction of the *a*-axis. Dashed lines represent hydrogen bonds which form cyclic R_2_^2^(7) synthons highlighted in blue. (**c**) Stacking mode of the molecules in the crystal of **1**.

The crystallization experiments with 1*H*-isatin-3-oxime (**1**) were carried out under the same conditions as those with 5-halogeno-1*H*-isatin-3-oximes **2**–**4** and yielded crystals of the monoclinic space group *P*2_1_ (*Z* = 2) (Figure 5a). The crystal structure of **1** has already been published [47], but without detailed characterization, which we include here for comparison purposes. Apart from a notable improvement of the quality of the underlying data set, the major difference between the two structure models lies in the orientation of the oxime H atom. In the structure reported here, H_oxime_ is positioned coplanar to the isatin scaffold (consistent with all the structures presented below), while, in the structure reported previously [47], it is oriented almost perpendicular to the ring plane. Accordingly, our model exhibits a varied mode of non-covalent interconnection between the molecules.

The packing diagram depicted in Figure 5b indicates that the molecules are linked to infinite zigzag-like chains running in the direction of the crystallographic *b*-axis. Within this chain structure, neighboring molecules are linked by an O-H∙∙∙O bond between the oxime H atom (H2) and the carbonyl O atom (O1) [*d*(H∙∙∙O) = 1.76(6) Å, ∠(O-H∙∙∙O) = 174(5)°], as well as an N-H∙∙∙N bond involving the amide H atom (H1) and the N atom (N2) of the oxime group [*d*(H∙∙∙N) = 2.00(5) Å, ∠(N-H∙∙∙N) = 157(5)°], thus creating a cyclic motif that can be described by the graph set R_2_^2^(7). These one-dimensional supramolecular aggregates are stacked in the direction of the *a*-axis. Within this stacking order, the longitudinal displacement of consecutive molecules causes an effective overlap of the structurally different ring units (*Bzn* and *Lt*), showing a *Cg*∙∙∙*Cg* distance of 3.41 Å (see Figure 5c). This distance suggests the presence of intermolecular π∙∙∙π interactions [48,49,50] in the direction of the stacking axis. No other directional interactions between the molecules are evident, so that only van der Waals forces are present in the direction of the crystallographic *c*-axis.

### 2.2. Crystal Structure of 5-Chloro-1H-isatin-3-oxime [Structure **2**-(I)]

**Figure 6:**
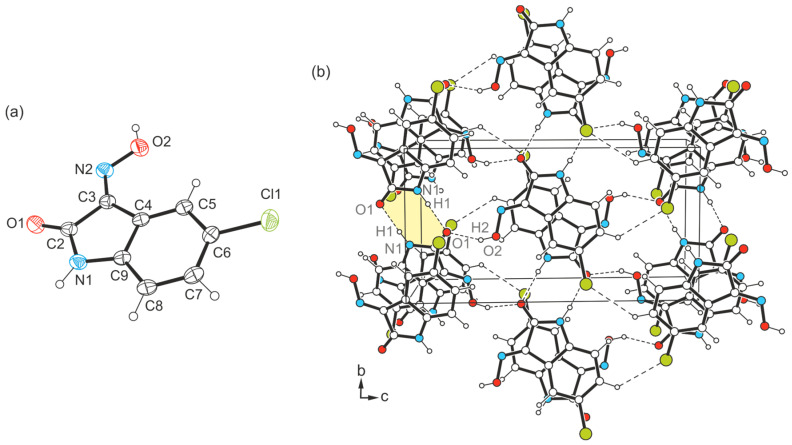
(**a**) Perspective view of the molecular structure of **2**. The displacement ellipsoids of the atoms are shown at a 50% probability level. (**b**) Packing diagram of **2**-(I) viewed in direction of the *a*-axis. Hydrogen bonds are shown as dashed lines. The 8-membered supramolecular ring motif is marked by color highlighting.

Compound **2**, which is equipped with a chlorine atom, crystallizes in the monoclinic space group *P*2_1_/*n* (*Z* = 4) and its molecular structure is shown in Figure 6a. This compound is reported in the literature and has already been characterized by Gervini et al. [51]. However, for the sake of consistency when comparing the co-ordination behavior of the molecules in the crystal structures of halogeno-substituted isatins **2**–**4**, compound **2** was crystallized by us under the same conditions as the bromine- and iodine-substituted analogues. The experimental data obtained (see Table S2) were taken into account in the analysis described below and yielded a structure model that is very similar to that described in the literature [51].

**Figure 7:**
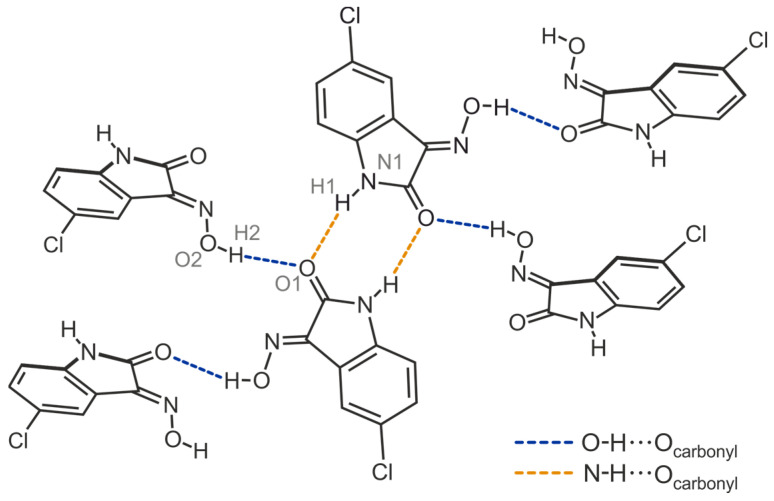
Mode of hydrogen bonding in the crystal structure of **2** including the labeling of co-ordinating atoms.

The smallest supramolecular entity of the crystal structure **2**-(I) consists of an inversion-symmetric dimer in which the molecules are linked via N-H∙∙∙O bonding involving the amine H atom and the carbonyl O atom [*d*(H∙∙∙O) = 1.96(3) Å, ∠(N-H∙∙∙O) = 167(3)°] (see Figure 6b and Figure 7), resulting in the formation of a cyclic motif of the structure R_2_^2^(8). The molecular dimers are linked to each other by O_oxime_-H∙∙∙O bonds of the pattern *C*(6) [*d*(H∙∙∙O) = 1.86(4) Å, ∠(O-H∙∙∙O) = 164(3)°], so that the carbonyl O atom acts as a bifurcated binding site within the supramolecular network. The structure motif shown in Figure 7 illustrates the bonding pattern of hydrogen bonds in the crystal structure of **2**. The distance of 2.88 Å between the chlorine atom and the arene H atom H7 of the neighboring molecule [∠(C-H∙∙∙Cl) = 134°] indicates the presence of a weak C-H∙∙∙Cl hydrogen bond [52]. The excerpt of the crystal structure shown in Figure 6b suggests that stacking forces exist between the molecules in the direction of the crystallographic *a*-axis. However, the lateral displacement of about 1.5 Å between the arene rings of consecutive molecules indicates the presence of only weak π∙∙∙π interactions.

### 2.3. Crystal Structures of 5-Bromo-1H-isatin-3-oxime (**3**)

#### 2.3.1. Polymorph **3**-(I)

**Figure 8:**
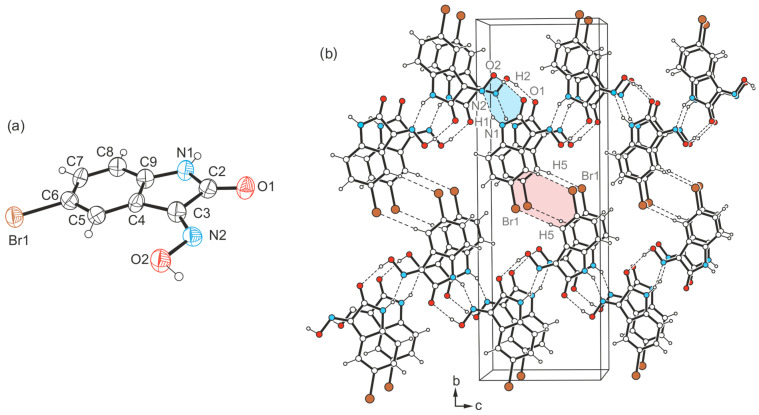
(**a**) Perspective view of the molecular structure of **3**. The displacement ellipsoids are drawn at a 40% probability level. (**b**) Packing diagram of **3**-(I) viewed down the crystallographic *a*-axis. Dashed lines represent hydrogen-bonding interactions. Supramolecular synthons are marked by color highlighting.

Crystal growth of the bromine-substituted compound **3** from 1,2-dimethoxyethane yields yellow needles of the space group *P*2_1_/*n* (*Z* = 4) with one molecule in the asymmetric unit (see Figure 8a). Unlike the previously described case, the present crystal structure consists of zigzag-like strands (Figure 8b), in which the molecules are linked via O-H_oxime_∙∙∙O=C [*d*(H∙∙∙O) = 1.94(14) Å, ∠(O-H∙∙∙O) = 172(16)°] and N-H∙∙∙N_oxime_ bonds [*d*(H∙∙∙N) = 2.02(10) Å, ∠(N-H∙∙∙N) = 153(10)°]. In total, the pattern of strong hydrogen bonds resembles that found in the crystal structure of **1**.

The bromine atom of the molecule causes weak cross-linking of the molecular chains via C-H∙∙∙Br bonds [*d*(H∙∙∙Br) = 3.04 Å, ∠(C-H∙∙∙Br) = 170°], so that the crystal structure of the present polymorph is composed of slightly corrugated molecular layers extending parallel to the crystallographic (*101*) plane. The centroid-to-centroid distance of 3.792 Å and the lateral displacement of ca. 1.7 Å between the molecules of consecutive layers indicate the presence of weak stacking forces that stabilize the crystal structure along the *a*-axis.

#### 2.3.2. Polymorph **3**-(II) 

The yellow needle-like crystals of **3** obtained from DMSO were found to be another polymorph of this compound. The crystal structure was solved in the monoclinic system (space group *P*2_1_/*c*) with two independent molecules (A and B) in the asymmetric unit of the cell. 

**Figure 9:**
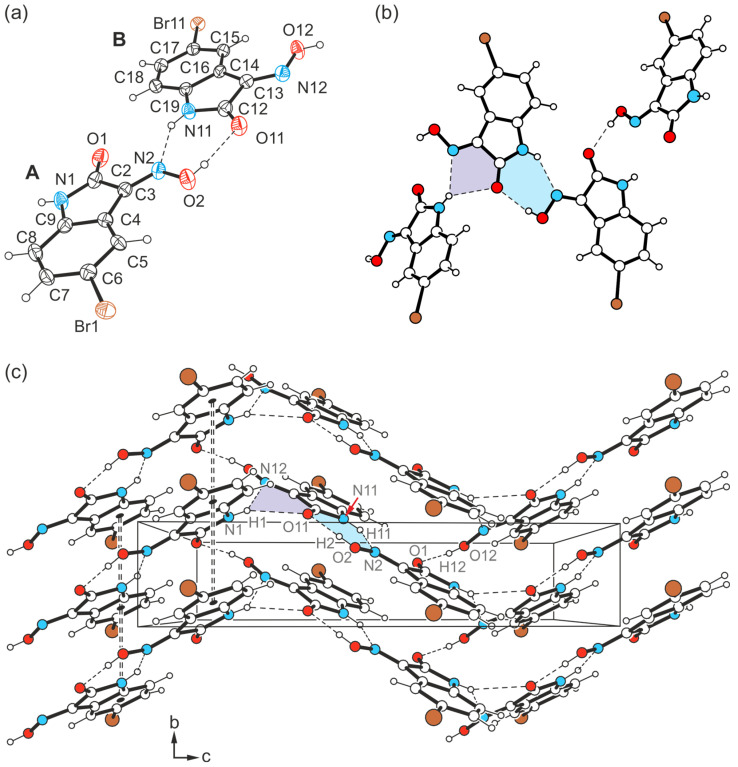
(**a**) Perspective view of molecules A and B of the crystal structure **3**-(II), linked by the R_2_^2^(7) synthon. Displacement ellipsoids of the atoms are drawn at a probability level of 50%. (**b**) Cyclic hydrogen bond synthons R_2_^2^(7) (marked in blue) and R_1_^2^(5) (violet). (**c**) Packing diagram of **3**-(II) viewed down the crystallographic *a*-axis. Hydrogen bonds are displayed as dashed lines, π∙∙∙π stacking interactions as dashed double lines.

The pattern of non-covalent intermolecular bonding in the crystal fundamentally differs from that of the previously described polymorph. In the present case, the two crystallographically non-equivalent molecules contribute in different ways to the formation of the supramolecular network. While the oxime group of the molecule labeled A and the lactam unit of molecule B again form the planar R_2_^2^(7) synthon [*d*(H∙∙∙O) = 1.95(5) Å, ∠(O-H∙∙∙O) = 170(5)°; *d*(H∙∙∙N) = 2.18(4) Å, ∠(N-H∙∙∙N) = 142(3)°], consecutive molecular pairs adopt a twisted arrangement (Figure 9a,c). This still allows the association of the oxime H atom of molecule B and the carbonyl O atom of molecule A [*d*(H∙∙∙O) = 1.87(5) Å, ∠(O-H∙∙∙O) = 168(5)°], yet introduces the cyclic synthon R_1_^2^(5) depicted in Figure 9b into the crystal architecture. It is based on hydrogen bonds from the amide H atom (H1) of the isatin molecule A to the carbonyl O atom (O11) and to the oxime N atom (N12) of the isatin molecule B [*d*(H∙∙∙O) = 2.48(5) Å, ∠(O-H∙∙∙O) = 141(4)°; *d*(H∙∙∙N) = 2.34(3) Å, ∠(N-H∙∙∙N) = 146(5)°]. 

As a result, the atoms H1 and O11 act as bifurcated bonding sites within the supramolecular network, rearranging the chainlike patterns incorporated in polymorph I. Instead, in the present case, the strong hydrogen bonds form complex two-dimensional networks that extend parallel to the crystallographic *bc*-plane according to Figure 9c. Within these structural domains, the aromatic units and the bromine atoms connected with them are located in the peripheral regions.

**Figure 10:**
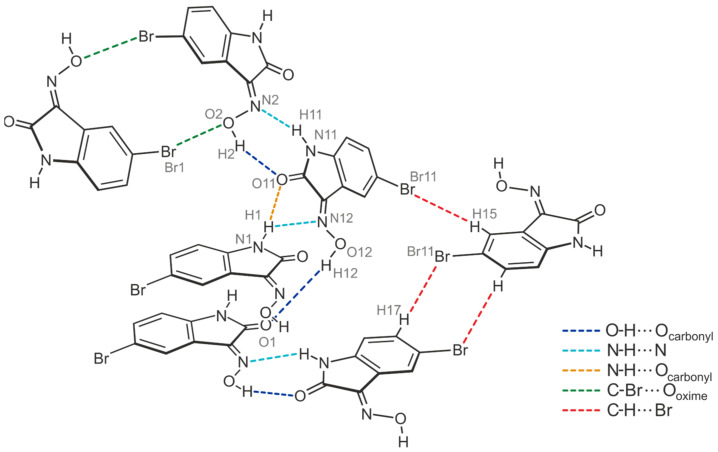
Connection mode of the molecules in the crystal structure **3**-(II).

Consequently, the somewhat weaker intermolecular interactions based on halogen atoms determine the crystalline assembly in the direction of the *a*-axis. The bromine atoms of the two molecules contribute in different ways to the molecular cross-linking. The distance between the bromine atom Br1 and the oxime O atom O2 [*d*(Br···O) = 3.183(2) Å] of two symmetry-equivalent isatin molecules A, as well as the well-defined bond geometry [∠(C-Br···O) = 164.1(1)°], indicate a stabilizing halogen bond (Table 1). In comparison, the distance of 3.465(2) Å between the corresponding atoms Br11 and O12 of two isatin molecules B is significantly longer and this value lies beyond the limit given by the van der Waals criterion (3.37 Å) [53]. The C-Br∙∙∙O angle of 134.2(1)° also deviates considerably from a linear arrangement of the atoms. Instead, the bromine atom of isatin molecule B is involved in the formation of weak C_arene_-H∙∙∙Br bonds [*d*(H∙∙∙Br) = 3.02, 3.05 Å, ∠(C-H∙∙∙Br) = 169, 131°]. A schematic representation showing the pattern of non-covalent intermolecular bonding in the crystal of polymorph II is shown in Figure 10. The view of the crystal structure along the *a*-axis (Figure 9c) conveys a herringbone-like packing structure, which is stabilized by π∙∙∙π stacking forces of the molecules, in addition to the hydrogen bonds discussed above.

### 2.4. Crystal Structures of 5-Iodo-1H-isatin-3-oxime (**4**)

#### 2.4.1. Polymorph **4**-(I)

**Figure 11:**
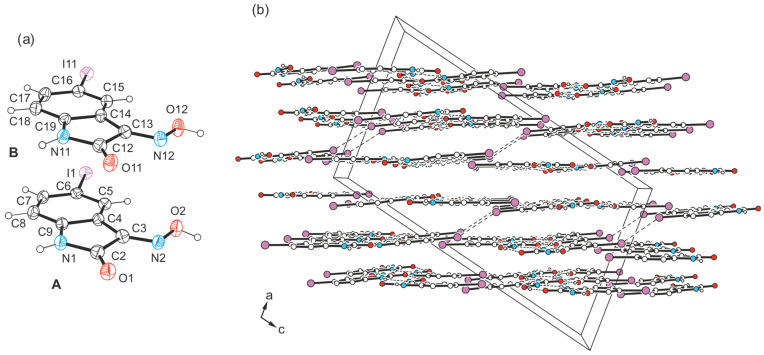
(**a**) Perspective view of the non-equivalent molecules A and B in structure **4**-(I). Displacement ellipsoids of the atoms are drawn at a 50% probability level. (**b**) Packing diagram of **4**-(I) viewed in direction of the *b*-axis. Dashed lines represent hydrogen bonds as well as I∙∙∙I interactions.

The yellow crystals of compound **4** obtained from acetone exhibit the space group *C*2/*c* with two independent molecules (A and B) in the asymmetric unit of the unit cell (see Figure 11a). The crystal structure is composed of infinite molecular 1D aggregates extending along the crystallographic *b-*axis. Therein, crystallographically independent molecules are arranged in an alternating order and connected among each other by O-H∙∙∙O [*d*(H∙∙∙O) = 1.96(5), 1.86(5) Å, ∠(O-H∙∙∙O) = 162(7), 171(6)°] and N-H∙∙∙N bonds [*d*(H∙∙∙N) = 2.23(5), 2.07(5) Å, ∠(N-H∙∙∙N) = 139(5), 146(5)°], following the R_2_^2^(7) motif. The structure of these aggregates is identical to that found in the crystal structure of polymorph I of the bromine-substituted compound **3**.

Viewing the crystal structure as shown in Figure 11b reveals a layered packing of molecules. Interlayer association is accomplished by I∙∙∙I interactions [type I; *d*(I∙∙∙I) = 3.772(1) Å, *θ*_1_ = *θ*_2_ = 141.1(1)°] between inversion-related molecules A. This bond geometry is unusual for an I∙∙∙I bond [54], since a type II bond geometry is usually observed for this halogen atom, in which the two angles *θ*_1_ (C_x_-I∙∙∙I) and *θ*_2_ (I∙∙∙I-C_y_) ideally assume values of 180° and 90° (halogen bonds of type I and II are, for example, defined in [55,56]). At this point, the theoretical studies carried out by Ibrahim et al. [57] are worth mentioning, in which the ability of C_6_H_5_X monomers to participate in X∙∙∙X interactions of different types was elucidated. The color-coded E_binding_ maps generated based on potential energy surface (PES) scans revealed that the binding energies of type I interactions increase with the increasing size of the σ-hole of the halogen atoms in the order C_6_H_5_Cl∙∙∙ClC_6_H_5_ < C_6_H_5_Br∙∙∙BrC_6_H_5_ < C_6_H5I∙∙∙IC_6_H_5_. This result is in agreement with previously reported results (see ref. [56]). 

**Figure 12:**
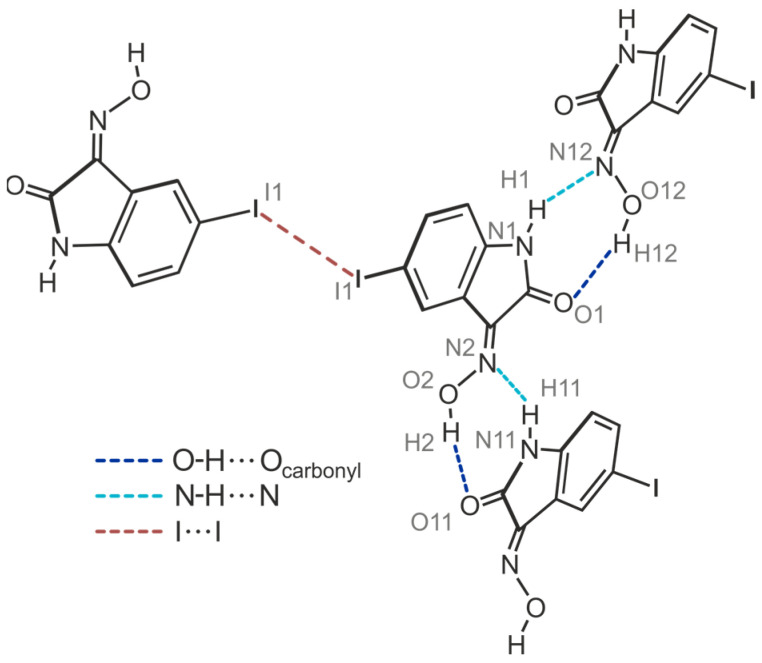
Mode of non-covalent intermolecular bonding in the crystal structure **4**-(I).

Figure 12 illustrates the interplay of the aforementioned hydrogen bonds and the type I halogen contact in **4**-(I), while visualizing that the iodine atom of molecule B is excluded from intermolecular interactions.

**Figure 13:**
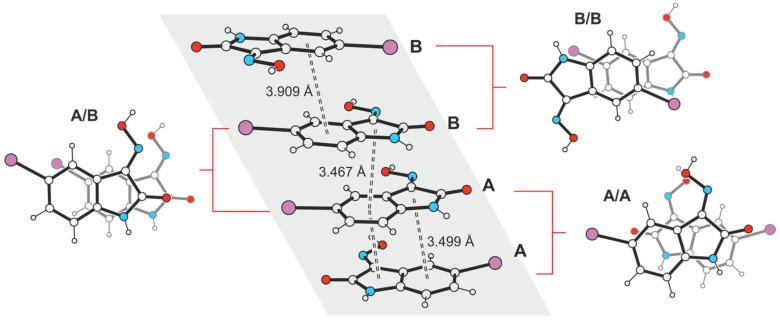
Stacking arrangement of the crystallographically independent molecules A and B in the crystal structure **4**-(I) (**middle**: perspective view of molecular stacking; **left**/**right**: top views of molecular pairs within the stacking structure). Oxygen atoms are marked as red, nitrogen as blue and iodine atoms as violet circles.

The columnar arrangement of the molecules in the direction of the layer normal indicates additional stabilization of the crystal structure by stacking forces. From the molecular arrangement shown in Figure 13, it is clear that these interactions occur both between ring units of different types (*Bzn*∙∙∙*Lt*) and between the benzene rings (*Bzn*∙∙∙*Bzn*) of isatin molecules.

Within the stacking structure, pairs of interacting molecules denoted A are arranged at an angle of ca. 150°, whereas pairs of molecules B adopt a nearly antiparallel orientation. Pairs of crystallographically independent molecules exhibit an offset arrangement along their longitudinal axis. The distances between the ring centers of interacting molecules cover a range of 3.467–3.909 Å, and the spatial dislocation between consecutive molecules is 0.571–1.536 Å. 

#### 2.4.2. Polymorph **4**-(II)

**Figure 14:**
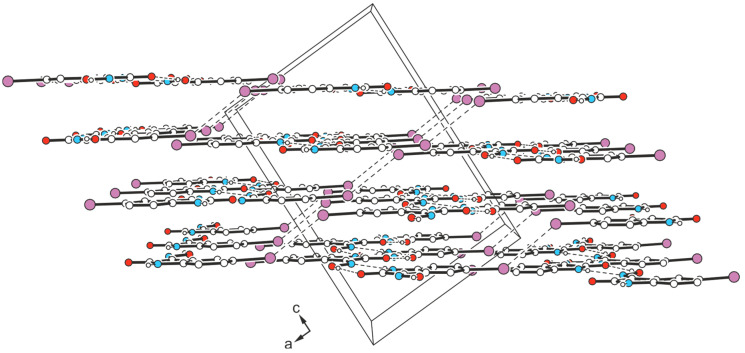
Packing diagram of **4**-(II) viewed in direction of the crystallographic *b*-axis. Dashed lines represent hydrogen bonds and I∙∙∙I interactions. Oxygen atoms are marked as red, nitrogen as blue and iodine atoms as violet circles.

The yellow block-like crystals of compound **4** obtained from ethanol represent another polymorph (polymorph II). This also exhibits the monoclinic space group *C*2/*c*, but with only one molecule in the asymmetric unit of the cell. The present crystal structure shows, once again, the formation of molecular zigzag chains characteristic of this type of compound, in which the molecules are linked by N-H∙∙∙N_oxime_ [*d*(H∙∙∙O) = 2.02(3) Å, ∠(O-H∙∙∙O) = 169(3)°] and O-H_oxime_∙∙∙O=C bonds [*d*(H∙∙∙N) = 2.10(3) Å, ∠(N-H∙∙∙N) = 147(3)°]. These aggregates arrange themselves into layers, which extend parallel to the crystallographic (*-102*) plane. Within a given layer, no directional interactions between molecular chains are apparent. The presence of a single molecule in the asymmetric unit implies a pattern of non-covalent bonds between the molecules that differs from polymorph I and, consequently, a different layer sequence, as can be seen from the packing diagram (Figure 14). 

**Figure 15:**
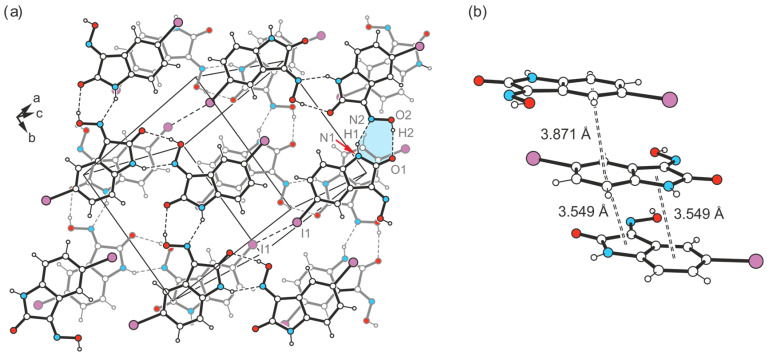
(**a**) Excerpt of the packing structure of **4**-(II) viewed in the direction of the layer normal. Broken lines represent hydrogen bonds and I∙∙∙I interactions. (**b**) Perspective view of the stacking arrangement of molecules in **4**-(II). Oxygen atoms are marked as red, nitrogen as blue and iodine atoms as violet circles.

The interconnection of different layers is based on symmetric I∙∙∙I type I contacts [*d*(I∙∙∙I) = 3.898(1) Å, *θ*_1_ = *θ*_2_ = 136.8(1)°] and π∙∙∙π interactions, the latter of which can be deduced from Figure 15. This view of the packing structure in the direction of the layer normal reveals a partial overlap between pairs of benzene units of consecutive molecules [*d*(*Cg*∙∙∙*Cg*) = 3.871(1) Å, slippage = 1.422 Å], as well as between the subsequent benzene and lactam moieties. 

#### 2.4.3. Polymorph **4**-(III) 

**Figure 16:**
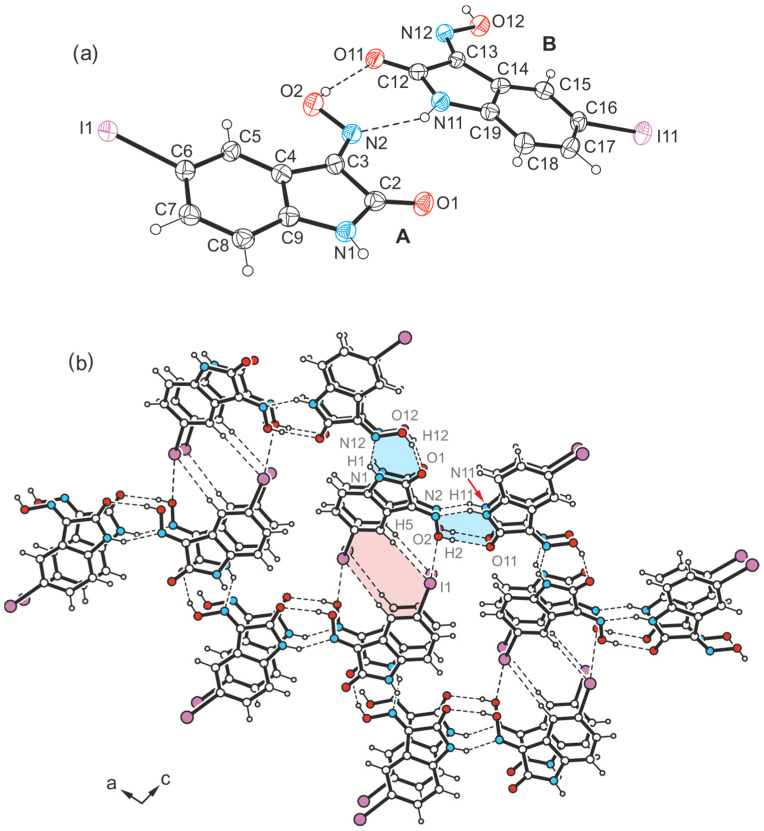
(**a**) Perspective view of the crystallographically independent molecules (A and B) of structure **4**-(III). Displacement ellipsoids of the atoms are shown at a 50% probability level. (**b**) Packing diagram of **4**-(III) viewed in direction of the crystallographic *b*-axis. Dashed lines represent hydrogen- and halogen-bond interactions. Cyclic synthons based on the former are highlighted in blue and red, respectively.

**Figure 17:**
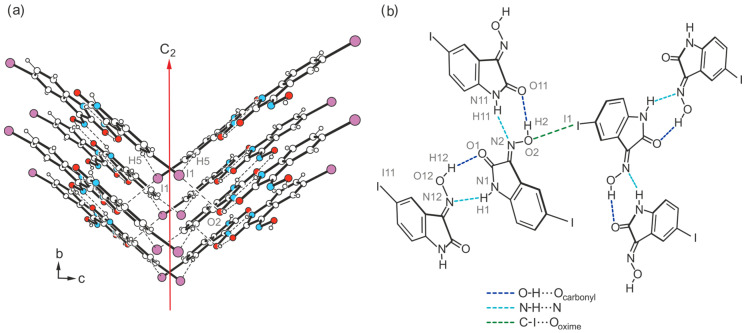
(**a**) Excerpt of the crystal structure **4**-(III) viewed along the *a*-axis. Oxygen atoms are marked as red, nitrogen as blue and iodine atoms as violet circles. (**b**) Pattern of non-covalent intermolecular bonding (dashed lines) in **4**-(III).

Crystal growth of **4** from a solvent mixture THF/H_2_O (*v*/*v* 1:1) yields a third polymorph in the form of yellow needles. However, this crystal structure present in the space group *C*2/*c* (*Z =* 16), containing two molecules in its asymmetric unit (A and B, see Figure 16a), is characterized by fundamental packing differences compared to the polymorphs I and II of this compound. It consists of one-dimensional supramolecular aggregates, in which crystallographically non-equivalent molecules are linked in alternating order by O-H∙∙∙O [*d*(H∙∙∙O) = 1.85(6), 2.01(5) Å, ∠(O-H∙∙∙O) = 163(5)°] and N-H∙∙∙N bonds [*d*(H∙∙∙N) = 2.14(6), 2.09(5) Å, ∠(N-H∙∙∙N) = 142(5), 146(5)°]. Unlike in the previously described polymorphs, these 1D aggregates do not form a layered crystal lattice. Instead, the molecular strands related by the two-fold symmetry axis are linked via I∙∙∙O_oxime_ halogen bonds, involving the iodine atom I1 and the oxime O atom O2 of isatin A [*d*(I∙∙∙O) = 3.383(3) Å, ∠(C-I∙∙∙O) = 169.3(1)°] (Figure 16b and Figure 17a). Therefore, this structure represents another example where hydrogen-bond-mediated molecular chains are interconnected by X···O_oxime_ contacts. In addition, iodine atom I1 is included in the formation of a C_arene_-H∙∙∙I bond [*d*(H∙∙∙I) = 3.21 Å, ∠(C-H∙∙∙I) = 173°]. The iodine atom I11 of isatin molecule B, on the other hand, does not feature a similar interaction with the oxime group of a neighboring molecule due to its distance of 3.733(1) Å from the atom O12. 

The molecular strands linked in this way are arranged at an angle of approximately 100°, so that the crystal structure viewed in the direction of the *a*-axis (see Figure 17a) reveals a herringbone-like packing of molecules. Stacking forces between the isatin molecules are also likely to contribute to the stabilization of the crystal structure in the present case. The stacking structures formed by molecules A and molecules B are largely identical and differ only in the *Cg*∙∙∙*Cg* distances between *Bzn* and *Lt* units, which amount to 3.630(2) Å for isatin molecules A and 3.520(2) Å for isatin molecules B. The spatial displacement of the molecules is 1.380 and 0.718 Å, respectively. A schematic representation of the pattern of non-covalent intermolecular bonding in the crystal is shown in Figure 17b.

#### 2.4.4. Polymorph **4**-(IV)

**Figure 18:**
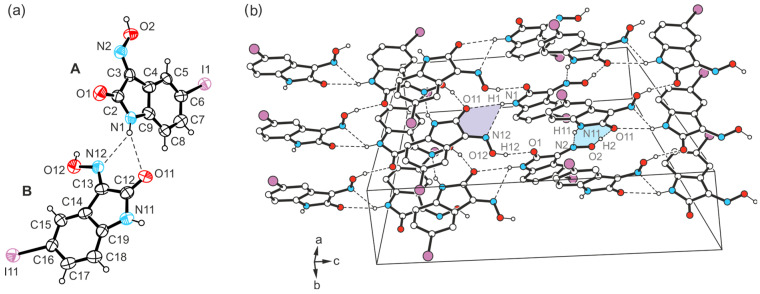
(**a**) Perspective view of molecules A (*x*, *y*, *z*) and B (*x*, 1 − *y*, −0.5 + *z*) of the crystal structure **4**-(IV), linked by the R_1_^2^(5) synthon. Displacement ellipsoids of the atoms are shown at the 40% probability level. (**b**) Excerpt of the crystal structure with hydrogen bonds marked as dashed lines. The cyclic supramolecular synthons R_2_^2^(7) and R_1_^2^(5) are highlighted in blue and purple, respectively.

Another crystal sample of the isatinoxime **4**, obtained from anhydrous THF in the form of yellow plates, proved to be an additional polymorph (polymorph IV) of this compound. The asymmetric unit of the crystal structure (space group *C*2/*c*) is occupied by two molecules (A and B, Figure 18a).

**Figure 19:**
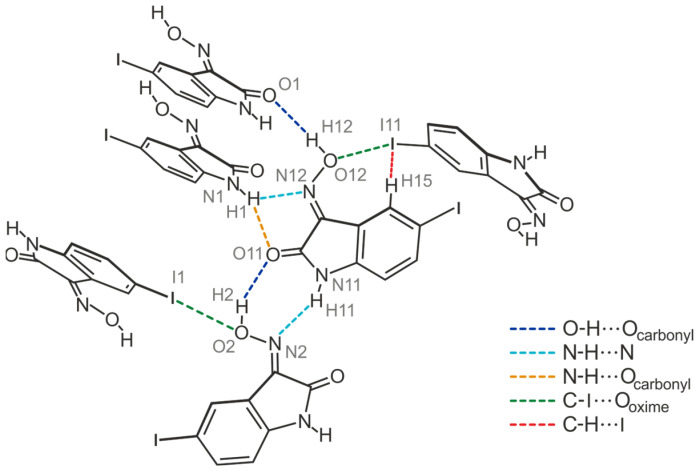
Structure motif showing the mode of non-covalent intermolecular bonding in the crystal structure **4**-(IV).

The strand-like linkage of the molecules characteristic of polymorphs **4**-(I), **4**-(II), and **4**-(III) is not present here. Instead, the hydrogen-bonding pattern is shared with polymorph II of the brominated compound **3**, as evidenced by the excerpt of the crystal structure shown in Figure 18b and the schematic representation in Figure 19. Accordingly, the functional groups of the two crystallographically independent molecules create different bonding patterns. The oxime group of isatin molecule A and the amide moiety of isatin molecule B form the characteristic synthon with the descriptor R_2_^2^(7) [*d*(H∙∙∙O) = 2.01(18) Å, ∠(O-H∙∙∙O) = 148(17)°; *d*(H∙∙∙N) = 2.03(14) Å, ∠(N-H∙∙∙N) = 158(14)°]. In contrast, the amide H atom H1 of isatin molecule A provides a bifurcated binding site to form a N-H∙∙∙O and a N-H∙∙∙N bond, in which the carbonyl O atom O11 and the oxime N atom N12 of isatin molecule B act as acceptors [*d*(H∙∙∙O) = 2.41(5) Å, ∠(N-H∙∙∙O) = 151(11)°; *d*(H∙∙∙N) = 2.55(14) Å, ∠(N-H∙∙∙N) = 120(12)°; see Figure 18]. The oxime H atom H12 of this molecule forms an O-H∙∙∙O bond to the carbonyl O atom O1 of molecule A [*d*(H∙∙∙O) = 1.84(8) Å, ∠(O-H∙∙∙O) = 167(16)°]. 

The iodine atoms are also involved in the cross-linking of the molecules, albeit in almost identical ways. As can be seen in Figure 19, the iodine atom I1 is associated with the oxime O atom O2 [*d*(I∙∙∙O) 3.287(9) Å, ∠(C-I∙∙∙O) 173.4(4)°], while iodine atom I11 acts as a bifurcated binding site for a C-I∙∙∙O and a weak C-H∙∙∙I bond. Bonding partners for this iodine atom are the oxime O atom O12 [*d*(I∙∙∙O) = 3.285(9) Å, ∠(C-I∙∙∙O) = 168.8(4)°] and the arene H atom H15 [*d*(H∙∙∙I) 3.19 Å, ∠(C-H∙∙∙I) 132°] of an adjacent isatin molecule. The sections from the crystal structures of polymorphs III and IV shown in Figure S2 (Supporting Information) illustrate the differences in their packing structures.

### 2.5. Hirshfeld Surface Analysis

Hirshfeld surface (HS) analysis [58,59] represents an effective way to gain insight into the co-ordination behavior of molecules in the crystalline state. In order to visualize and quantify the different types of intermolecular interactions [60] (hydrogen and halogen bonds, arene-based interactions, etc.), the molecular Hirshfeld surfaces mapped over *d*_norm_ and the associated two-dimensional fingerprint plots [46,61] were constructed using the *CrystalExplorer* program (Version 21.5) [62,63]. The normalized contact distance (*d*_norm_) based on the distances *d*_e_ and *d*_i_ can be expressed by the equation *d*_norm_ = [(*d*_i_−*r*_i_^vdw^)/*r*_i_^vdw^] + [(*d*_e_−*r*_e_^vdw^)/*r*_e_^vdw^], where *d*_e_ is the distance of the Hirshfeld surface from the nearest nucleus outside the surface and *d*_i_ the corresponding distance to the nearest nucleus inside the surface; and *r*^vdw^ is the van der Waals (vdW) radius of the atom [53]. In the two-dimensional fingerprint diagrams, the distance *d*_e_ is plotted against *d*_i_, where more frequently occurring pairs of values are characterized by lighter colors (from blue to green to yellow). 

To visualize the intermolecular interactions in the *d*_norm_ map, a color scale is used as well [63]. The red spots on the HS (negative *d*_norm_ values) represent contacts with distances shorter than the sum of the van der Waals radii, while the blue (positive *d*_norm_ values) and white regions (*d*_norm_ = 0) indicate contacts with distances longer than and equal to the sum of the van der Waals radii, respectively. 

**Figure 20:**
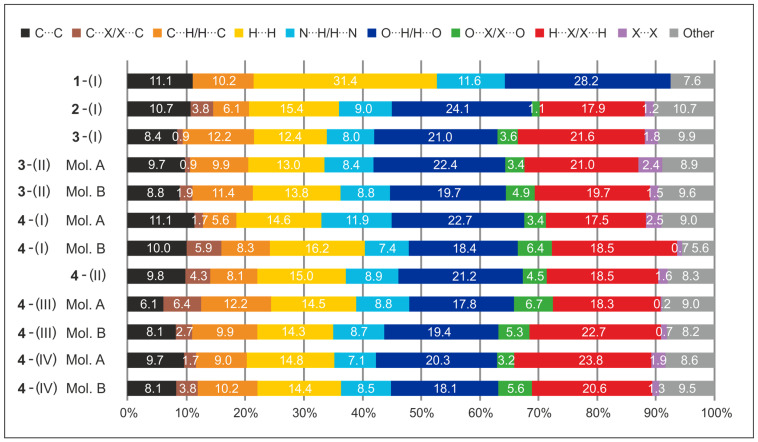
Histogram showing the relative contributions (in %) of the different types of intermolecular interactions to the respective Hirshfeld surfaces in the crystal structures of **1**–**4**.

The relative contributions (in %) of the different types of intermolecular interactions in the crystals of compounds **1**–**4** are displayed in Figure 20. The overall two-dimensional fingerprint plots and the diagrams showing the contributions of different contacts to the Hirshfeld surface can be found in Figures S3–S14 of the Supplementary Material. For the polymorphs of compounds **3** and **4**, views of the HS mapped over the shape index are shown in Figures S15–S20, while views of the HS plotted over the calculated electrostatic potential are presented in Figure S21.

#### 2.5.1. Hirshfeld Surface Analysis of the Crystal Structures of **1** and **2**

**Figure 21:**
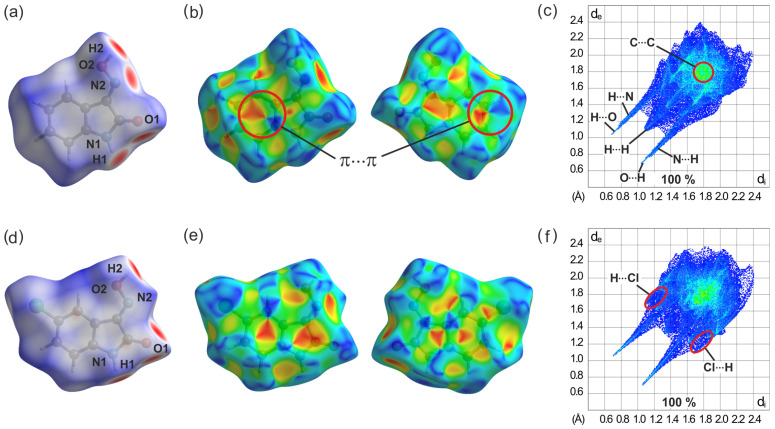
(**a**) Hirshfeld surface plotted over *d*_norm_, shape-index (front and back side), and 2D fingerprint plot for overall interactions (including highlighted interaction types) for **1**-(I) (**a**–**c**) and **2**-(I) (**d**–**f**). The areas of the 2D fingerprint plots (**c**) and (**f**) marked by red circles represent π∙∙∙π stacking interactions and H∙∙∙Cl bonds, respectively.

In Figure 21a, which shows the *d_norm_* surface for compound **1**, the red spots around the atoms N2 and H2 of the oxime group, the carbonyl oxygen O1, and the amino hydrogen H1 are attributed to O-H∙∙∙O and N-H∙∙∙N hydrogen bonds. They are visible in the two-dimensional fingerprint plot as two pairs of sharp symmetrical spikes with a minimum value at *d*_e_ + *d*_i_ ≈ 1.75 Å and *d*_e_ + *d*_i_ ≈ 1.90 Å, respectively (see Figure 21c). The decomposition of the fingerprint plot reveals that H···H interactions represent the largest contribution (31.4%) to the Hirshfeld surface. They appear in the fingerprint plot as widely scattered points with a concentration at *d*_e_ + *d*_i_ ≈ 2.2 Å. The H···O/O···H interactions appear as the next largest region (28.2%) of the fingerprint plot, while H···N/N···H interactions cover 11.6% of the HS. The C···C contacts, which are assigned to π···π stacking interactions, occupy 11.1% of the Hirshfeld surface and appear as a blue–green area in the fingerprint plot at *d*_e_ + *d*_i_ = 3.6–3.8 Å. The presence of this kind of interaction can also be visualized by the shape index of the Hirshfeld surface, in which π···π stacking appears as neighboring red and blue triangles (Figure 21b). The blue triangles represent convex regions symbolizing the presence of ring carbon atoms of the molecule inside the surface, while the red triangles symbolize concave regions caused by carbon atoms of the overlying π-stacked molecule.

The presence of a chloro substituent in **2**-(I) induces a pattern of strong hydrogen bonds involving H1, H2, and O1, which is clearly visible on the molecular *d*_norm_ surface (see Figure 21d) and in which the oxygen atom acts a bifurcated binding site. The fingerprint plot of this compound in Figure 21f is dominated by H∙∙∙O/O∙∙∙H and H∙∙∙Cl/Cl∙∙∙H interactions which make up 24.1% and 17.9% of the Hirshfeld surface, respectively. The percentage contribution of H∙∙∙H contacts is found to be 15.4%. The decreased amount of H∙∙∙H contacts compared to the previous structure is in accordance with the replacement of a hydrogen atom with a halogen substituent. The C∙∙∙C contacts with a contribution of 10.7% are significant interactions in the crystal of this compound. 

#### 2.5.2. Hirshfeld Surface Analysis of the Polymorphs of **3**

**Figure 22:**
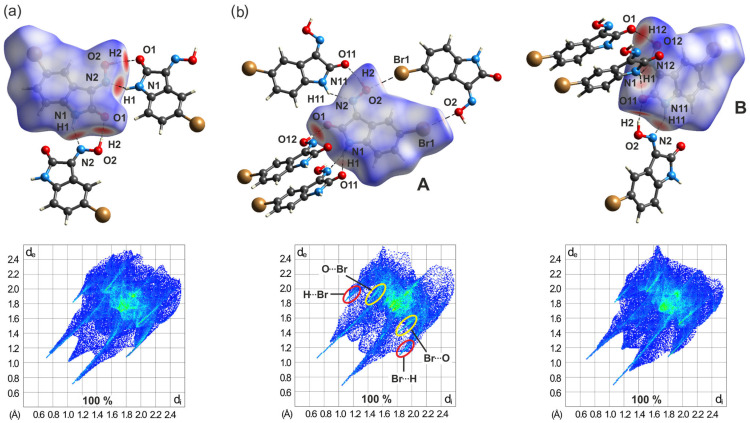
(**a**) *d*_norm_, selected intermolecular contacts, and two-dimensional fingerprint plots of **3**-(I) (**b**) co-ordination mode of molecule A (left) and molecule B (right) of **3**-(II). In the fingerprint plot of **3**-(II), Br∙∙∙O/O∙∙∙Br and H∙∙∙Br/Br∙∙∙H contacts are marked by yellow and red ellipses, respectively.

The comparison of Hirshfeld surfaces and fingerprint plots can be an effective tool for the identification of differences in the intermolecular interactions of the various polymorphs of a given molecular species. The bromo-substituted isatinoxime **3** exists in two polymorphic forms [designated **3**-(I) and **3**-(II)] with one and two molecules, respectively, in the asymmetric part of the unit cell. The molecular *d*_norm_ surfaces and the corresponding 2D-Hirshfeld plots are shown in Figure 22. The pattern of intermolecular hydrogen bonding in the polymorph **3**-(I) resembles that found for compound **1**, which is obvious from the similarities of the molecular HS plots (see Figure 22a). The halogen-based contacts, which comprise H∙∙∙Br/Br∙∙∙H, O∙∙∙Br/Br∙∙∙O, and Br∙∙∙Br interactions, contribute in summary to 27.0% of the Hirshfeld surface, while the contributions of H∙∙∙O/O∙∙∙H and H∙∙∙N/N∙∙∙H are 21.0 and 8.0%, respectively. The fingerprint plot for this polymorph also shows the presence of an unusually short H∙∙∙H contact (*d*_e_ + *d*_i_ ≈ 2.0 Å), with a length shorter than twice the van der Waals radius of hydrogen. Another detail of the 2D plot worth mentioning is the presence of a rather diffuse collection of blue points between the hydrogen-bonding spikes. This feature results from the cyclic hydrogen bond synthon [R_2_^2^(7)] connecting the molecules and arises from close H∙∙∙H contacts across the ring [64]. Similar patterns are also observed in the fingerprint plots of the related molecules discussed below. 

The crystallographically independent molecules of polymorph **3**-(II) exhibit different environments. These differences are also evident in the molecular Hirshfeld surfaces and in the 2D fingerprint plots shown in Figure 22b. The surface of the molecule labeled A is connected with three molecules B via O-H∙∙∙O and N-H∙∙∙N interactions. An additional molecule A related by inversion symmetry is associated by C-Br∙∙∙O_oxime_ halogen bonds. The latter interactions are represented in the fingerprint plot as bright green spikes (marked as yellow ellipses) with a shortest distance of *d*_e_ + *d*_i_ ≈ 3.2 Å. The molecule labeled B is surrounded only by three molecules A. Here, too, the linkage takes place via O-H∙∙∙O and N-H∙∙∙N bonds. The Br∙∙∙O streaks are also visible in the fingerprint plot of molecule B, but with an interatomic distance of 3.46 Å (vdW distance: 3.37 Å). The molecular Hirshfeld surfaces are dominated by H∙∙∙O/O∙∙∙H and H∙∙∙Br/Br∙∙∙H contacts which contribute 22.4/19.7% and 21.0/19.7%, respectively, to the Hirshfeld surface. In a similar manner to the polymorph described previously, C∙∙∙C contacts, which essentially correspond to offset π···π interactions, exert a stabilizing influence on the crystal structure.

#### 2.5.3. Hirshfeld Surfaces of the Polymorphs of **4**

The crystalline polymorphs of 5-iodoisatin-3-oxime (**4**) exhibit the space group *C*2/*c* with *Z*′ = 1 for **4**-(II) and *Z*′ = 2 for the polymorphs designated as **4**-(I), **4**-(III), and **4**-(IV). The differences are evident in the packing structures and, consequently, should be reflected in the Hirshfeld surface analyses. Polymorphs **4**-(I) and **4**-(II) are characterized by layered packing structures, with differences in the order of the molecular layers resulting from the different number of formula units. 

**Figure 23:**
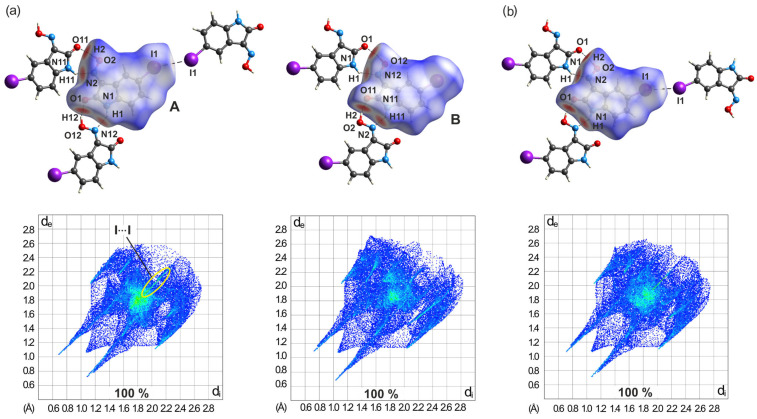
(**a**) *d*_norm_, selected intermolecular contacts, and two-dimensional fingerprint plots of molecules A (left) and B (right) of **4**-(I). The I···I contact is marked yellow. (**b**) *d*_norm_, selected intermolecular contacts, and two-dimensional fingerprint plot of **4**-(II).

In a similar manner to the structure of **3**-(II), a striking difference in the environment of the two molecules is also evident in the crystal structure of polymorph **4**-(I), which is due to a different number of their binding partners. Although the molecular *d*_norm_ plots shown in Figure 23a display similarities due to the chain-like association of the molecules via O-H∙∙∙O and N-H∙∙∙N bonding, in the molecule labeled A, there is another binding partner connected by an I∙∙∙I interaction of type I. This contact is indicated in the 2D fingerprint plot of the molecule by a bright streak (marked as a yellow ellipse) along the plot diagonal with a starting point at *d*_e_ + *d*_i_ ≈ 3.77 Å. An interaction of this type is not evident in the fingerprint plot of molecule B.

However, a comparative inspection of the two fingerprint plots reveals further differences. The pairs of spikes in the 2D plots corresponding to the present hydrogen bonds have different lengths for both the O-H∙∙∙O and the N-H∙∙∙N interactions. The fingerprint plot of molecule A reveals that, for each of these types of hydrogen bonds, the spike at the upper left for molecule A is significantly longer than that at the lower right, which is proof that the donor and acceptor molecules are not the same for these two interactions. A complementary donor/acceptor part is evident in the fingerprint plot of molecule B. 

Moreover, the 2D fingerprint plots of the two molecules indicate a different distribution of points around *d*_e_ ≈ *d*_i_ ≈ 1.80 Å, which suggests a different degree of overlap and spacing of the molecules within the stacking structure. 

The environment of the molecule in the crystal structure of polymorph **4**-(II) resembles that of molecule A in the previously described polymorph. These similarities are also obvious when comparing the 2D fingerprint plots of the molecules (see Figure S19 and Figure 23b). The molecular shape-index plot of **4**-(II) clearly indicates that the two faces of the molecule contribute in a different way in π∙∙∙π-stacking interactions in the crystal.

Different from the previously described polymorphs of compound **4**, H∙∙∙I/I∙∙∙H contacts provide the largest contribution to the molecular Hirshfeld surfaces of the polymorphs **4**-(III) and **4**-(IV), namely, 18.3/22.7% and 23.8/20.6%, respectively. With a slightly lower contribution, H∙∙∙O/O∙∙∙H contacts are the next prominent interactions (17.8/19.4% and 20.3/18.1%, respectively). H∙∙∙N/N∙∙∙H contacts contribute only 8.8/8.7% and 7.1/8.5%, respectively, to the Hirshfeld surface of the molecules, while the corresponding shares of I···O/O···I interactions amount to 6.7/5.3% and 3.2/5.6%. It should be noted, at this point, that the low percentage of I∙∙∙O contacts does not reflect the strength and structure-directing effect that such an interaction has on the packing behavior of the molecules in the solid state.

**Figure 24:**
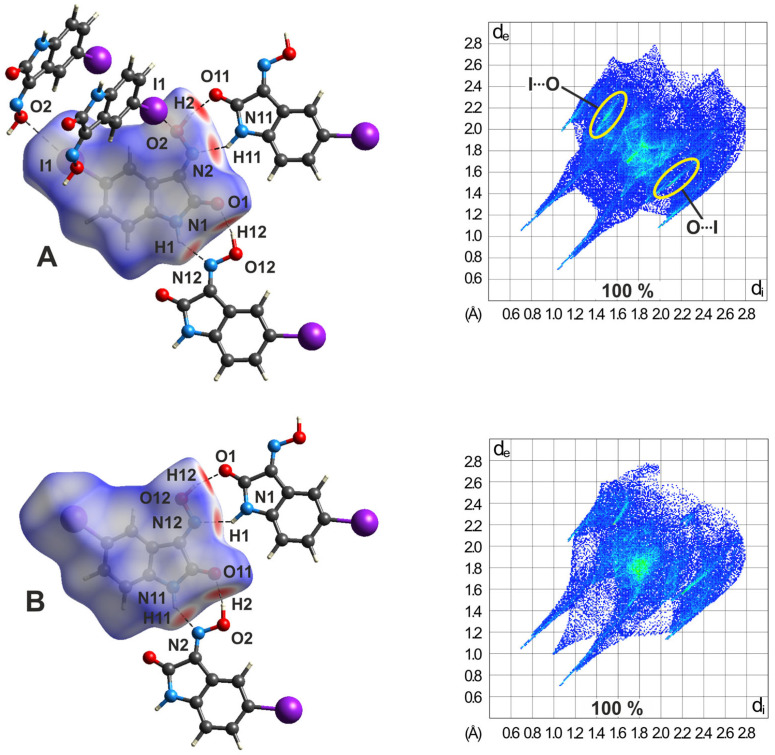
*d*_norm_, selected intermolecular contacts, and two-dimensional fingerprint plots of molecules A (top) and B (bottom) in **4**-(III). In the fingerprint plot of molecule A, I∙∙∙O/O∙∙∙I halogen bonds are marked by yellow ellipses.

The 3D HS plots of polymorph **4**-(III) shown in Figure 24 reveal striking differences with respect to the spatial environment of the two molecules. While the co-ordination of molecule A involves two molecules B by classical hydrogen bonds, as well as two other molecules A via I∙∙∙O bonding, the iodine atom of molecule B is excluded from any I∙∙∙O interactions. These differences are also evident in the 2D fingerprint plots of the molecules. The bright streaks emphasized by yellow ellipses in the fingerprint plot of molecule A show a shortest I∙∙∙O distance of *d*_e_ + *d*_i_ ≈ 3.38 Å, which is less than the sum of the vdW radii of the atoms. However, the corresponding streaks in the fingerprint plot of molecule B are shifted to higher values, in this case, with a shortest I∙∙∙O distance of 3.75 Å. The different proportions of C∙∙∙C contacts related to the Hirshfeld surfaces (6.1 vs. 8.1%) also indicate that the two molecules of the polymorph are involved to different degrees in the formation of π∙∙∙π stacking interactions.

**Figure 25:**
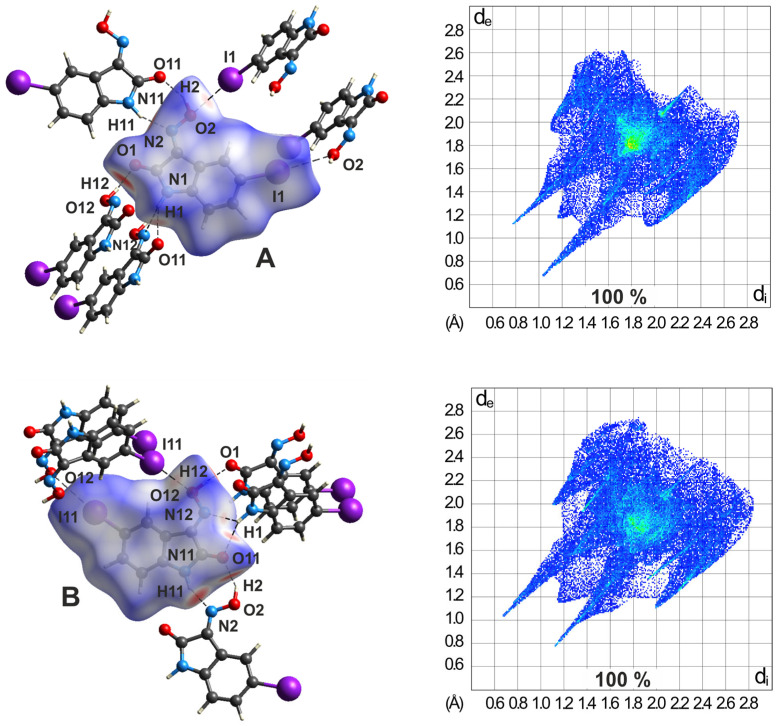
*d*_norm_, selected intermolecular contacts, and two-dimensional fingerprint plots of the molecules A (top) and B (bottom) in **4**-(IV).

The molecular 3D surfaces and corresponding fingerprint plots of polymorph **4**-(IV) shown in Figure 25 vividly illustrate the structural differences from polymorph **4**-(III). Corresponding to the larger number of binding partners for each of the two molecules in **4**-(IV), the molecular plots show a larger number of binding sites. This is due to the fact that each of the molecules is involved in the formation of weaker I∙∙∙O-type contacts, in addition to the expected strong hydrogen bonds.

The differences between polymorphs **4**-(III) and **4**-(IV) are also evident in the 2D fingerprint plots of the molecules. In a similar way as in the polymorph **4**-(I) mentioned above, the O-H∙∙∙O and N-H∙∙∙N bonds are visible by asymmetric pairs of spikes in the fingerprint plots. 

The differences are particularly noticeable for the H∙∙∙O bonds, as is clear from Figure S13b. For molecule A, the starting point of the spike on the left side of the plot diagonal (donor part) is at *d*_e_ + *d*_i_ = 1.13 + 0.78 Å, and on the right side of the diagonal (acceptor part) at *d*_e_ + *d*_i_ = 0.67 + 1.02 Å. The complementary donor/acceptor part is found in the fingerprint plot of molecule B (Figure S14b). The differences in spike lengths and thickness is even more pronounced for intermolecular H∙∙∙N interactions, as shown in Figures S13c and S14c, respectively. In this case, the asymmetry results from the presence of an N-H∙∙∙N and N-H∙∙∙O bond in which the amine H atom of molecule A acts as a bifurcated donor site, resulting in relatively long N∙∙∙H bond distances (2.55, 2.41 Å) and small bond angles (120, 151°). 

The two-dimensional fingerprint plots derived from the Hirshfeld surfaces allow the intermolecular interactions to be analyzed in detail so that even rather subtle differences between polymorphic systems can be quantified. They clearly indicate the different distribution of interactions for a single molecule in the four structures.

## 3. Conclusions

The analysis of the crystal structures of 5-halogeno-1*H*-isatin-3-oximes has shown that the oxime group of the bromine- and iodine-substituted derivatives is able to participate in the formation of halogen bonds. As assumed on the basis of the literature data, the oxime O atom and not the N atom acts as the acceptor for the halogen bonds. 

With regard to hydrogen bonding, the oxime functionality has proven its ability to compete with the other strong donor and acceptor sites. Apart from compound **2**, which contains R_2_^2^(8) synthons between two amide functionalities, all crystal structures feature cyclic R_2_^2^(7) units that connect amide and oxime moieties. The latter motif forms two different supramolecular assemblies. In the case of the structures **1**-(I), **3**-(I), **4**-(I), **4**-(II), and **4**-(III), the robust synthon R_2_^2^(7) yields 1-dimensional, flat, zigzag-like molecular chains with outward facing (if present) halogen atoms. Conversely, through the incorporation of R_1_^2^(5) synthons into the crystal framework of **3**-(II) and **4**-(IV), the aforementioned chains are cross-linked to more complex 2-dimensional networks, yet again with exposed halogen atoms.

**Figure 26:**
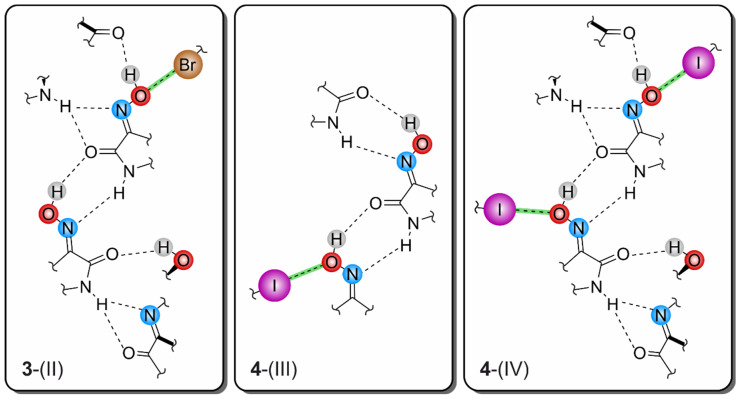
Halogen- and hydrogen-bonding motifs observed in the crystal structures **3**-(II), **4**-(III), and **4**-(IV).

Independent from their exact construction, these R_2_^2^(7)-mediated supramolecular patterns pack in a way that the halogen atoms are always oriented towards the oxime O atoms of neighboring isatin units. In the structures **3**-(II), **4**-(III), and **4**-(IV), this leads to clear X···O_Oxime_ halogen bonds as important secondary interactions. As they are invariably linked to the robust primary hydrogen bonds between the amide and oxime functionalities, hydrogen bonds and X···O_Oxime_ interactions display a distinct degree of complementarity in 5-haloisatin-3-oximes (see Figure 26 and Figure S22).

This is underlined by the fact that other forms of hydrogen and halogen bonds are virtually absent in the studied crystal structures, with the only exception being occasionally occurring, weak C-H···X contacts.

As is obvious from the description of the structures, all compounds exhibit stacking interactions in their crystalline solids. According to the dual nature of the isatin framework—the six-membered ring is electron-rich (donor), the five-membered ring electron-deficient (acceptor)—the dissimilar ring units of neighboring molecules exert an attractive electrostatic interaction within a molecular stacking arrangement. In most cases, the distance between the ring planes is 3.2–3.4 Å, which lies within the range of values typical for π∙∙∙π interactions. However, a comparative inspection of the crystal structures reveals striking differences with respect to the spatial arrangement between the molecules of a given stacking structure. The orientation of two molecules with respect to each other can assume a parallel or antiparallel longitudinal displacement, or can be antiparallel laterally offset. In the first two cases, the six- and five-membered rings of neighboring molecules are nearly congruent; in the third example, there is a partial overlap of the rings. An interesting example of this is the crystal structure of polymorph **4**-(I), in which these spatial relationships between molecules coexist. 

The above findings are supported by detailed Hirshfeld analyses of the individual crystal structures. The Hirshfeld surfaces not only reflect the large similarities in hydrogen bonding, but also reveal finer packing differences between different compounds and even polymorphs. This comprises, among others, the distinct degrees of halogen bonding within crystal structures of compound **4,** as well as the varied stacking arrangements between molecules in **4**-(I) and **4**-(II).

The knowledge gained on the basis of these investigations makes an important contribution to evaluating the potential of the oxime functionality in the field of supramolecular chemistry and crystal engineering.

## 4. Materials and Methods

All reactions were monitored via TLC on silica gel 60 F_254_ plates and halted when no traces of the educts were visible anymore. To the best of our knowledge, **4** has not yet been described in the scientific literature, while preparations of the remaining compounds have been introduced by various other groups (**1** [47,65,66,67] **2** [68,69,70], **3** [71,72,73]). The synthetic steps followed in this paper were based on procedures by Gabriel [74], as well as Schunck and Marchlewski [68].

Melting points, determined with a “Themovar 300429” hot stage microscope (Reichert, Vienna, Austria), are uncorrected. NMR data were collected with “Avance III-500 MHz” (Bruker, Billerica, MA, USA) and “ECZR 500 MHz” spectrometers (JEOL, Akishima, Japan), respectively, and use tetramethylsilane as internal standard. Elemental analyses were carried out with a “Vario Micro Cube" combustion analysis model (Elementar Analysensysteme, Langenselbold, Germany). For X-ray diffraction analysis, see below.

### 4.1. Crystal Structure Analysis

Crystals suitable for X-ray diffraction were obtained by slow evaporation of the solvent from a solution of the respective compound (26 different solvents were tested; see Tables S3 and S4). These crystals were identified under a microscope, mounted on a glass fiber, and analyzed in φ- and ω-scans with STOE equipment (image plate systems IPDS-2 and IPDS-2T, respectively), employing Mo-K_α_ radiation (λ = 0.71073 Å) monochromatized by graphite. Where not stated otherwise, diffraction data were collected at 160 K. Following data collection, indexing, and integration with the program X-AREA [75], data reduction including scaling and absorption correction was carried out via the X-RED and LANA program suite [76,77]. Preliminary structure solutions were obtained by direct methods (SHELXT2018/2 [78]), employing the program XSTEP-32 [79], and then refined by full-matrix least-squares calculations based on F^2^ for all reflections using the program SHELXL [80]. Aprotic hydrogen atoms were refined using a riding model, while protic hydrogen atoms were identified from the electron density map and, if need be, restrained to lie within common distances from their covalently bound N-/O-atoms [**1**-(I), **3**-(I), **4**-(I), and **4**-(IV)]. All atoms except riding hydrogen atoms were refined anisotropically. The graphical representation of the molecular structures was performed using the program ORTEP-III [81].

Hirshfeld surfaces were generated, analyzed, and visualized using the program CrystalExplorer (Version 21.5) [63]. Mapping of the electrostatic potential on the Hirshfeld surface employed the implemented routines B3LYP/6-31G(d,p) (**1**, **2**) and B3LYP/DGDZVP (**3**, **4**).

Crystallographic data for the structures described in this paper were deposited at the Cambridge Crystallographic Data Centre (CCDC) under identification numbers 2,313,454–2,313,461: 2,313,454 [**3**-(I)], 2,313,455 [**2**-(I)], 2,313,456 [**4**-(II)], 2,313,457 [**4**-(IV)], 2,313,458 [**4**-(III)], 2,313,459 [**1**-(I)], 2,313,460 [**4**-(I)], and 2,313,461 [**3**-(II)].

### 4.2. Synthesis of Compounds **1**–**4**

#### 4.2.1. Synthesis of Compounds **1**–**3**

The corresponding isatin (1*H*-isatin, 5-chloro-1*H*- and 5-bromo-1*H*-isatin) was dissolved in a boiling mixture of ethanol/tetrahydrofuran or ethanol/water. Afterwards, a solution of potassium carbonate (1 equiv) and hydroxylamine hydrochloride (1.5 equiv) in water was added. After refluxing or stirring at room temperature and diluting with water, the precipitating solid was filtered off, washed, recrystallized, and dried in vacuo. All details are given in Supporting Information.

*1H-isatin-3-oxime (***1***).* mp 244–245 °C; ^1^H NMR (DMSO-d_6_, 500 MHz) *δ* 6.88 (d, *J* = 8.0 Hz, 1H), 7.01 (t, *J* = 7.5 Hz, 1H), 7.35 (t, *J* = 7.8 Hz, 1H), 7.94 (d, *J* = 7.5 Hz, 1H), 10.71 (s, 1H), 13.32 (s, 1H) ppm; ^13^C NMR (DMSO-d_6_, 125 MHz) *δ* 110.3, 116.0, 122.2, 127.2, 132.2, 142.7, 144.3, 164.6 ppm; EA anal. C 59.08, N 17.40, H 3.72%, calcd for C_8_H_6_N_2_O_2_, C 59.26, N 17.28, H 3.73%. 

*5-Chloro-1H-isatin-3-oxime (***2***).* mp 260 °C (decomp.); ^1^H NMR (DMSO-d_6_, 500 MHz): *δ* 6.88 (d, *J =* 8.0 Hz, 1H), 7.40 (dd, *J =* 8.5, 2.0 Hz, 1H), 7.90 (d, *J =* 2.0 Hz, 1H), 10.84 (s, 1H), 13.63 (s, 1H) ppm; ^13^C NMR (125 MHz, DMSO-d_6_): *δ* 111.8, 117.1, 125.8, 126.3, 131.6, 141.4, 143.5, 164.2 ppm; EA anal. C 48.88, N 14.25, H 2.56%, calcd for C_8_H_5_ClN_2_O_2_, C 48.88, N 14.42, H 2.59%. 

*5-Bromo-1H-isatin-3-oxime (***3***).* mp 260 °C (decomp.) [**3**-(I) and **3**-(II)]; ^1^H NMR (DMSO-d_6_, 500 MHz): *δ* 6.84 (d, *J =* 8.5 Hz, 1H), 7.53 (dd, *J =* 8.5, 2.0 Hz, 1H), 8.03 (d, *J =* 2.5 Hz, 1H), 10.85 (s, 1H), 13.63 (s, 1H) ppm; ^13^C NMR (DMSO-d_6_, 125 MHz) *δ* 112.3, 113.4, 117.5, 129.1, 134.4, 141.8, 143.4, 164.1 ppm; EA anal. C 39.86, N 11.62, H 2.09%, calcd for C_8_H_5_BrN_2_O_2_, C 40.03, N 11.72, H 2.12%. 

#### 4.2.2. Synthesis of 5-Iodo-1*H*-isatin-3-oxime (**4**)

5-Iodoisatin (0.50 g, 1.83 mmol) was dissolved in a solution of tetrahydrofuran (20 mL) and ethanol (10 mL) at elevated temperature and a solution of potassium carbonate (0.25 g, 1.83 mmol) and hydroxylamine hydrochloride (0.20 g, 2.88 mmol) in water (15 mL) was added. After stirring the mixture at 50 °C for 3 h, it was concentrated under reduced pressure, the resulting solid washed thoroughly with water and chloroform, recrystallized from water/tetrahydrofuran (2:1 *v*/*v*), and dried in vacuo to obtain **4** as dark yellow crystals. Yield: 0.28 g (0.95 mmol, 52%). mp 255 °C (decomp.) [**4**-(I) and **4**-(II)], 250 °C (decomp.) [**4**-(III)] and 200 °C (decomp.) [**4**-(IV)]; ^1^H NMR (DMSO-d6, 500 MHz) *δ* 6.73 (d, *J =* 8.0 Hz, 1H), 7.67 (dd, *J =* 8.0, 2.0 Hz, 1H), 8.21 (d, *J =* 1.5 Hz, 1H), 10.82 (s, 1H), 13.58 (s, 1H) ppm; ^13^C NMR (DMSO-d6, 125 MHz) *δ* 84.7, 112.8, 118.0, 134.6, 140.1, 142.1, 143.2, 163.8 ppm; EA anal. C 33.36, N 9.73, H 1.75%, calcd for C_8_H_5_IN_2_O_2_, C 33.54, N 9.75, H 1.77%.

## Figures and Tables

**Table 1:** Overview of halogen bonds (displayed in bold) and strong hydrogen bonds in the crystal structures **3**-(II), **4**-(III), and **4**-(IV) (for further details, see Table S2).

Structure	Interaction			
	C-X∙∙∙O	*d*(X∙∙∙O)	*d*(C∙∙∙O)	∠(C-X∙∙∙O)
	D-H∙∙∙A	*d*(H∙∙∙O)	*d*(D···A)	∠(D-H∙∙∙A)
	(X = Br, I; D, A = N, O)	in Å	in Å	in °
**3**-(II)	**C(6)-Br(1)∙∙∙O(2)**	3.183(2)	5.038(3)	164.1(1)
	N(1)-H(1)···O(11)	2.48(5)	3.134(3)	141(4)
	N(1)-H(1)···N(12)	2.34(3)	3.030(4)	146(5)
	N(11)-H(11)···N(2)	2.18(4)	2.873(4)	142(3)
	O(2)-H(2)···O(11)	1.95(5)	2.724(3)	170(5)
	O(12)-H(12)···O(1)	1.87(5)	2.701(3)	168(5)
**4**-(III)	**C(6)-I(1)···O(2)**	3.383(2)	5.564(4)	169.4(1)
	N(1)-H(1)···N(12)	2.14(5)	2.901(4)	142(5)
	O(2)-H(2)···O(11)	2.01(6)	2.704(4)	163(5)
	N(11)-H(11)···N(2)	2.09(3)	2.833(4)	146(5)
	O(12)-H(12)···O(1)	1.85(6)	2.694(4)	163(5)
**4**-(IV)	**C(6)-I(1)···O(2)**	3.287(9)	5.390(14)	173.4(4)
	**C(16)-I(11)···O(12)**	3.285(9)	5.353(14)	168.8(4)
	N(1)-H(1)···N(12)	2.55(14)	3.081(17)	120(12)
	N(1)-H(1)···O(11)	2.41(5)	3.205(14)	151(11)
	N(11)-H(11)···N(2)	2.03(14)	2.859(19)	158(14)
	O(2)-H(2)···O(11)	2.01(18)	2.758(13)	148(17)
	O(12)-H(12)···O(1)	1.84(8)	2.665(13)	167(16)

## Data Availability

The data presented in this study are available upon request from the corresponding author.

## Supplementary Materials

The following supporting information can be downloaded at: https://www.mdpi.com/article/10.3390/molecules29051174/s1, Crystallographic and structure refinement data of the crystal structures of compounds **1**–**4**. (Table S1). Geometric parameters for non-covalent interactions in the crystal structures examined (Table S2). Results of crystallization experiments depending on the employed solvent(s) (Tables S3 and S4). Search in the Cambridge Structural Database (CSD): NOH_oxime_∙∙∙X hydrogen bonds (X = F, Cl, Br, I) (Figure S1). Comparison of the packing behaviors of polymorphs **4**-(III) and **4**-(IV) (Figure S2). 2D fingerprint plots for overall and individual interactions in the crystal packing of compounds **1**–**4** (Figures S3–S14). Hirshfeld surface plots of the crystal phases of compounds **3** and **4** (Figures S15–S20). Calculated electrostatic potential for compounds **1**–**4** mapped on the respective Hirshfeld surfaces (Figure S21). Overview of hydrogen and halogen bonding patterns in the crystal structures **1**-(I), **2**-(I), 3-(I), **3**-(II) and **4**-(I)–4-(IV) (Figure S22). ^1^H and ^13^C NMR spectra of compounds **1**–**4** (Figures 23–30). Synthetic procedures for **1**–**3** (further details) [82,83,84].

## References

[1] Cavallo G., Metrangolo P., Milani R., Pilati T., Priimagi A., Resnati G., Terraneo G. (2016). The Halogen Bond. Chem. Rev..

[2] Brown A., Beer P.D. (2016). Halogen Bonding Anion Recognition. Chem. Commun..

[3] Gilday L.C., Robinson S.W., Barendt T.A., Langton M.J., Mullaney B.R., Beer P.D. (2015). Halogen Bonding in Supramolecular Chemistry. Chem. Rev..

[4] Politzer P., Murray J.S. (2013). Halogen Bonding: An Interim Discussion. ChemPhysChem.

[5] Wolters L.P., Schyman P., Pavan M.J., Jorgensen W.L., Bickelhaupt F.M., Kozuch S. (2014). The Many Faces of Halogen Bonding: A Review of Theoretical Models and Methods. Wiley Interdiscip. Rev. Comput. Mol. Sci..

[6] Mukherjee A., Tothadi S., Desiraju G.R. (2014). Halogen Bonds in Crystal Engineering: Like Hydrogen Bonds yet Different. Acc. Chem. Res..

[7] Priimagi A., Cavallo G., Metrangolo P., Resnati G. (2013). The Halogen Bond in the Design of Functional Supramolecular Materials: Recent Advances. Acc. Chem. Res..

[8] Meyer F., Dubois P. (2013). Halogen Bonding at Work: Recent Applications in Synthetic Chemistry and Materials Science. CrystEngComm.

[9] Metrangolo P., Meyer F., Pilati T., Resnati G. (2008). Halogen Bonding in Supramolecular Chemistry. Angew. Chem. Int. Ed..

[10] Metrangolo P., Neukirch H., Pilati T., Resnati G. (2005). Halogen Bonding Based Recognition Processes: A World Parallel to Hydrogen Bonding. Acc. Chem. Res..

[11] Ivanov D.M., Bokach N.A., Kukushkin V.Y., Frontera A. (2022). Metal Centers as Nucleophiles: Oxymoron of Halogen Bond-Involving Crystal Engineering. Chem. Eur. J..

[12] Teyssandier J., Mali K.S., De Feyter S. (2020). Halogen Bonding in Two-Dimensional Crystal Engineering. ChemistryOpen.

[13] Sharber S.A., Mullin W.J., Thomas S.W. (2021). Bridging the Void: Halogen Bonding and Aromatic Interactions to Program Luminescence and Electronic Properties of π-Conjugated Materials in the Solid State. Chem. Mater..

[14] Sutar R.L., Huber S.M. (2019). Catalysis of Organic Reactions through Halogen Bonding. ACS Catal..

[15] Troff R.W., Mäkelä T., Topić F., Valkonen A., Raatikainen K., Rissanen K. (2013). Alternative Motifs for Halogen Bonding. Eur. J. Org. Chem..

[16] Takemura A., McAllister L.J., Karadakov P.B., Pridmore N.E., Whitwood A.C., Bruce D.W. (2014). Competition and Cooperation: Hydrogen and Halogen Bonding in Co-Crystals Involving 4-Iodotetrafluorobenzoic Acid, 4-Iodotetrafluorophenol and 4-Bromotetrafluorophenol. CrystEngComm.

[17] Robertson C.C., Perutz R.N., Brammer L., Hunter C.A. (2014). A Solvent-Resistant Halogen Bond. Chem. Sci..

[18] Pinfold H., Sacchi M., Pattison G., Costantini G. (2021). Determining the Relative Structural Relevance of Halogen and Hydrogen Bonds in Self-Assembled Monolayers. J. Phys. Chem. C.

[19] Rowe R.K., Ho P.S. (2017). Relationships between Hydrogen Bonds and Halogen Bonds in Biological Systems. Acta Crystallogr. Sect. B Struct. Sci. Cryst. Eng. Mater..

[20] Voth A.R., Khuu P., Oishi K., Ho P.S. (2009). Halogen Bonds as Orthogonal Molecular Interactions to Hydrogen Bonds. Nat. Chem..

[21] Vasylyeva V., Nayak S.K., Terraneo G., Cavallo G., Metrangolo P., Resnati G. (2014). Orthogonal Halogen and Hydrogen Bonds Involving a Peptide Bond Model. CrystEngComm.

[22] Riel A.M.S., Rowe R.K., Ho E.N., Carlsson A.C.C., Rappé A.K., Berryman O.B., Ho P.S. (2019). Hydrogen Bond Enhanced Halogen Bonds: A Synergistic Interaction in Chemistry and Biochemistry. Acc. Chem. Res..

[23] Decato D.A., Riel A.M.S., May J.H., Bryantsev V.S., Berryman O.B. (2021). Theoretical, Solid-State, and Solution Quantification of the Hydrogen Bond-Enhanced Halogen Bond. Angew. Chem. Int. Ed..

[24] Aakeröy C.B., Panikkattu S., Chopade P.D., Desper J. (2013). Competing Hydrogen-Bond and Halogen-Bond Donors in Crystal Engineering. CrystEngComm.

[25] Aakeröy C.B., Sinha A.S., Epa K.N., Chopade P.D., Smith M.M., Desper J. (2013). Structural Chemistry of Oximes. Cryst. Growth Des..

[26] Rubin-Preminger J.M., Englert U. (2009). Halogen Bonding in Substituted Cobaloximes. Inorg. Chim. Acta.

[27] Bedeković N., Martinez V., Topić E., Stilinović V., Cinčić D. (2020). Cobaloximes as Building Blocks in Halogen-Bonded Cocrystals. Materials.

[28] Mazik M., Buthe A. (2007). Oxime-Based Receptors for Mono- and Disaccharides. J. Org. Chem..

[29] Mazik M., Buthe A. (2008). Highly effective receptors showing Di- vs. Monosaccharide Preference. Org. Biomol. Chem..

[30] Bruton E.A., Brammer L., Pigge F.C., Aakeröy C.B., Leinen D.S. (2003). Hydrogen Bond Patterns in Aromatic and Aliphatic Dioximes. N. J. Chem..

[31] Etter M.C., MacDonald J.C., Bernstein J. (1990). Graph-set Analysis of Hydrogen-bond Patterns in Organic Crystals. Acta Crystallogr. Sect. B.

[32] Bernstein J., Davis R.E., Shimoni L., Chang N.L. (1995). Patterns in Hydrogen Bonding: Functionality and Graph Set Analysis in Crystals. Angew. Chemie Int. Ed..

[33] Maurin J.K. (1995). Resonance-Assisted Hydrogen Bonds Between Oxime and Carboxyl Groups. II. The Tetrameric Structure of Pyruvic Acid Oxime. Acta Crystallogr. Sect. C Cryst. Struct. Commun..

[34] Maurin J.K. (1998). Oxime-Carboxyl Hydrogen Bonds: The Preferred Interaction Determining Crystal Packing of “Carboxyoximes”. Acta Crystallogr. Sect. B Struct. Sci..

[35] Kubicki M., Borowiak T., Antkowiak W.Z. (2000). Hydrogen Bonds in “Carboxyoximes”: The Case of Bornane Derivatives. Z. Naturforsch. B.

[36] Maurin J.K. (1992). Structure of (*E*)-4-Benzoylbutyramide Oxime. Acta Cryst..

[37] Mazik M., Bläser D., Boese R. (2000). Programmed Construction of Discrete Self-Assembled Cyclic Aggregates. Chem. Eur. J..

[38] Mazik M., Bläser D., Boese R. (2005). α,ß -Unsaturated Ketoximes Carrying a Terminal Pyridine or Quinoline Subunit as Building Blocks for Supramolecular Syntheses. J. Org. Chem..

[39] Mazik M., Bläser D., Boese R. (1999). New Helical Hydrogen-Bonded Assemblies Forming Channel-Inclusion Complexes. Tetrahedron Lett..

[40] Aakeröy C.B., Beatty A.M., Leinen D.S. (2001). Syntheses and Crystal Structures of New “Extended” Building Blocks for Crystal Engineering: (Pyridylmethylene)Aminoacetophenone Oxime Ligands. Cryst. Growth Des..

[41] Groom C.R., Bruno I.J., Lightfoot M.P., Ward S.C. (2016). The Cambridge Structural Database. Acta Crystallogr. Sect. B.

[42] Bruno I.J., Cole J.C., Edgington P.R., Kessler M., Macrae C.F., McCabe P., Pearson J., Taylor R. (2002). New Software for Searching the Cambridge Structural Database and Visualizing Crystal Structures. Acta Crystallogr. Sect. B.

[43] Haleblian J., McCrone W. (1969). Pharmaceutical Applications of Polymorphism. J. Pharm. Sci..

[44] Dunitz J.D., Bernstein J. (1995). Disappearing Polymorphs. Acc. Chem. Res..

[45] Bernstein J., Davey R.J., Henck J.-O. (1999). Concomitant Polymorphs. Angew. Chem. Int. Ed..

[46] McKinnon J.J., Spackman M.A., Mitchell A.S. (2004). Novel Tools for Visualizing and Exploring Intermolecular Interactions in Molecular Crystals. Acta Crystallogr. Sect. B Struct. Sci..

[47] Mo X.L., Chen C.H., Liang C., Mo D.L. (2018). Copper-Catalyzed Carbonyl Group Controlled Coupling of Isatin Oximes with Arylboronic Acids To Prepare N-Aryloxindole Nitrones. Eur. J. Org. Chem..

[48] Dance I., Atwood J.L., Steed J.M. (2004). π-π Interactions: Theory and Scope. Encyclopedia of Supramolecular Chemistry.

[49] James S.L., Atwood J.L., Steed J.W. (2004). π-π Stacking as a Crystal Engineering Tool. Encyclopedia of Supramolecular Chemistry.

[50] Salonen L.M., Ellermann M., Diederich F. (2011). Aromatic Rings in Chemical and Biological Recognition: Energetics and Structures. Angew. Chem. Int. Ed..

[51] Martins B.B., Gervini V.C., Pires F.C., Bortoluzzi A.J., de Oliveira A.B. (2016). (3*Z*)-5-Chloro-3-(Hydroxyimino)Indolin-2-One. IUCrData.

[52] Steiner T., Desiraju G.R. (1999). The Weak Hydrogen Bond in Chemistry and Structural Biology.

[53] Bondi A. (1964). Van Der Waals Volumes and Radii. J. Phys. Chem..

[54] Saha B.K., Rather S.A., Saha A. (2016). Interhalogen Interactions in the Light of Geometrical Correction. Cryst. Growth Des..

[55] Pedireddi V.R., Reddy D.S., Goud B.S., Craig D.C., Rae A.D., Desiraju G.R. (1994). The Nature of Halogen...Halogen Interactions and the Crystal Structure of 1,3,5,7-Tetraiodoadamantane. J. Chem. Soc. Perkin Trans. 2.

[56] Awwadi F.F., Willett R.D., Peterson K.A., Twamley B. (2006). The Nature of Halogen...Halogen Synthons: Crystallographic and Theoretical Studies. Chemistry.

[57] Ibrahim M.A.A., Saeed R.R.A., Shehata M.N.I., Ahmed M.N., Shawky A.M., Khowdiary M.M., Elkaeed E.B., Soliman M.E.S., Moussa N.A.M. (2022). Type I–IV Halogen⋯Halogen Interactions: A Comparative Theoretical Study in Halobenzene⋯Halobenzene Homodimers. Int. J. Mol. Sci..

[58] Spackman M.A., Byrom P.G. (1997). A Novel Definition of a Molecule in a Crystal. Chem. Phys. Lett..

[59] McKinnon J.J., Mitchell A.S., Spackman M.A. (1998). Hirshfeld Surfaces: A New Tool for Visualising and Exploring Molecular Crystals. Chem. Eur. J..

[60] McKinnon J.J., Jayatilaka D., Spackman M.A. (2007). Towards Quantitative Analysis of Intermolecular Interactions with Hirshfeld Surfaces. Chem. Commun..

[61] Spackman M.A., McKinnon J.J. (2002). Fingerprinting Intermolecular Interactions in Molecular Crystals. CrystEngComm.

[62] Spackman M.A., Jayatilaka D. (2009). Hirshfeld Surface Analysis. CrystEngComm.

[63] Spackman P.R., Turner M.J., McKinnon J.J., Wolff S.K., Grimwood D.J., Jayatilaka D., Spackman M.A. (2021). CrystalExplorer: A Program for Hirshfeld Surface Analysis, Visualization and Quantitative Analysis of Molecular Crystals. J. Appl. Crystallogr..

[64] McKinnon J.J., Fabbiani F.P.A., Spackman M.A. (2007). Comparison of Polymorphic Molecular Crystal Structures through Hirshfeld Surface Analysis. Cryst. Growth Des..

[65] Baeyer A., Comstock W. (1883). Über Oxindol Und Isatoxim. Ber. Dtsch. Chem. Ges..

[66] Borsche W., Sander W. (1914). Untersuchungen Über Isatin Und Seine Derivate II: Isatoxim -> o-Cyan-Phenyl-Isocyanat Und Verwandte Reaktionen. Chem. Ber..

[67] Jeankumar V.U., Alokam R., Sridevi J.P., Suryadevara P., Matikonda S.S., Peddi S., Sahithi S., Alvala M., Yogeeswari P., Sriram D. (2014). Discovery and Structure Optimization of a Series of Isatin Derivatives as Mycobacterium Tuberculosis Chorismate Mutase Inhibitors. Chem. Biol. Drug Des..

[68] Schunck E., Marchlewski L. (1895). Zur Kenntniss Der Rothen Isomeren Des Indigotins Und Über Einige Derivate Des Isatins. Ber. Dtsch. Chem. Ges..

[69] Kearney T., Harris P.A., Jackson A., Joule J.A. (1992). Synthesis of Isatin-3-Oximes from 2-Nitroacetanilides. Synthesis.

[70] Wei W.T., Zhu W.M., Ying W.W., Wu Y., Huang Y.L., Liang H. (2017). Metal-Free Synthesis of Isatin Oximes: Via Radical Coupling Reactions of Oxindoles with t-BuONO in Water. Org. Biomol. Chem..

[71] Baeyer A., Knop C.A. (1866). Untersuchungen Über Die Gruppe Des Indigblaus. Justus Liebigs Ann. Chem..

[72] Jensen B.S., Jorgensen T.D., Ahring P.K., Christophersen P., Strobaek D., Teuber L., Olesen S.P. (2000). Use of Isatin Derivatives as Ion Channel Activating Agents. WO Patent.

[73] Zhu G.-D., Gandhi V.B., Gong J., Luo Y., Liu X., Shi Y., Guan R., Magnone S.R., Klinghofer V., Johnson E.F. (2006). Discovery and SAR of Oxindole–Pyridine-Based Protein Kinase B/Akt Inhibitors for Treating Cancers. Bioorg. Med. Chem. Lett..

[74] Gabriel S. (1883). Beitrag Zur Kenntnis Aromatischer Nitrosokörper. Ber. Dtsch. Chem. Ges..

[75] (2015). X-AREA.

[76] (2014). X-RED.

[77] (2015). LANA.

[78] Sheldrick G.M. (2015). SHELXT—Integrated Space-Group and Crystal-Structure Determination. Acta Crystallogr. Sect. A.

[79] (2000). XStep-32.

[80] Sheldrick G.M. (2015). Crystal Structure Refinement with SHELXL. Acta Crystallogr. Sect. C.

[81] Farrugia L.J. (2012). WinGX and ORTEP for Windows: An Update. J. Appl. Crystallogr..

[82] Spek A.L. (2020). checkCIF validation ALERTS: What they mean and how to respond. Acta Crystallogr. E.

[83] Golushko A.A., Sandzhieva M.A., Ivanov A.Y., Boyarskaya I.A., Khoroshilova O.V., Barkov A.Y., Vasilyev A.V. (2018). Reactions of 3,3,3-Trihalogeno-1-Nitropropenes with Arenes in the Superacid CF_3_SO_3_H: Synthesis of (Z)-3,3,3-Trihalogeno-1,2-diarylpropan-1-one Oximes and Study on the Reaction Mechanism. J. Org. Chem..

[84] Campbell A., Tasker P., Parsons S. (2015). CCDC 1413226: Experimental Crystal Structure Determination.

