# Supplementary material for: Combination of Hydrogen and Halogen Bonds in the Crystal Structures of 5-Halogeno-1H-isatin-3-oximes: Involvement of the Oxime Functionality in Halogen Bonding
Source: Molecules. 2024 Mar 6;29(5):1174. doi: 10.3390/molecules29051174 (PMC10934878; doi:10.3390/molecules29051174)
Supplement: Supplementary file 1 [file molecules-29-01174-s001.zip › molecules-2800272-supplementary.pdf]

Supporting Information

Combination of hydrogen and halogen bonds in the crystal structures of 5-halogeno-1*H*-isatin-3-oximes: Involvement of the oxime functionality in halogen bonding

Eric Meier, Wilhelm Seichter and Monika Mazik*

Institut für Organische Chemie, Technische Universität Bergakademie Freiberg, Leipziger Straße 29, 09596 Freiberg, Germany, Tel.: 03731392389; Fax: 03731393170

e-mail: monika.mazik@chemie.tu-freiberg.de; https://tu-freiberg.de/fakultaet2/orgch

- Crystallographic and structure refinement data of the crystal structures **1**-(I), **2**-(I), **3**-(I), **3**-(II), **4**-(I), **4**-(II), **4**-(III) and **4**-(IV) (**Table S1**).
- Geometric parameters for noncovalent interactions in the crystal structures examined (**Table S2**).
- Results of crystallization experiments depending on the employed solvent(s) (**Tables S3** and **S4**).
- Search in the Cambridge Structural Database (CSD): $NOH_{oxime}\cdots X$ hydrogen bonds (X = F, Cl, Br, I) (**Figure S1**).
- Comparison of the packing behaviors of polymorphs **4**-(III) and **4**-(IV) (**Figure S2**)
- 2D fingerprint plots for overall and individual interactions in the crystal packing of compounds **1-4** (**Figures S3-S14**).
- Hirshfeld surface plots of the crystal phases of compounds **3** and **4** (**Figures S15-S20**).
- Calculated electrostatic potential for compounds **1** – **4** mapped on the respective Hirshfeld surfaces (**Figure 21**)
- Overview of hydrogen and halogen bonding patterns in the crystal structures **1**-(I), **2**-(I), **3**-(I), **3**-(II) and **4**-(I) – **4**-(IV) (**Figure S22**).
- ^{1}H and ^{13}C NMR spectra of compounds **1-4** (**Figures S23-S30**).
- Synthetic procedures for **1-3** (further details)
- References

Table S1: Crystallographic and structure refinement data of the crystal structures **1**-(I), **2**-(I), **3**-(I) **3**-(II), **4**-(I), **4**-(II), **4**-(III) and **4**-(IV).

Crystal structure	**1**-(I)	**2**-(I)	**3**-(I)
Empirical formula	$C_8H_6N_2O_2$	$C_8H_5ClN_2O_2$	$C_8H_5BrN_2O_2$
Formula weight	162.15	196.59	241.05
Crystal system	monoclinic	monoclinic	monoclinic
Space group	$P\ 2_1$	$P\ 2_1/c$	$P\ 2_1/n$
a (Å)	3.8808(7)	7.3643(4)	3.7915(4)
b (Å)	10.3169(11)	7.5180(3)	25.324(3)
c (Å)	9.1690(15)	14.2546(7)	8.5580(8)
α(°)	90	90	90
β(°)	93.516(14)	93.934(4)	100.659(8)
γ (°)	90	90	90
V (Å^3)	366.42(10)	787.34(7)	807.53(15)
Z	2	4	4
F(000)	168	400	472
D_c (Mg m^{-3})	1.470	1.658	1.983
μ (mm^{-1})	0.109	0.446	5.052
Data collection			
Temperature (K)	160(2)	160(2)	160(2)
No. of collected reflections	8106	7980	4389
within the Θ-limit (°)	3.0 – 27.0	2.8 – 27.0	2.9 – 26.0
Index ranges $\pm h$, $\pm k$, $\pm l$	-4/4, -13/13, -11/11	-9/9, -9/9, -16/18	-4/3, -31/31, -10/10
No. of unique reflections	834 [b]	1723	1589
R_{int}	0.0344 [b]	0.0400	0.1043
Refinement calculations: full-matrix least- squares on all F^2 values			
Weighting expression w [a]	$[\sigma^2(F_o^2)+(0.0396P)^2 +0.1101P]^{-1}$	$[\sigma^2(F_o^2)+(0.0568P)^2 +0.5054P]^{-1}$	$[\sigma^2(F_o^2)+(0.1053P)^2 + 1.6475P]^{-1}$
No. of refined parameters	118	127	126
No. of restraints	2	0	2
No. of F values used [$I>2\sigma(I)$]	823 [b]	1441	944
Final R-Indices			
$R(=\Sigma\|\Delta F\| / \Sigma\|F_o\|)$	0.0374	0.0377	0.0726
wR on F^2	0.0901	0.0947	0.1712
S (=Goodness of fit on F^2)	1.044	1.055	1.094
Final $\Delta\rho_{max}/\Delta\rho_{min}$ (e Å^{-3})	0.17/-0.17	0.33/-0.28	1.76/-0.88

[a] $P = (F_o^2 + 2F_c^2)/3$; [b] The refinement of twinned structure data with SHELXL-2014/7 [1] does not merge symmetry-equivalent reflexes, precluding a direct determination of R_{Int} or the number of measured/unique reflections. An estimate of R_{Int} is available through the measuring programs X-AREA/LANA [2,3], while the latter data was taken from the fcf-report of the checkcif routine [4].

Table S1: Continued

Crystal structure	**3**-(II)	**4**-(I)	**4**-(II)
Empirical formula	$C_8H_5BrN_2O_2$	$C_8H_5IN_2O_2$	$C_8H_5IN_2O_2$
Formula weight	241.05	288.04	288.04
Crystal system	monoclinic	monoclinic	monoclinic
Space group	*P 2₁/c*	*C 2/c*	*C 2/c*
a (Å)	24.0256(17)	14.0527(5)	10.6756(9)
b (Å)	3.8070(2)	10.1160(2)	10.1072(6)
c (Å)	17.7962(13)	25.0788(9)	16.1947(14)
α(°)	90	90	90
β(°)	95.208(6)	102.934(3)	93.753(7)
γ (°)	90	90	90
V (Å^3)	1621.02(19)	3474.68(19)	1743.7(2)
Z	8	16	8
F(000)	944	2176	1088
D_c (Mg m^{-3})	1.975	2.202	2.194
μ (mm^{-1})	5.034	3.651	3.638
Data collection			
Temperature (K)	160(2)	160(2)	160(2)
No. of collected reflections	19953	14389	8091
within the Θ-limit (°)	2.7 – 27.0	1.7 – 25.5	2.8 – 27.9
Index ranges ±*h*, ±*k*, ±*l*	-30/30, -4/4, -22/22	-14/16, -12/12, -30/30	-14/14, -13/13, -17/21
No. of unique reflections	3514	3240	2080
R_{int}	0.0461	0.0384	0.0208
Refinement calculations: full-matrix least- squares on all F^2 values			
Weighting expression *w* [a]	$[\sigma^2(F_o^2)+(0.0227P)^2 +3.4596P]^{-1}$	$[\sigma^2(F_o^2)+(0.0580P)^2 +5.4500P]^{-1}$	$[\sigma^2(F_o^2)+(0.0189P)^2 +2.5567P]^{-1}$
No. of refined parameters	251	251	126
No. of restraints	0	2	0
No. of F values used [I>2$\sigma(I)$]	2907	2873	1937
Final *R*-Indices			
$R(=\Sigma\|\Delta F\| / \Sigma\|F_o\|)$	0.0301	0.0335	0.0155
wR on F^2	0.0651	0.0845	0.0379
S (=Goodness of fit on F^2)	1.151	1.093	1.083
Final $\Delta\rho_{max}/\Delta\rho_{min}$ (e Å^{-3})	0.70/-0.61	0.50/-1.14	0.39/-0.33

[a] $P = (F_o^2 + 2F_c^2)/3$

Table S1: Continued

Crystal structure	**4**-(III)	**4**-(IV) [b]
Empirical formula	$C_8H_5IN_2O_2$	$C_8H_5IN_2O_2$
Formula weight	288.04	288.04
Crystal system	monoclinic	monoclinic
Space group	*C 2/c*	*C 2/c*
a (Å)	19.9997(7)	47.127(4)
b (Å)	4.4355(1)	4.0991(2)
c (Å)	39.5075(14)	18.6214(14)
α(°)	90	90
β(°)	99.133(3)	105.676(6)
γ (°)	90	90
V (Å^3)	3460.23(19)	3463.4(4)
Z	16	16
F(000)	2176	2176
D_c (Mg m^{-3})	2.212	2.210
μ (mm^{-1})	3.666	3.663
Data collection		
Temperature (K)	160(2)	160(2)
No. of collected reflections	13209	11976
within the Θ-limit (°)	2.1 – 26.0	2.7 – 26.0
Index ranges ±*h*, ±*k*, ±*l*	-24/24, -4/5, -48/48	-53/58, -5/5, -22/22
No. of unique reflections	3406	3369
R_{int}	0.0378	0.1212
Refinement calculations: full-matrix least- squares on all F^2 values		
Weighting expression *w* [a]	$[\sigma^2(F_o^2)+(0.0537P)^2 +6.7024P]^{-1}$	$[\sigma^2(F_o^2)+(0.1275P)^2 +0.0000P]^{-1}$
No. of refined parameters	252	248
No. of restraints	0	4
No. of F values used [$I>2\sigma(I)$]	3146	1899
Final *R*-Indices		
$R(=\Sigma\|\Delta F\| / \Sigma\|F_o\|)$	0.0293	0.0664
wR on F^2	0.0761	0.1683
S (=Goodness of fit on F^2)	1.074	0.949
Final $\Delta\rho_{max}/\Delta\rho_{min}$ (e Å^{-3})	1.03/-1.06	1.70/-1.50

[a] $P = (F_o^2 + 2F_c^2)/3$; [b] the crystals in the sample were small and oftentimes twinned, rendering the collection of an adequate data set difficult.

Table S2: Geometric parameters for non-covalent interactions in the crystal structures examined.

Atoms		Distance (Å)		Angle (°)	Slippage (Å)
D-H···A		D···A	H···A	D-H···A	
C-X···A (X=Br, I; A=O)		C···A	X···A	C-X···A	
π···π		*Cg*···*Cg*			
**1-(I)**					
N(1)-H(1)···N(2)	-*x*, -*y*, -*z*	2.859(4)	2.00(5)	157(5)	
O(2)-H(2)···O(1)	-*x*, -0.5+*y*, -*z*	2.712(3)	1.76(6)	174(5)	
C(5)-H(5)···O(2)	*x*, *y*, *z* (*intra*)	3.063(5)	2.59	111	
Cg(A)···*Cg*(B)[a]	-1+*x*, *y*, *z*	3.411(2)			0.375
Cg(B)···*Cg*(A)[a]	1+*x*, *y*, *z*	3.411(2)			0.493
Cg(B)···*Cg*(B)[a]	1+*x*, *y*, *z*	3.881(2)			1.831
**2-(I)**					
N(1)-H(1)···O(1)	1-*x*, -*y*, 1-*z*	2.840(2)	1.96(3)	167(3)	
O(2)-H(2)···O(1)	1-*x*, 0.5+*y*, 1.5-*z*	2.712(2)	1.86(4)	164(3)	
C(5)-H(5)···O(2)	*x*, *y*, *z* (*intra*)	3.028(2)	2.56	111	
C(7)-H(7)···Cl(1)	-*x*, -0.5+*y*, 0.5-*z*	3.590(2)	2.87	134	
Cg(A)···*Cg*(B)[a]	1-*x*, 1-*y*, 1-*z*	3.613(1)			1.512
Cg(B)···*Cg*(A)[a]	1-*x*, 1-*y*, 1-*z*	3.613(1)			1.510
Cg(B)···*Cg*(B)[a]	-*x*, 1-*y*, 1-*z*	3.718(1)			1.555
**3-(I)**					
N(1)-H(1)···N(2)	0.5+*x*, 0.5-*y*, -0.5+*z*	2.827(11)	2.02(10)	153(10)	
O(2)-H(2)···O(1)	-0.5+*x*, 0.5-*y*, 0.5+*z*	2.772(11)	1.94(14)	172(16)	
C(5)-H(5)···O(2)	*x*, *y*, *z* (*intra*)	3.140(12)	2.68	111	
C(5)-H(5)···Br(1)	1-*x*, 1-*y*, 1-*z*	3.977(11)	3.04	170	
Cg(B)···*Cg*(B)[a]	-1+*x*, *y*, *z*	3.791(7)			1.669
**3-(II)**					
N(1)-H(1)···O(11)	*x*, -0.5-*y*, -0.5+*z*	3.134(3)	2.48(5)	141(4)	
N(1)-H(1)···N(12)	*x*, -0.5-*y*, -0.5+*z*	3.030(4)	2.34(3)	146(5)	
N(11)-H(11)···N(2)	*x*, -1+*y*, *z*	2.873(4)	2.18(4)	142(3)	
N(11)-H(11)···O(1)	*x*, *y*, *z*	3.478(4)	2.74(4)	150(3)	
O(2)-H(2)···O(11)	*x*, *y*, *z*	2.724(3)	1.95(5)	170(5)	
O(12)-H(12)···O(1)	*x*, 0.5-*y*, 0.5+*z*	2.701(3)	1.87(5)	168(5)	
C(5)-H(5)···O(2)	*x*, *y*, *z* (*intra*)	3.152(4)	2.70	110	
C(6)-Br(1)···O(2)	-*x*, -1-*y*, 1-*z*	5.038(3)	3.183(2)	164.1(1)	
C(15)-H(15)···O(12)	*x*, *y*, *z* (*intra*)	3.053(4)	2.58	111	
C(15)-H(15)···Br(11)	1-*x*, 0.5+*y*, 1.5-*z*	3.956(3)	3.02	169	
C(17)-H(17)···Br(11)	1-*x*, 1-*y*, 1-*z*	3.748(3)	3.05	132	
Cg(A)···*Cg*(B)[a]	*x*, 1+*y*, *z*	3.713(2)			1.511
Cg(B)···*Cg*(A)[a]	*x*, -1+*y*, *z*	3.713(2)			1.473
Cg(B)···*Cg*(B)[a]	*x*, 1+*y*, *z*	3.807(2)			1.704
Cg(B')···*Cg*(B')[a]	*x*, -1+*y*, *z*	3.807(2)			1.738

[a] *Cg* means the centroid (centre of gravity) of the ring.
1-(I), **2**-(I), **3**-(I): Ring A: N(1), C(2), C(3), C(4), C(9); ring B: C(4)…C(9).
3-(II): Ring A: N(1), C(2), C(3), C(4), C(9); ring B: C(4)…C(9); ring A': N(11), C(12), C(13), C(14), C(19); ring B': C(14)…C(19).

Table S2: Continued.

Atoms		Distance (Å)		Angle (°)	Slippage (Å)
D-H···A		D···A	H···A	D-H···A	
C-X···A (X=Br, I; A=O)		C···A	X···A	C-X···A	
π···π		*Cg*···*Cg*			
**4-(I)**					
N(1)-H(1)···N(12)	0.5-*x*, 0.5+*y*, 0.5-*z*	2.866(5)	2.23(5)	139(5)	
O(2)-H(2)···O(11)	0.5-*x*, -0.5+*y*, 0.5-*z*	2.705(4)	1.86(5)	171(6)	
N(11)-H(11)···N(2)	0.5-*x*, 0.5+*y*, 0.5-*z*	2.826(4)	2.07(5)	146(5)	
O(12)-H(12)···O(1)	0.5-*x*, -0.5+*y*, 0.5-z	2.771(4)	1.96(5)	162(7)	
C(5)-H(5)···O(2)	*x*, *y*, *z* (*intra*)	3.054(5)	2.58	111	
C(6)-I(1)···I(1)	1.5-*x*, 1.5-*y*, 1-*z*	5.564(4)	3.772(1)	141.1(1)	
C(15)-H(15)···O(12)	*x*, *y*, *z* (*intra*)	3.092(5)	2.63	110	
Cg(A)···*Cg*(B)[a]	1-*x*, *y*, 0.5-*z*	3.499(3)			0.951
Cg(B)···*Cg*(A)[a]	1-*x*, *y*, 0.5-*z*	3.499(3)			0.817
Cg(B)···*Cg*(A')[a]	*x*, *y*, *z*	3.467(3)			0.815
Cg(B)···*Cg*(B')[a]	*x*, *y*, *z*	3.920(2)			1.536
Cg(A')···*Cg*(B)[a]	*x*, *y*, *z*	3.467(3)			0.571
Cg(B)···*Cg*(B')[a]	0.5-*x*, 1.5-*y*, 1-*z*	3.909(2)			1.533
**4-(II)**					
N(1)-H(1)···N(2)	0.5-*x*, 0.5+*y*, 0.5-*z*	2.827(2)	2.10(3)	147(3)	
O(2)-H(2)···O(1)	0.5-*x*, -0.5+*y*, 0.5-*z*	2.757(2)	2.03(3)	169(3)	
C(5)-H(5)···O(2)	*x*, *y*, *z* (*intra*)	3.081(2)	2.61	111	
C(6)-I(1)···I(1)	2-*x*, 1-*y*, 1-*z*	2.825(5)	3.898(1)	136.8(1)	
Cg(A)···*Cg*(B)[a]	1-*x*, *y*, 0.5-*z*	3.549(1)			1.225
Cg(B)···*Cg*(A)[a]	1-*x*, *y*, 0.5-*z*	3.549(1)			1.152
Cg(B)···*Cg*(B)[a]	1-*x*, 1-*y*, 1-*z*	3.871(1)			1.422
**4-(III)**					
N(1)-H(1)···N(12)	0.5+*x*, -0.5+*y*, *z*	2.901(4)	2.14(5)	142(5)	
O(2)-H(2)···O(11)	*x*, *y*, *z*	2.704(4)	2.01(6)	163(5)	
N(11)-H(11)···N(2)	*x*, *y*, *z*	2.833(4)	2.09(3)	146(5)	
O(12)-H(12)···O(1)	-0.5+*x*, 0.5+*y*, *z*	2.694(4)	1.85(6)	163(5)	
C(5)-H(5)···O(2)	*x*, *y*, *z* (*intra*)	3.031(4)	2.55	111	
C(6)-I(1)···O(2)	1-*x*, 1+*y*, 0.5-*z*	5.564(4)	3.383(2)	169.4(1)	
C(15)-H(15)···O(12)	*x*, *y*, *z* (*intra*)	3.070(4)	2.60	111	
Cg(A)···*Cg*(B)[a]	*x*, -1+*y*, *z*	3.630(2)			1.468
Cg(B)···*Cg*(A)[a]	*x*, 1+*y*, *z*	3.630(2)			1.380
Cg(A')···*Cg*(B')[a]	*x*, 1+*y*, *z*	3.520(2)			0.854
Cg(B')···*Cg*(A')[a]	*x*, -1+*y*, *z*	3.520(2)			0.718

[a] *Cg* means the centroid (centre of gravity) of the ring.
4-(II): Ring A: N(1), C(2), C(3), C(4), C(9); ring B: C(4)…C(9).
4-(I), **4**-(III): Ring A: N(1), C(2), C(3), C(4), C(9); ring B: C(4)…C(9); ring A': N(11), C(12), C(13), C(14), C(19); ring B': C(14)…C(19).

Table S2: Continued.

Atoms		Distance (Å)		Angle (°)	Slippage (Å)
D-H···A		D···A	H···A	D-H···A	
C-X···A (X=Br, I; A=O)		C···A	X···A	C-X···A	
π···π		*Cg*···*Cg*			
**4-(IV)**					
N(1)-H(1)···N(12)	*x*, 1-*y*,-0.5+*z*	3.081(17)	2.55(14)	120(12)	
N(1)-H(1)···O(11)	*x*, 1-*y*,-0.5+*z*	3.205(14)	2.41(5)	151(11)	
N(11)-H(11)···N(2)	*x*, *y*, *z*	2.859(19)	2.03(14)	158(14)	
O(2)-H(2)···O(11)	*x*, *y*, *z*	2.758(13)	2.01(18)	148(17)	
O(12)-H(12)···O(1)	*x*, -*y*, 0.5+*z*	2.665(13)	1.84(8)	167(16)	
C(5)-H(5)···O(2)	*x*, *y*, *z* (*intra*)	3.175(16)	2.74	109	
C(6)-I(1)···O(2)	1-*x*, 1+*y*, 1.5-*z*	5.390(14)	3.287(9)	173.4(4)	
C(15)-H(15)···O(12)	*x*, *y*, *z* (*intra*)	3.053(16)	2.58	111	
C(16)-I(11)···O(12)	0.5-*x*, -0.5+*y*, 1.5-*z*	5.353(14)	3.285(9)	168.8(4)	
Cg(A)···*Cg*(B)[a]	*x*, -1+*y*, *z*	3.575(8)			0.803
Cg(B)···*Cg*(A)[a]	*x*, 1+*y*, *z*	3.576(8)			0.851
Cg(A‘)···*Cg*(B’)[a]	*x*, 1+*y*, *z*	3.811(8)			1.623
Cg(B‘)···*Cg*(A’)[a]	*x*, -1+*y*, *z*	3.811(8)			1.570

[a] *Cg* means the centroid (centre of gravity) of the ring.
4-(IV): Ring A: N(1), C(2), C(3), C(4), C(9); ring B: C(4)…C(9); ring A’: N(11), C(12), C(13), C(14), C(19); ring B’: C(14)…C(19).

Table S3: Solvents used for crystallization experiments

Solvents tested for crystallization experiments	
n-Hexane	1,4-Dioxane
n-Hexane/Acetone	Acetone
n-Heptane	Ethyl acetate
n-Heptane/Ethyl acetate	Dimethyl carbonate
Toluene	*N*,*N*-Dimethylformamide
Pyridine	Dimethylsulfoxide
Dichloromethane	Acetonitrile
Chloroform	*n*-Butanol
Diethyl ether	*iso*-Propanol
Diisopropyl ether	Ethanol
1,2-Dimethoxyethane	Methanol
2,2-Dimethoxypropane	Water
Tetrahydrofuran	Water/Tetrahydrofuran

Table S4: Results of crystallization experiments depending on the employed solvent(s).

solvent	1*H*-Isatin-3-oxime	5-Chloroisatin-3-oxime	5-Bromoisatin-3-oxime	5-Iodoisatin-3-oxime
n-Hexane/Acetone	-	**2**-(I)	-	**4**-(I)
n-Heptane/Ethyl acetate	-	-	**3**-(II)	**4**-(I)
Pyridine	-	**2**-(I)	-	-
1,2-Dimethoxyethane	**1**-(I)	**2**-(I)	**3**-(I)/**3**-(II)	-
Tetrahydrofuran	-	**2**-(I)	-	**4**-(I)/**4**-(IV) [a]
1,4-Dioxane	**1**-(I)	**2**-(I)	-	**4**-(I)
Acetone	-	**2**-(I)	-	**4**-(I)
Ethyl acetate	-	-	-	**4**-(I)
N,*N*-Dimethylformamide	-	**2**-(I)	**3**-(II)	-
Dimethylsulfoxide	**1**-(I)	**2**-(I)	**3**-(II)	**4**-(I)
Acetonitrile	-	-	-	**4**-(I)
n-Butanol	-	**2**-(I)	-	-
iso-Propanol	-	**2**-(I)	-	**4**-(I)
Ethanol	**1**-(I)	**2**-(I)	-	**4**-(I)/**4**-(II)
Methanol	-	**2**-(I)	**3**-(II)	**4**-(I)/**4**-(II)
Water	**1**-(I)	X	X	X[c]
Water/Tetrahydrofuran	-	**2**-(I)	-	**4**-(I)/**4**-(III) [b]

[a] **4**-(IV) could only be obtained a single time. All following experiments in THF yielded **4**-(I). [b] Recrystallization of the iodinated compound from H_2O/THF resulted in **4**-(I), while slowly evaporating a respective solution yielded **4**-(III).[c] "X" marks instances where the solubility of the compound was too low for an adequate experiment.

Search in the Cambridge Structural Database (CSD): NOH_{oxime}···X hydrogen bonds (X = F, Cl, Br, I) (Figure S1)

In order to determine the prevalence and nature of hydrogen bonding between oxime and halogen substituents in the Cambridge Structural Database (CSD), the interaction was modeled as follows: $(NM)_2C$=NOH···X-NM, where NM is any non-metal, X is any halogen, and the hydrogen bond geometry was restrained to distances shorter than the sum of the van der Waals radii as well as angles of ∠(O-H···X) greater than 110°.

The search revealed that in the overwhelming majority of cases, neutral halogen atoms do not function as primary acceptors of oxime-based hydrogen bonds. Instead, the NOH···X contacts can be interpreted as secondary interactions resulting from bifurcated hydrogen bonds to more potent primary acceptors. On one hand, the latter can be the oxime groups themselves, leading to the dimeric arrangements represented in Figure S1a. On the other hand, various different lewis-basic centers (denoted Y in Figure S1b) can fill the role of the primary hydrogen bond acceptor, predominantly giving rise to supramolecular dimers or 0-dimensional structures. An additional observation pertains to the identity of the halogen, which is oftentimes occupied by an F atom, while NOH···Cl/Br contacts occur significantly less often and I atoms are virtually never involved.

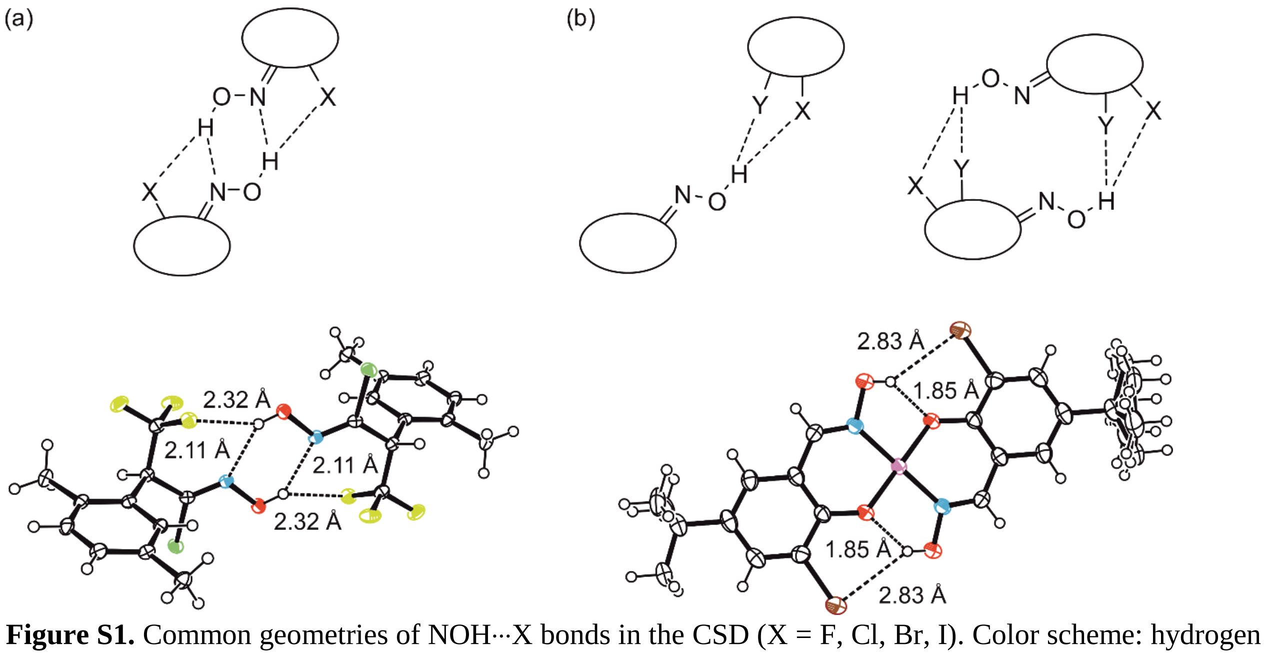

Figure S1. Common geometries of NOH···X bonds in the CSD (X = F, Cl, Br, I). Color scheme: hydrogen = white (circles) carbon = white (ellipsoids), nitrogen = blue, oxygen = red, fluorine = yellow, chlorine = green, bromine = brown, nickel = pink. (a) Dimeric arrangement centered around an $R_2^2(6)$ oxime motif with secondary NOH···X contacts. An example can be found in the structure KIGHIU [5]. (b) 0-dimensional or dimeric structures resulting from bifurcated hydrogen bonds from the oxime moiety to another primary acceptor (Y) and the halogen (X). As an example, an excerpt of the structure DUJHIB [6] is shown.

Comparison of the packing behaviors of polymorphs 4-(III) and 4-(IV) (Figure S2)

(a)

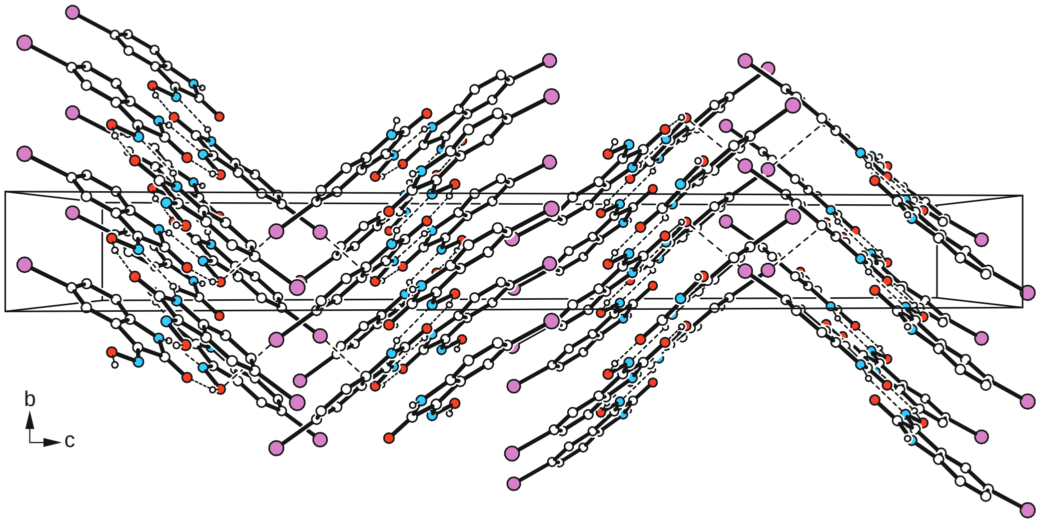

(b)

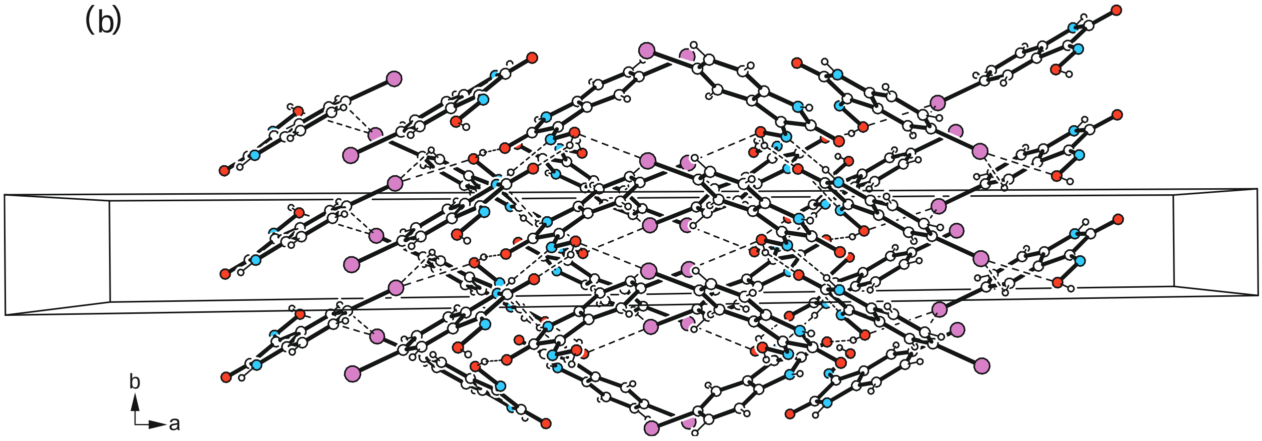

Figure S2. Packing diagrams of (a) the polymorph **4**-(III) viewed in direction of the *a*-axis and (b) of polymorph **4**-(IV) viewed along the c-axis.

2D fingerprint plots for overall and individual interactions in the crystal packing of compounds 1-4 (Figures S3-S14)

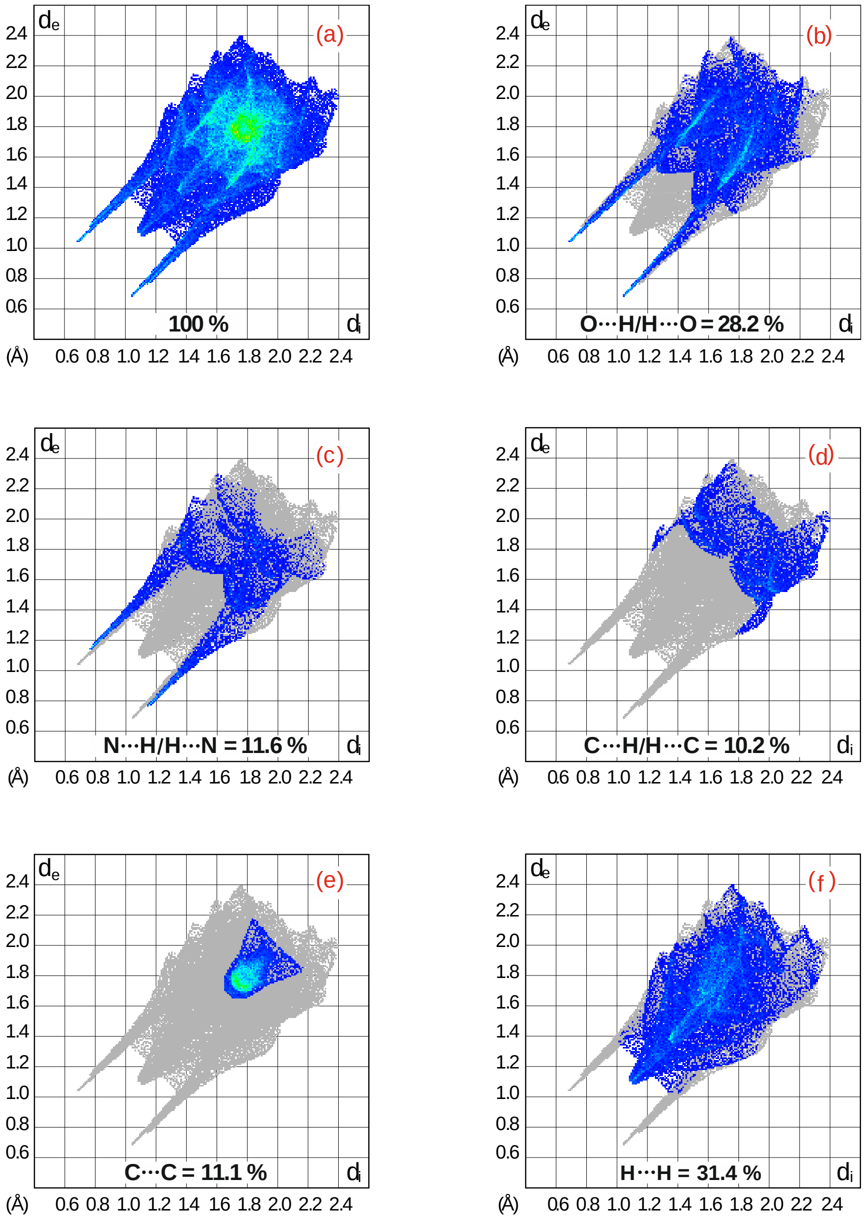

Figure S3. 2D fingerprint plots for (a) overall interactions and (b-f) individual interactions in the crystal packing of **1**-(I).

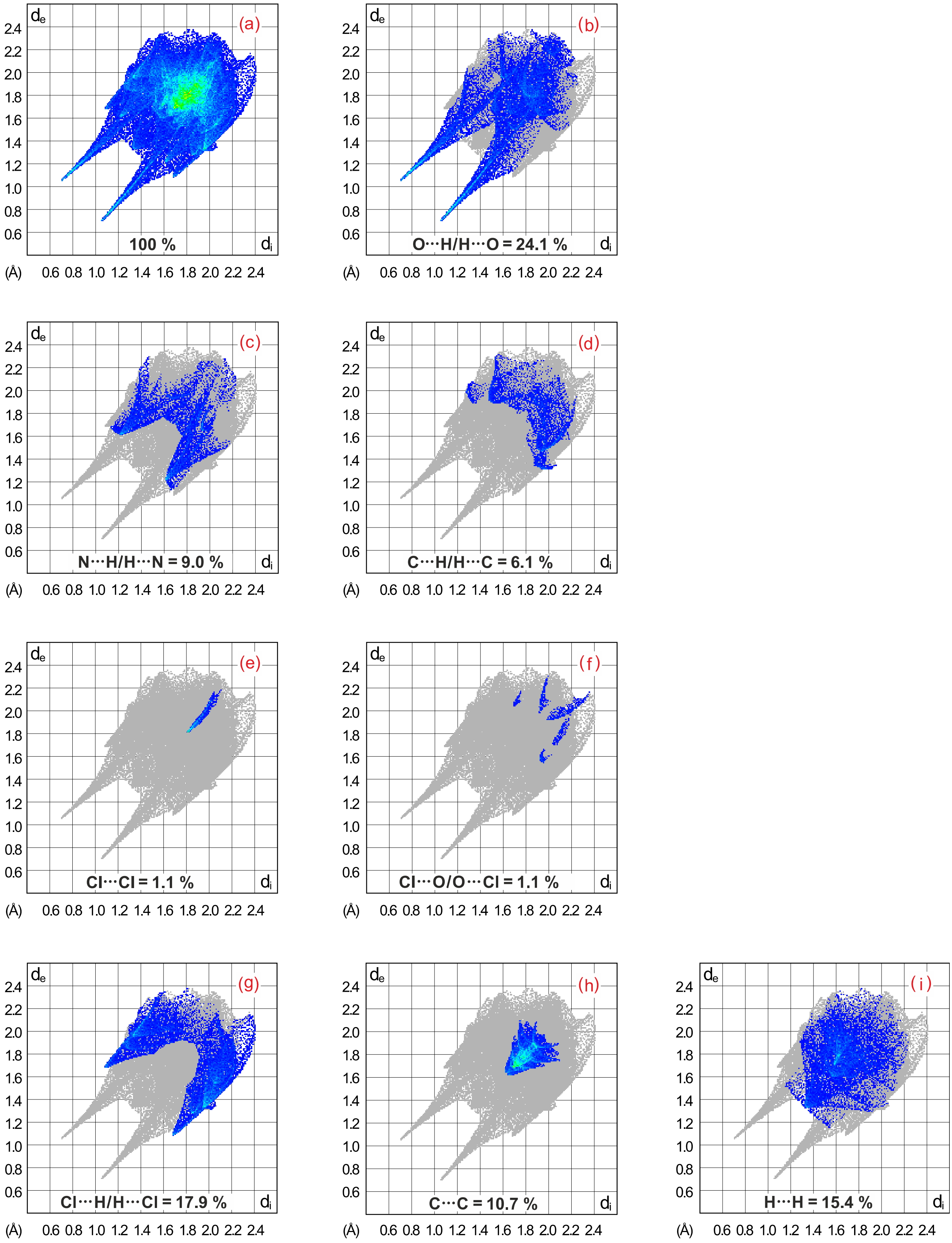

Figure S4. 2D fingerprint plots for (a) overall interactions and (b-i) individual interactions in the crystal packing of **2**-(I).

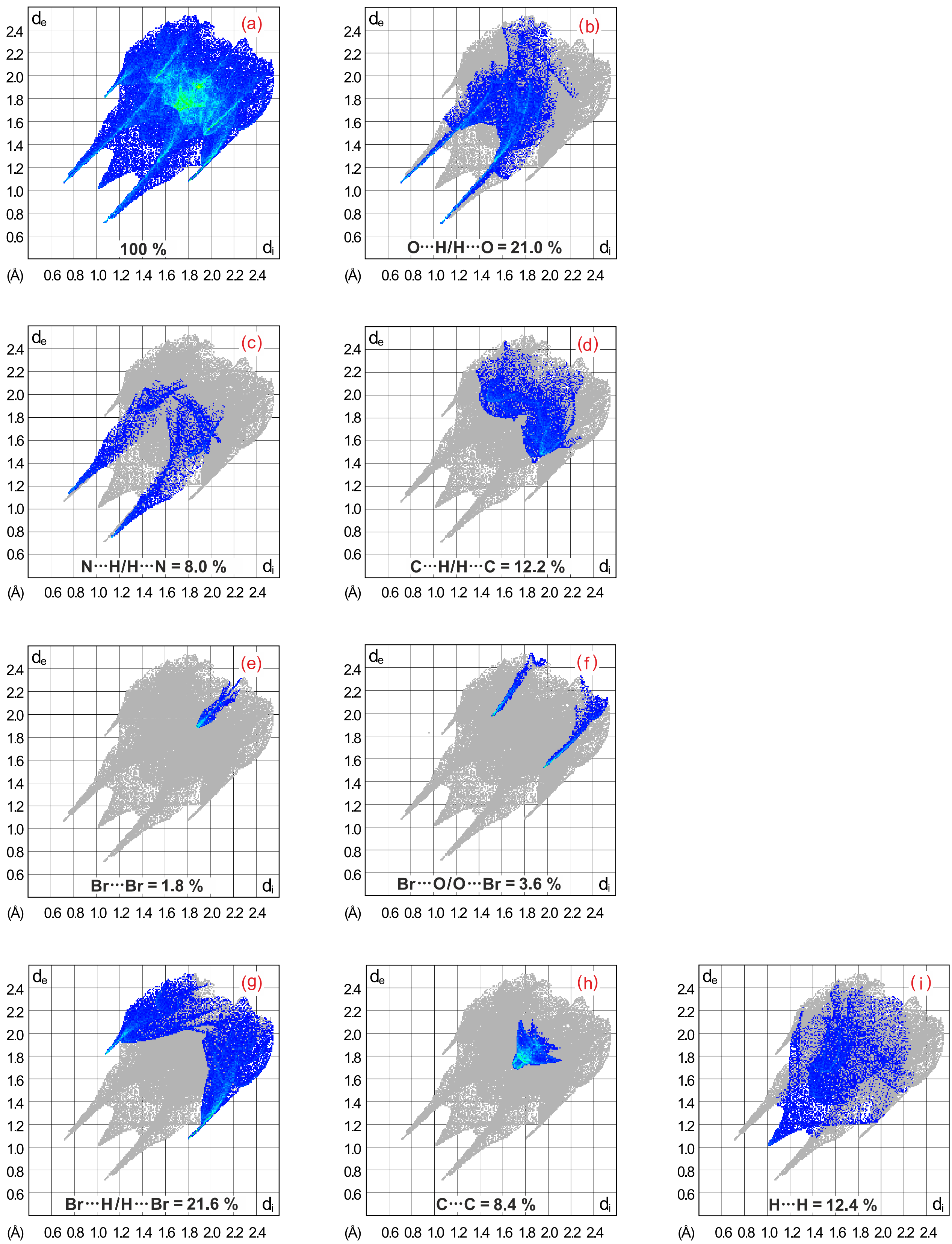

Figure S5. 2D fingerprint plots for (a) overall interactions and (b-i) individual interactions in the crystal packing of **3**-(I).

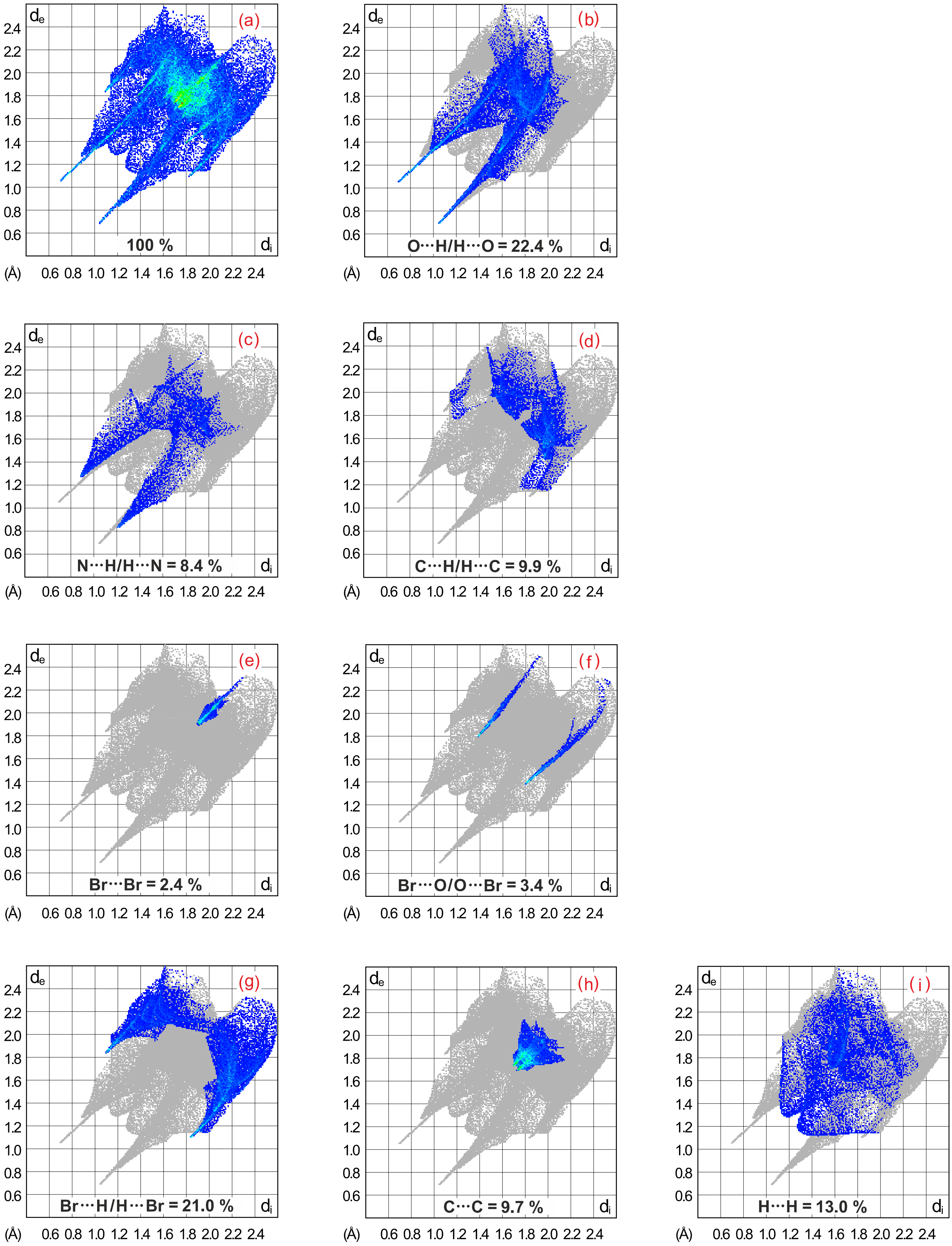

Figure S6. 2D fingerprint plots for (a) overall interactions and (b-i) individual interactions in the crystal packing of **3**-(II) (molecule A).

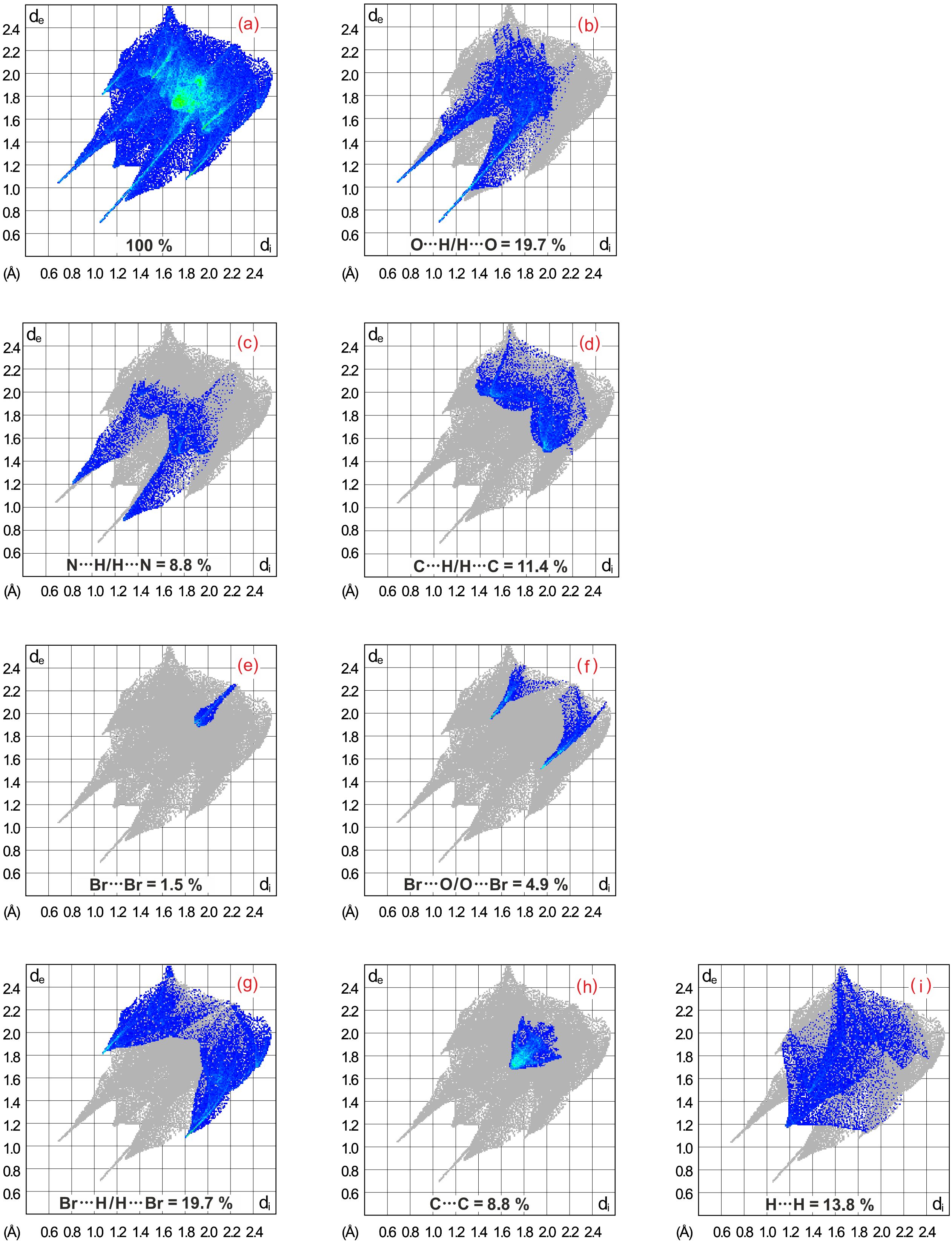

Figure S7. 2D fingerprint plots for (a) overall interactions and (b-i) individual interactions in the crystal packing of **3**-(II) (molecule B).

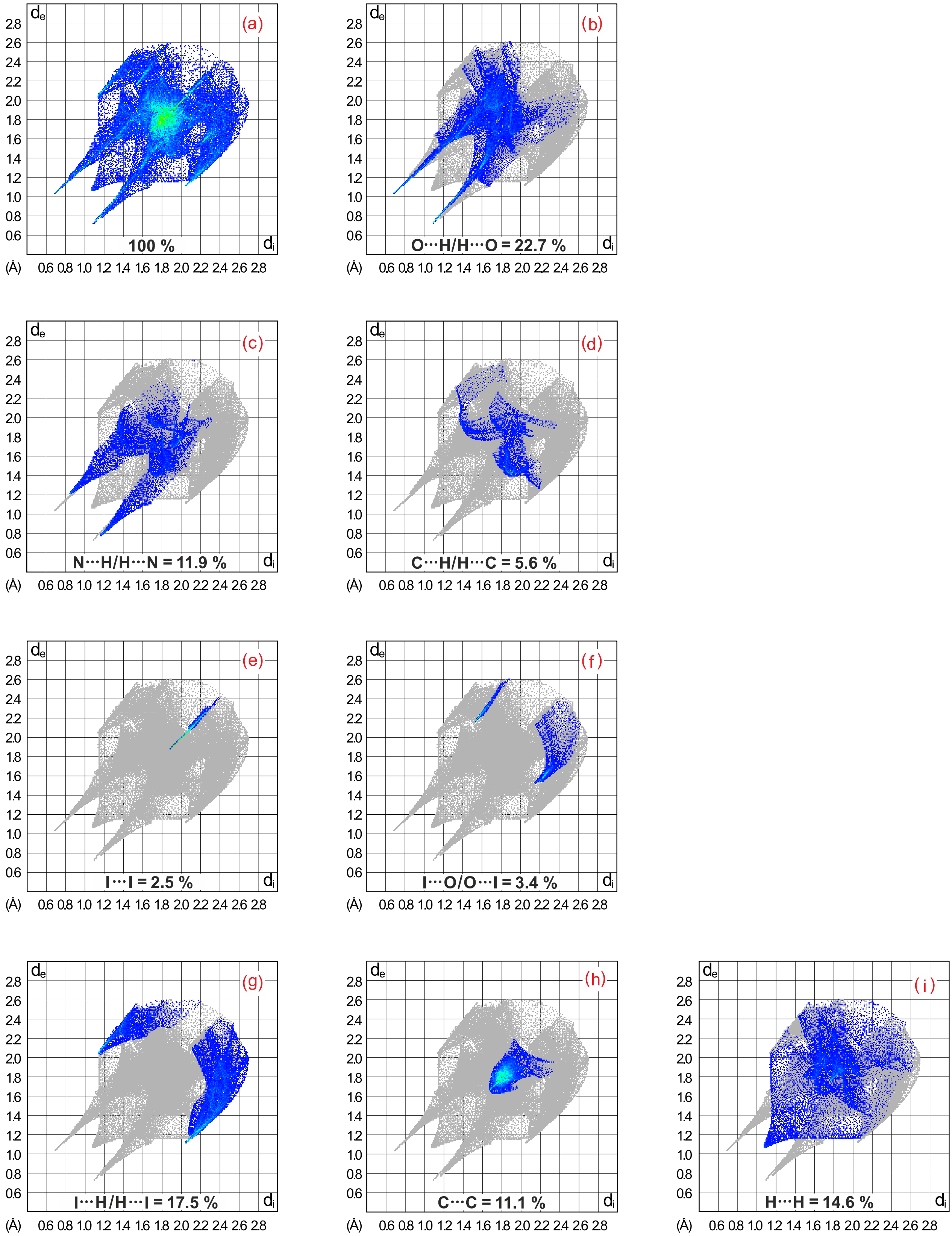

Figure S8. 2D fingerprint plots for (a) overall interactions and (b-i) individual interactions in the crystal packing of **4**-(I) (molecule A).

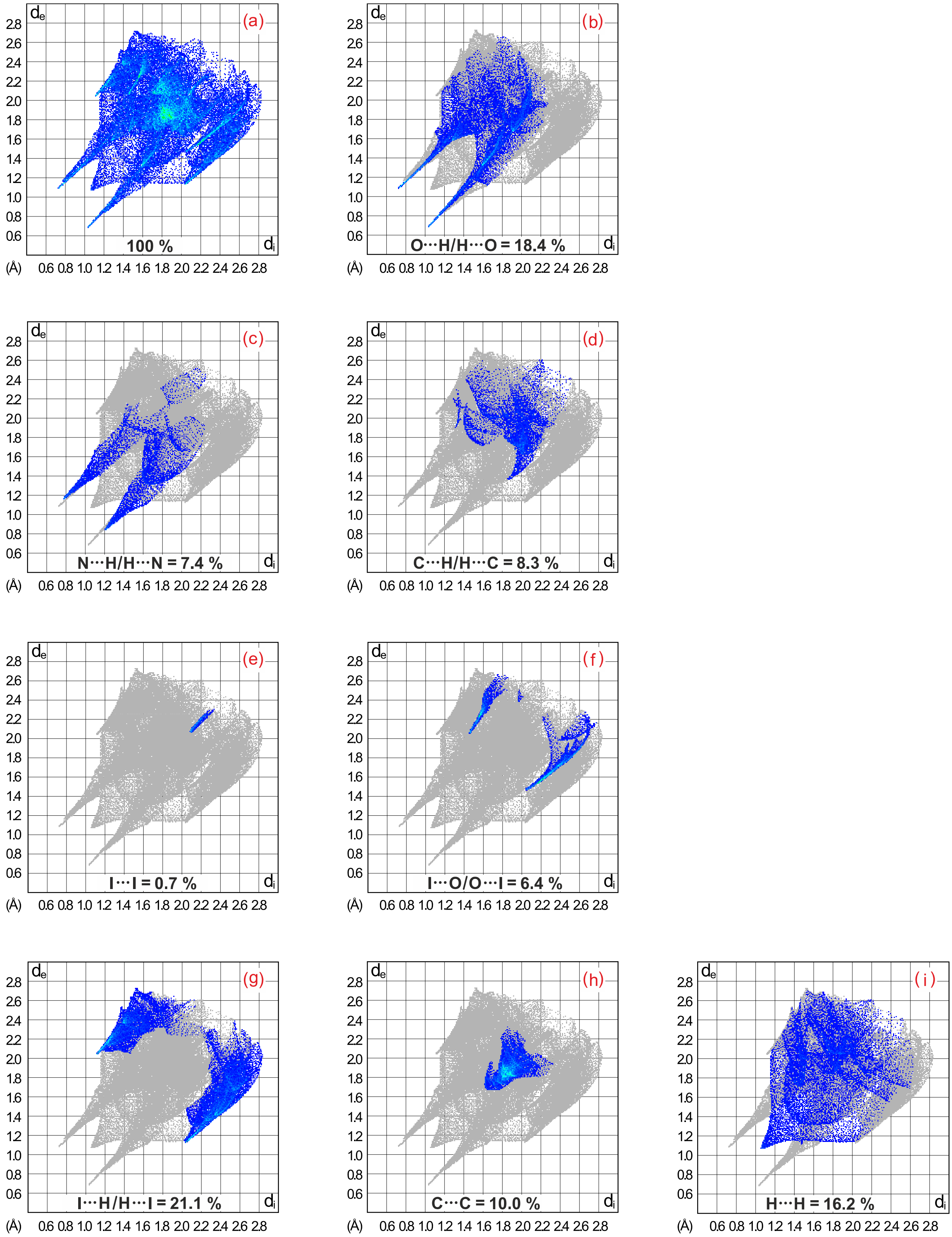

Figure S9. 2D fingerprint plots for (a) overall interactions and (b-i) individual interactions in the crystal packing of **4**-(I) (molecule B).

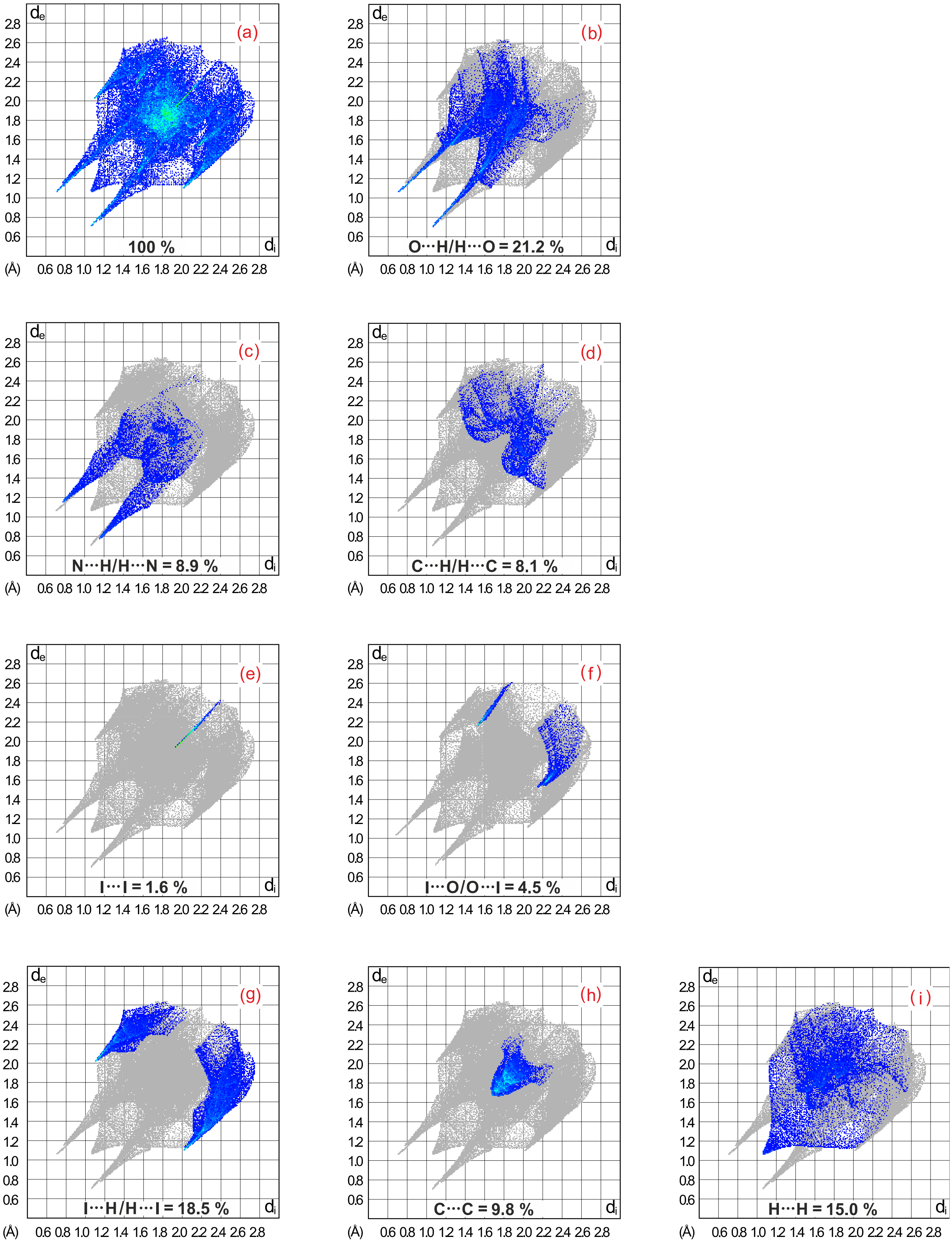

Figure S10. 2D fingerprint plots for (a) overall interactions and (b-i) individual interactions in the crystal packing of **4**-(II).

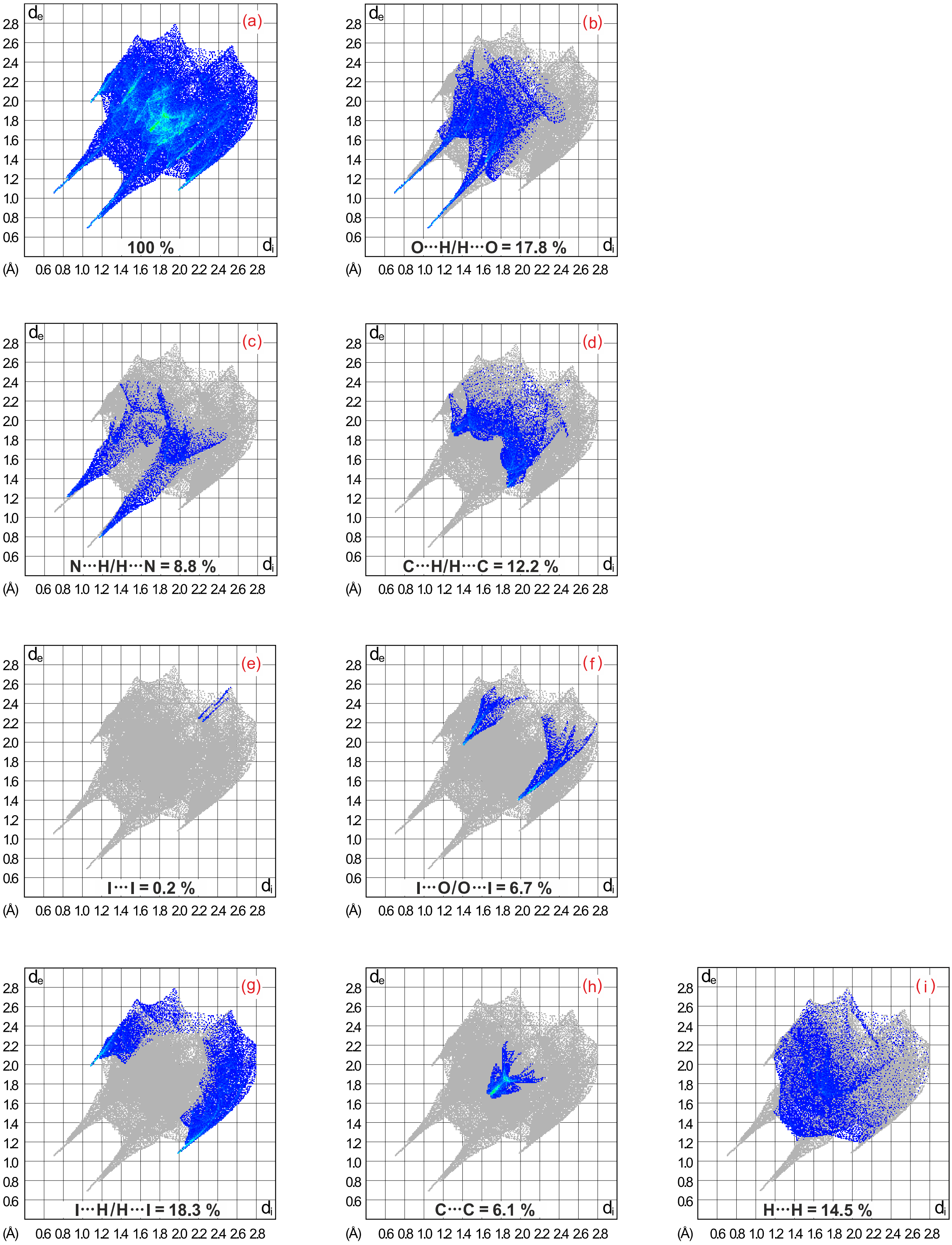

Figure S11. 2D fingerprint plots for (a) overall interactions and (b-i) individual interactions in the crystal packing of **4**-(III) (molecule A).

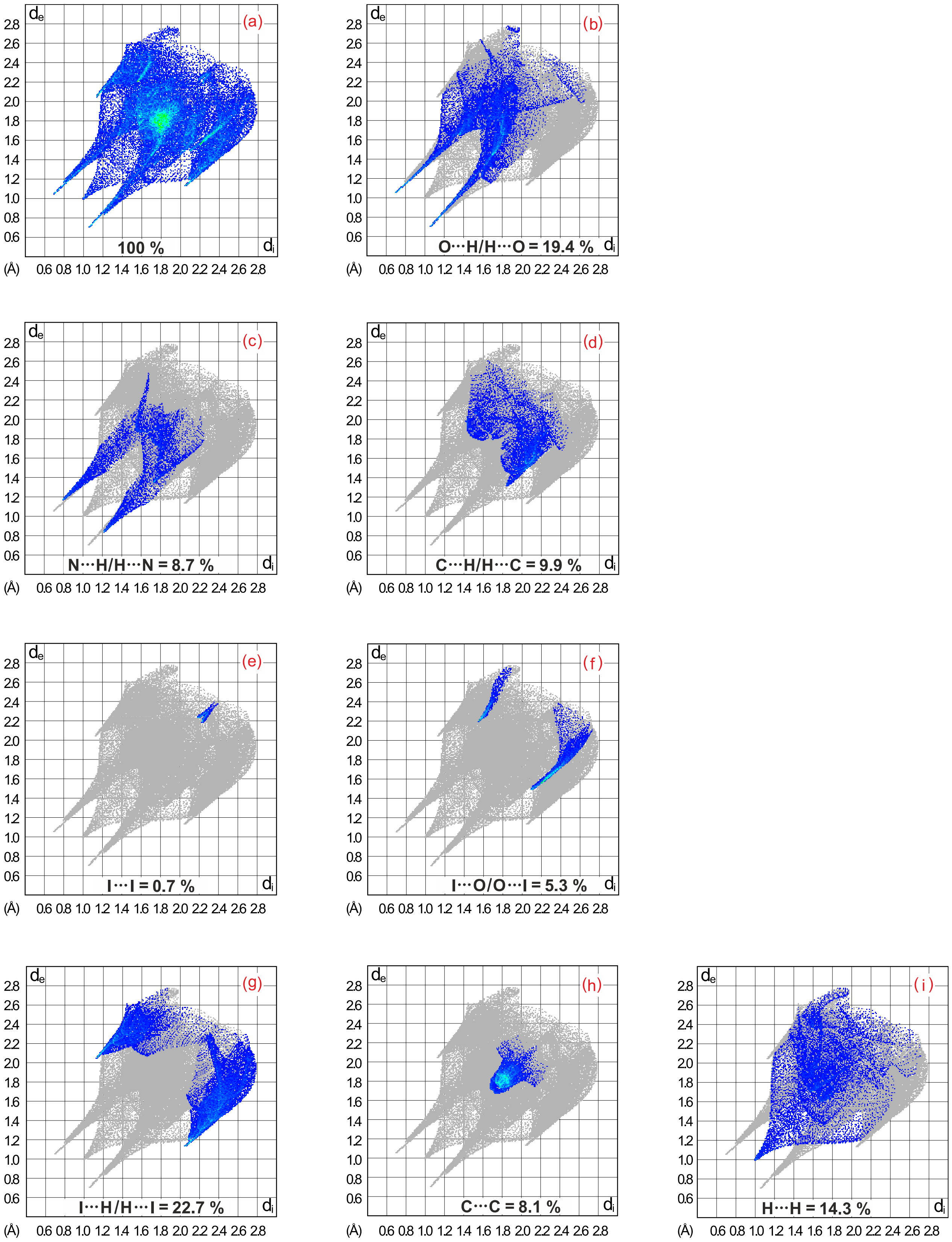

Figure S12. 2D fingerprint plots for (a) overall interactions and (b-i) individual interactions in the crystal packing of **4**-(III) (molecule B).

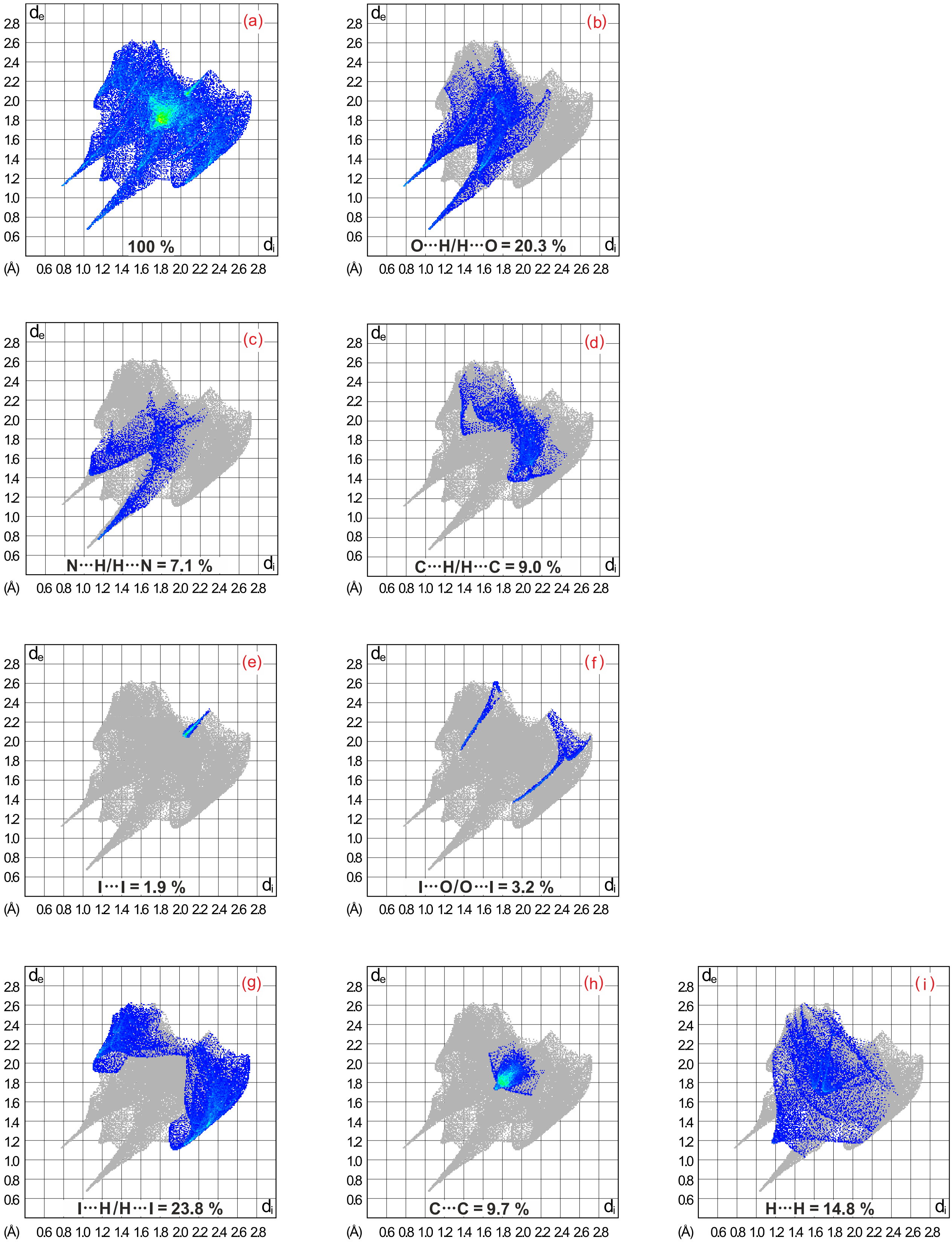

Figure S13. 2D fingerprint plots for (a) overall interactions and (b-i) individual interactions in the crystal packing of **4**-(IV) (molecule A).

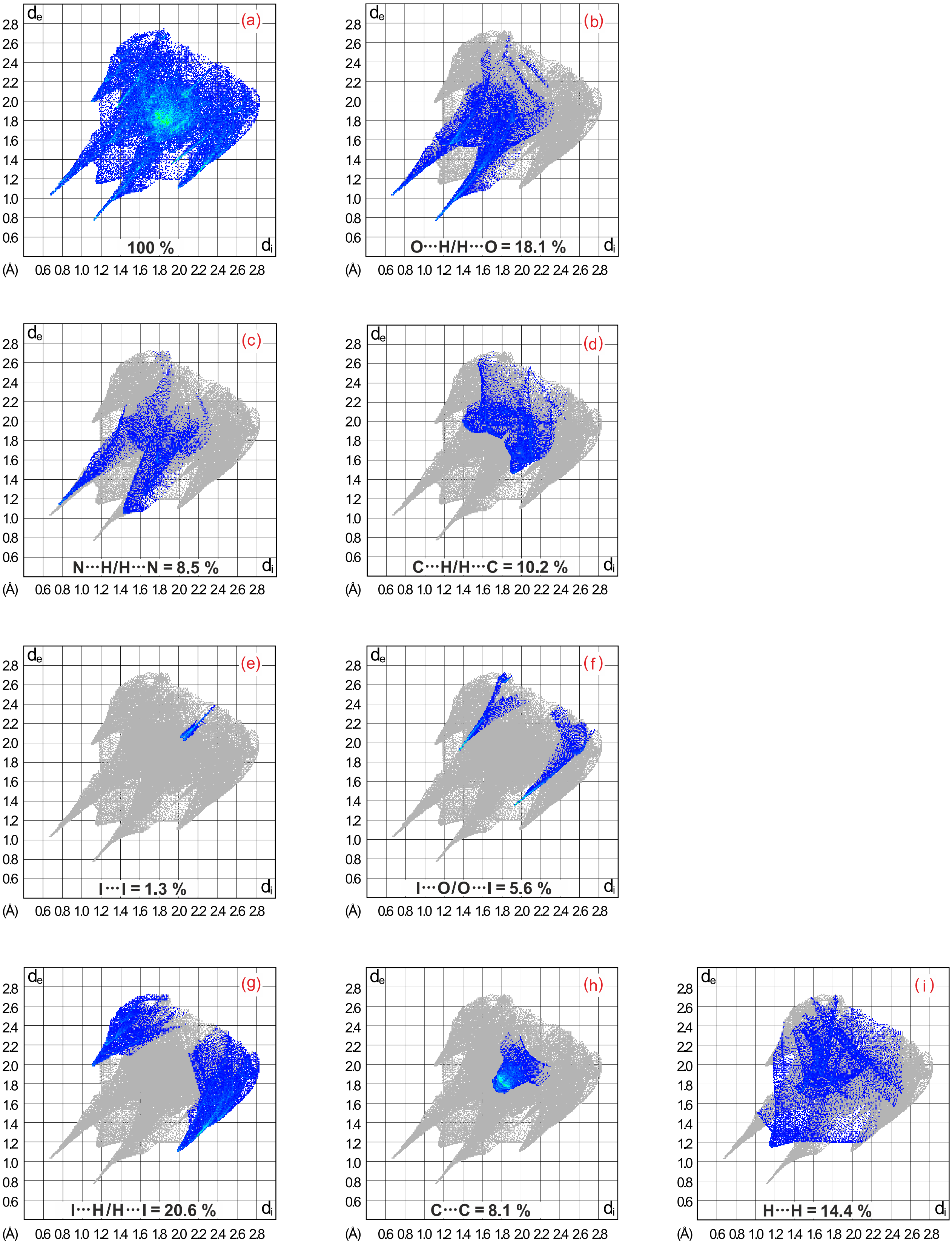

Figure S14. 2D fingerprint plots for (a) overall interactions and (b-i) individual interactions in the crystal packing of **4**-(IV) (molecule B).

Hirshfeld surface plots of the crystal phases of compounds 3 and 4 (Figures S15-S20)

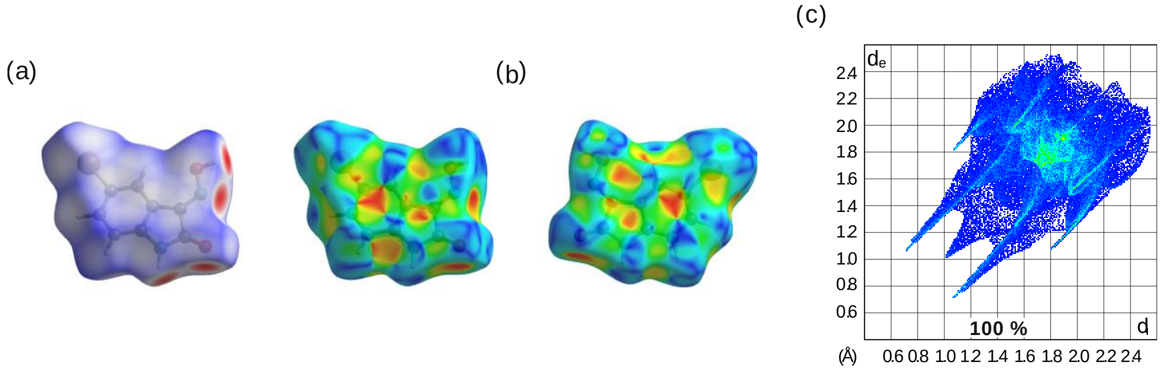

Figure S15. 3D Hirshfeld surface plot of **3**-(I) mapped over d_{norm} (a), HS mapped over the shape index (b) and 2D fingerprint for overall interactions for **3**-(I) (c).

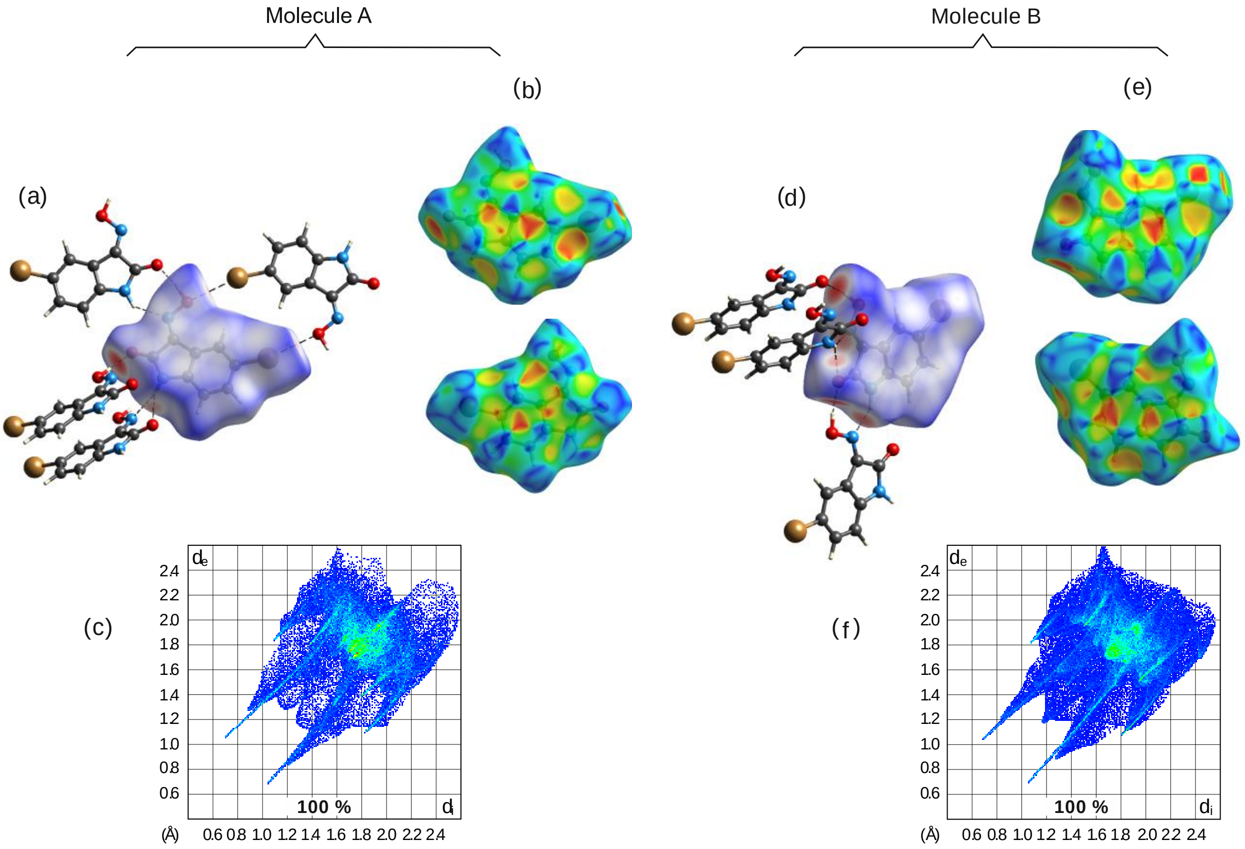

Figure S16. d_{norm}, selected intermolecular contacts, shape index (front and back side) and 2D fingerprint for overall interactions for molecule A (a-c) and molecule B (d-f) of **3**-(II).

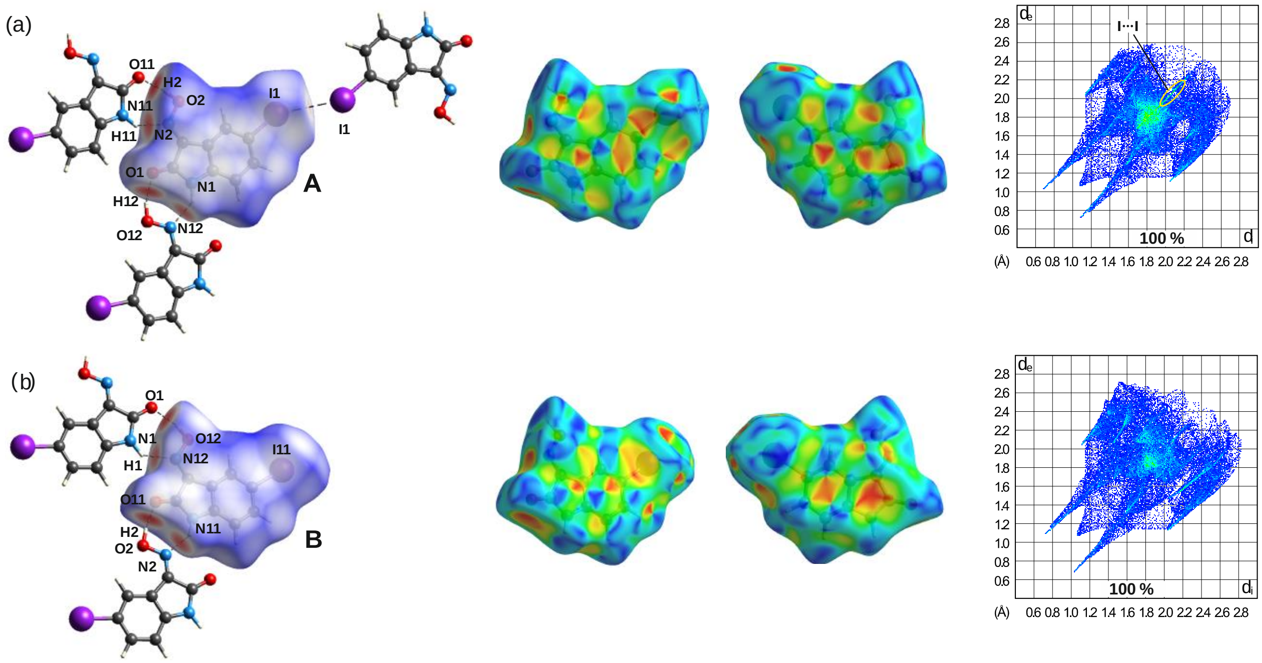

Figure S17. d_{norm}, selected intermolecular contacts, shape index (front and back side) and 2D fingerprint for overall interactions for molecule A (a-c) and molecule B (d-f) of **4**-(I).

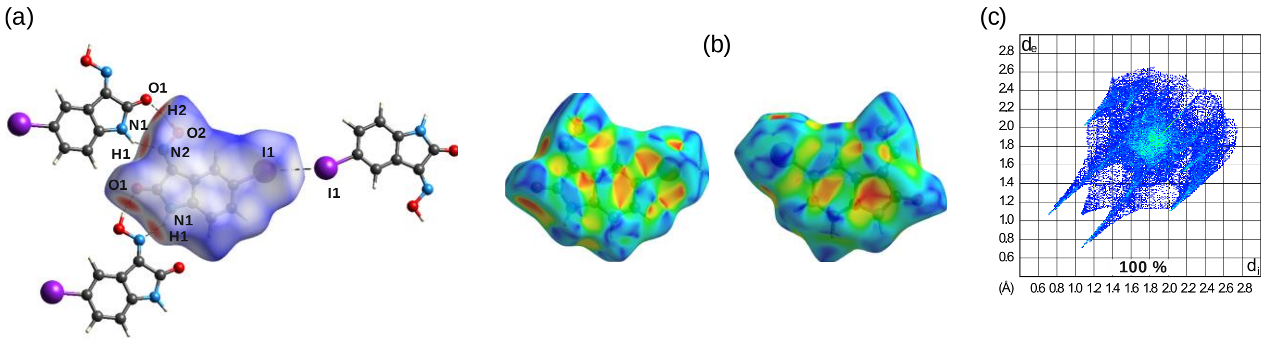

Figure S18. (a) d_{norm}, selected intermolecular contacts, (b) shape index (front and back side) and (c) 2D fingerprint for overall interactions of **4**-(II).

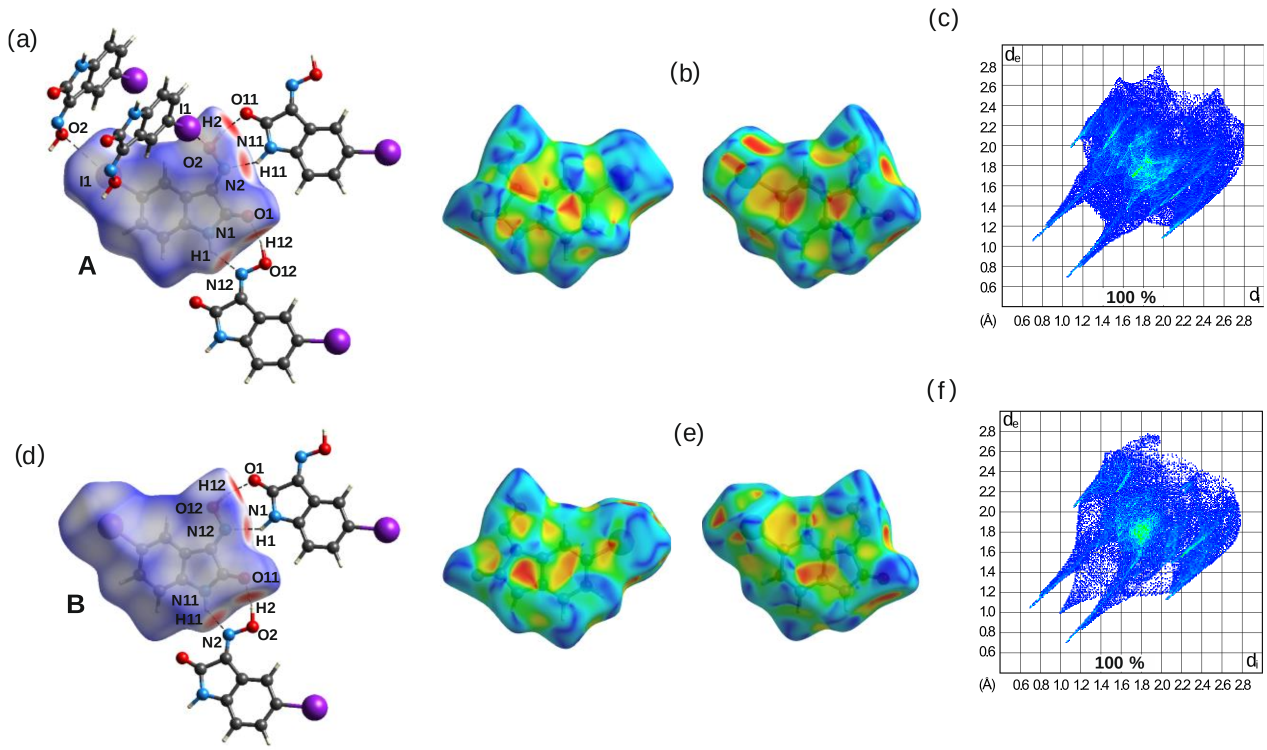

Figure S19. d_{norm}, selected intermolecular contacts, shape index (front and back side) and 2D fingerprint for overall interactions for molecule A (a-c) and molecule B (d-f) of **4**-(III).

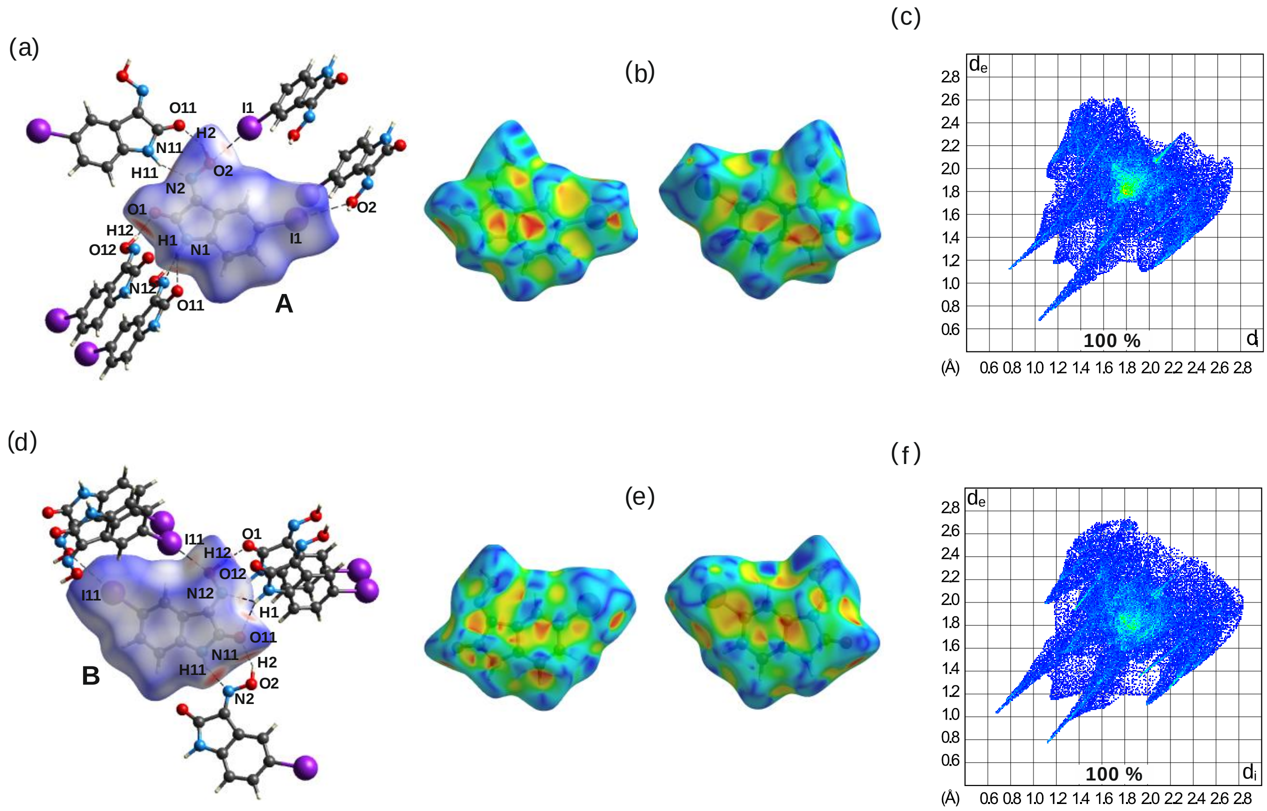

Figure S20. d_{norm}, selected intermolecular contacts, shape index (front and back side) and 2D fingerprint for overall interactions for molecule A (a-c) and molecule B (d-f) of **4**-(IV).

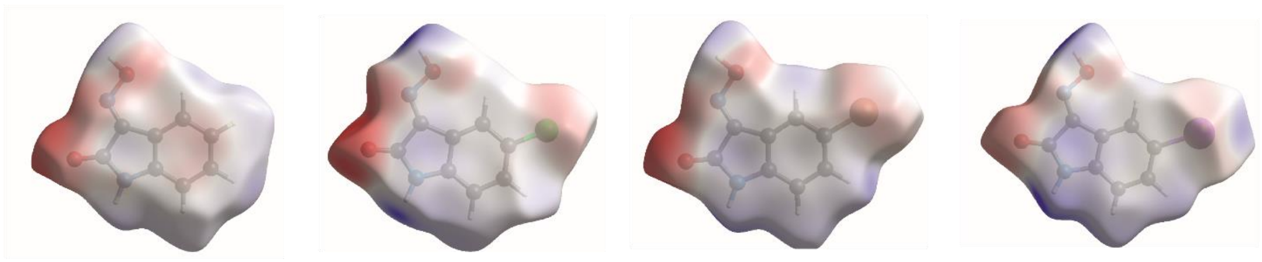

1-(I) **2**-(I) **3**-(I) **4**-(II)

Figure S21. Calculated electrostatic potential for compounds **1** – **4** mapped on the respective Hirshfeld surfaces (blue = positive, red = negative).

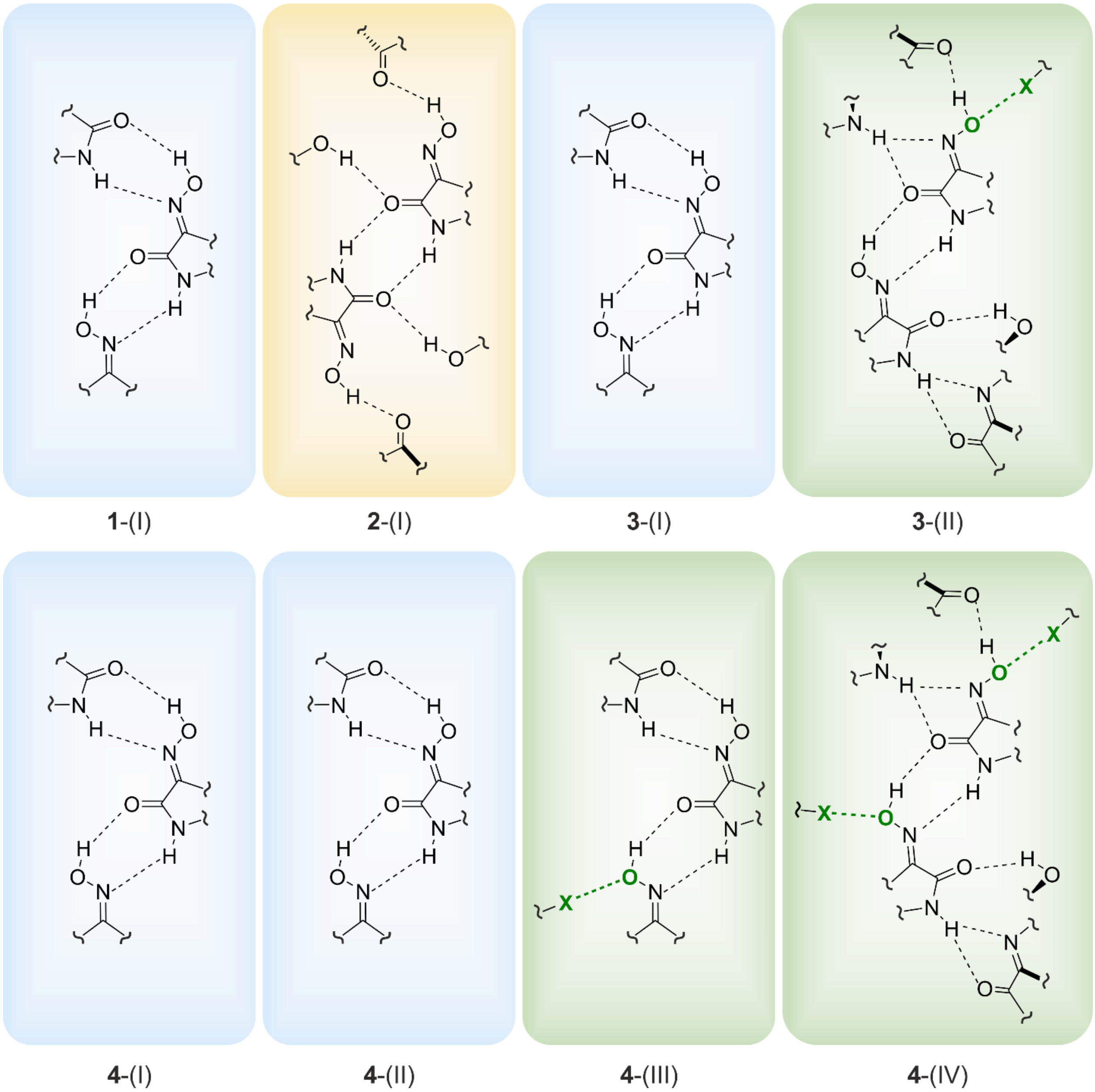

Figure S22. Overview of hydrogen and halogen bonding patterns in the crystal structures **1**-(I), **2**-(I), **3**-(I), **3**-(II) and **4**-(I) – **4**-(IV). The coloring highlights the main supramolecular motifs: $R_2^2(7)$ hydrogen bonding pattern (blue), $R_2^2(8)$ hydrogen bonding pattern (yellow) or $R_2^2(7)$ hydrogen bonding pattern in combination with X···O_{oxime} halogen bonds (green).

^{1}H- and ^{13}C-NMR spectra of compounds 1-4 (Figures S23-S30)

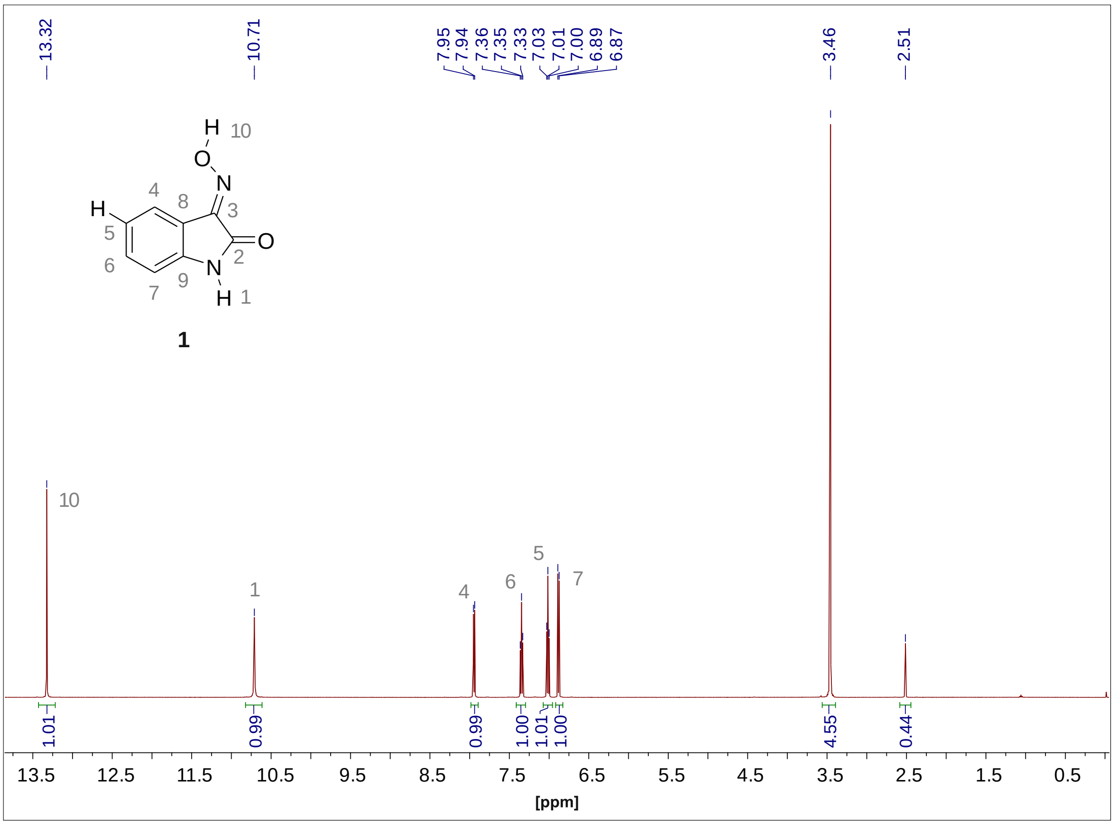

Figure S23. ^{1}H-NMR spectrum (500 MHz) of compound **1** in DMSO-d_6.

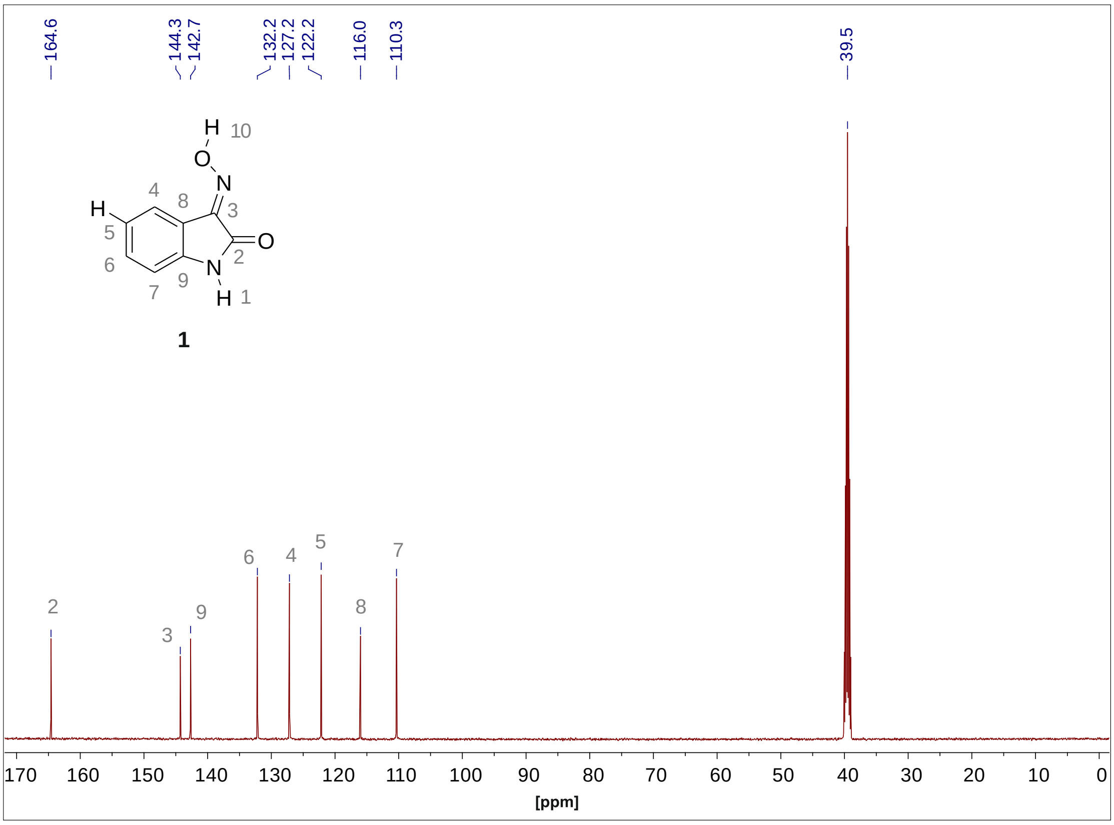

Figure S24. ^{13}C-NMR spectrum (125 MHz) of compound **1** in DMSO-d_6.

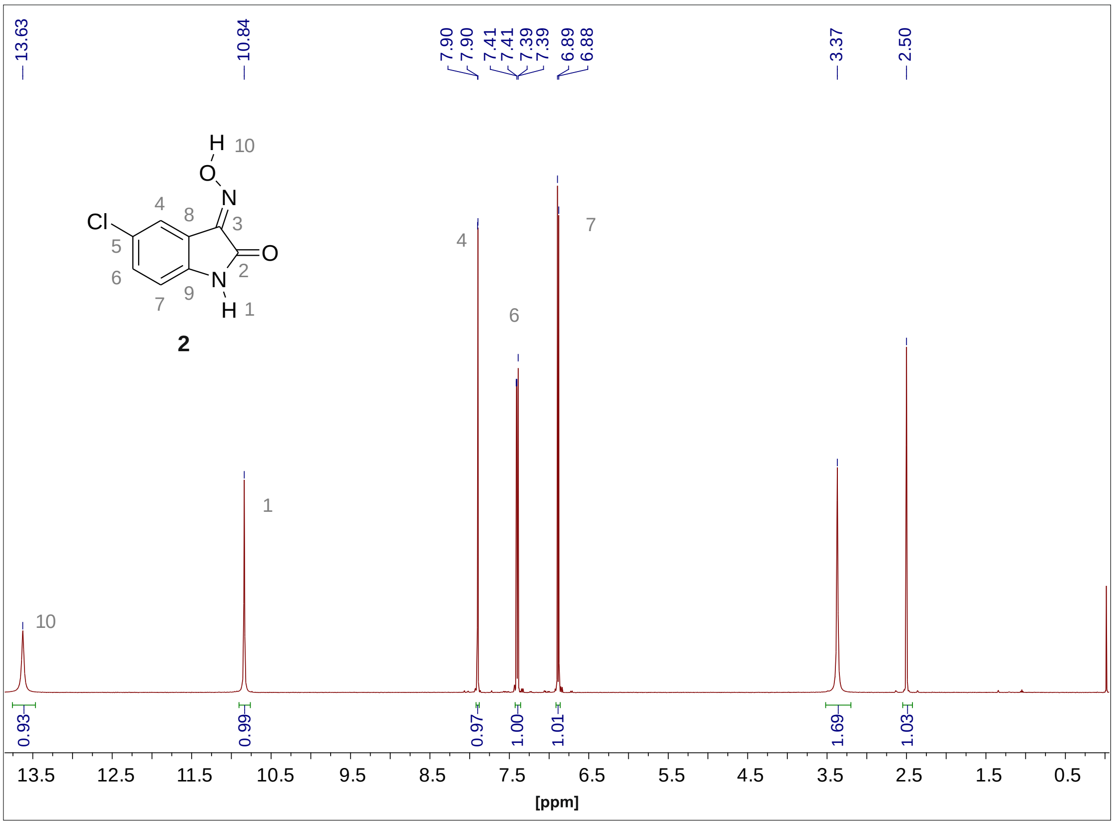

Figure S25. ^{1}H-NMR spectrum (500 MHz) of compound **2** in DMSO-d_6.

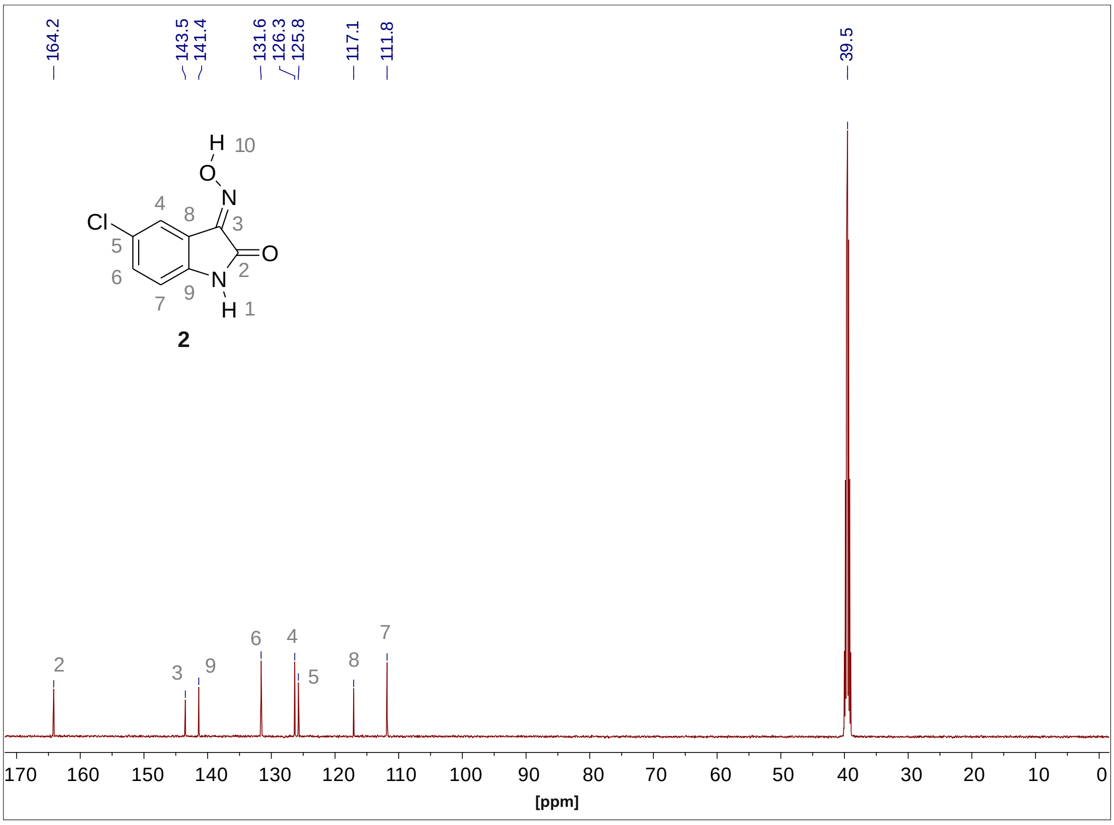

Figure S26. ^{13}C-NMR spectrum (125 MHz) of compound **2** in DMSO-d_6.

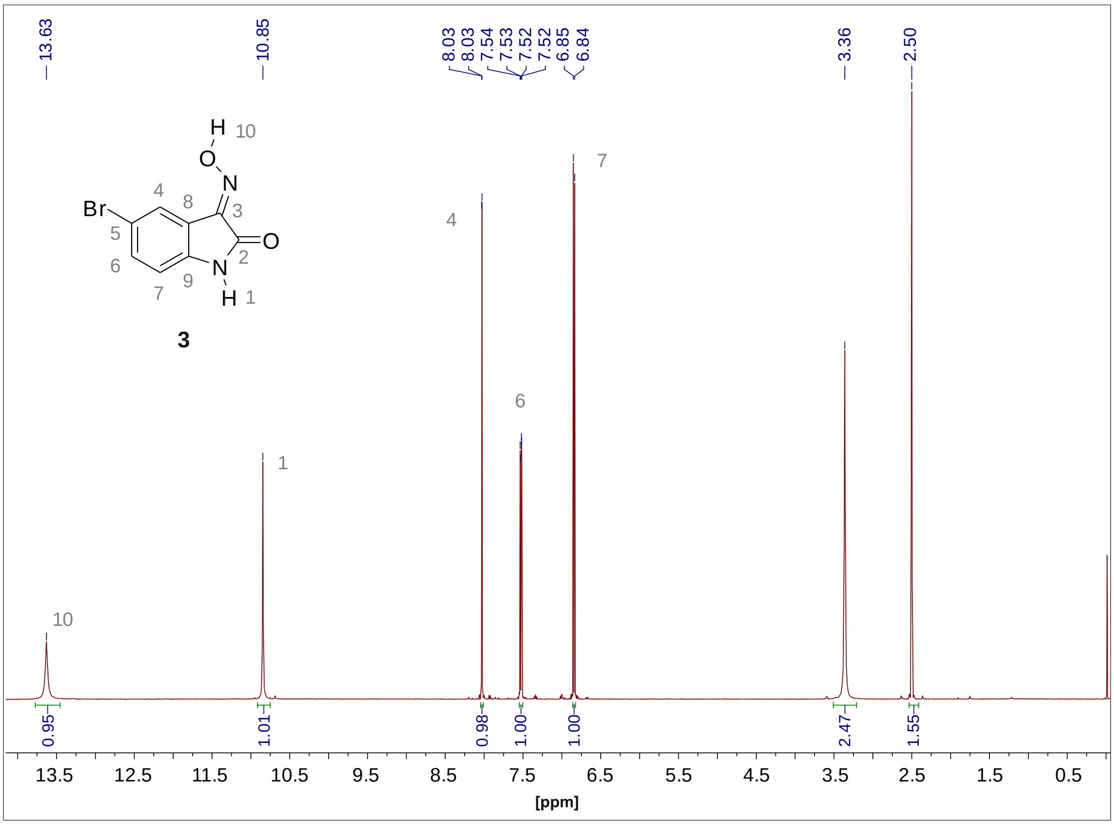

Figure S27. ^{1}H-NMR (500 MHz) spectrum of compound **3** in DMSO-d_6.

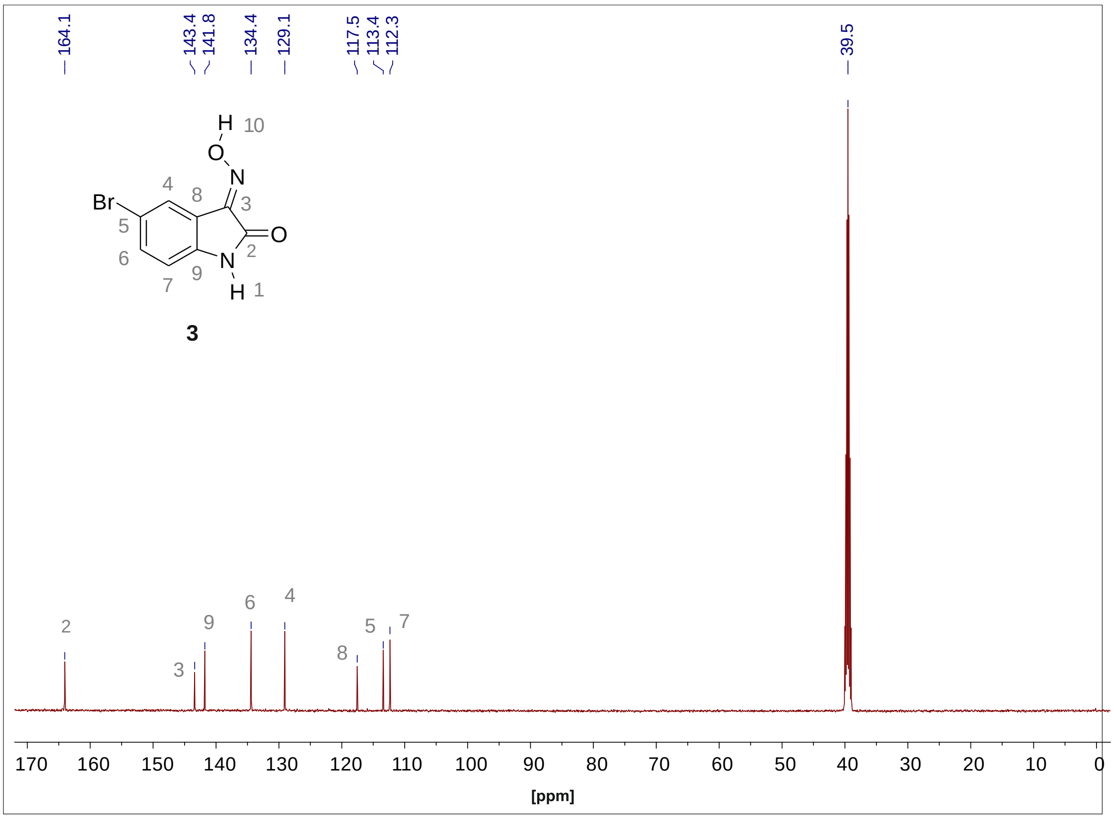

Figure S28. ^{13}C-NMR spectrum (125 MHz) of compound **3** in DMSO-d_6.

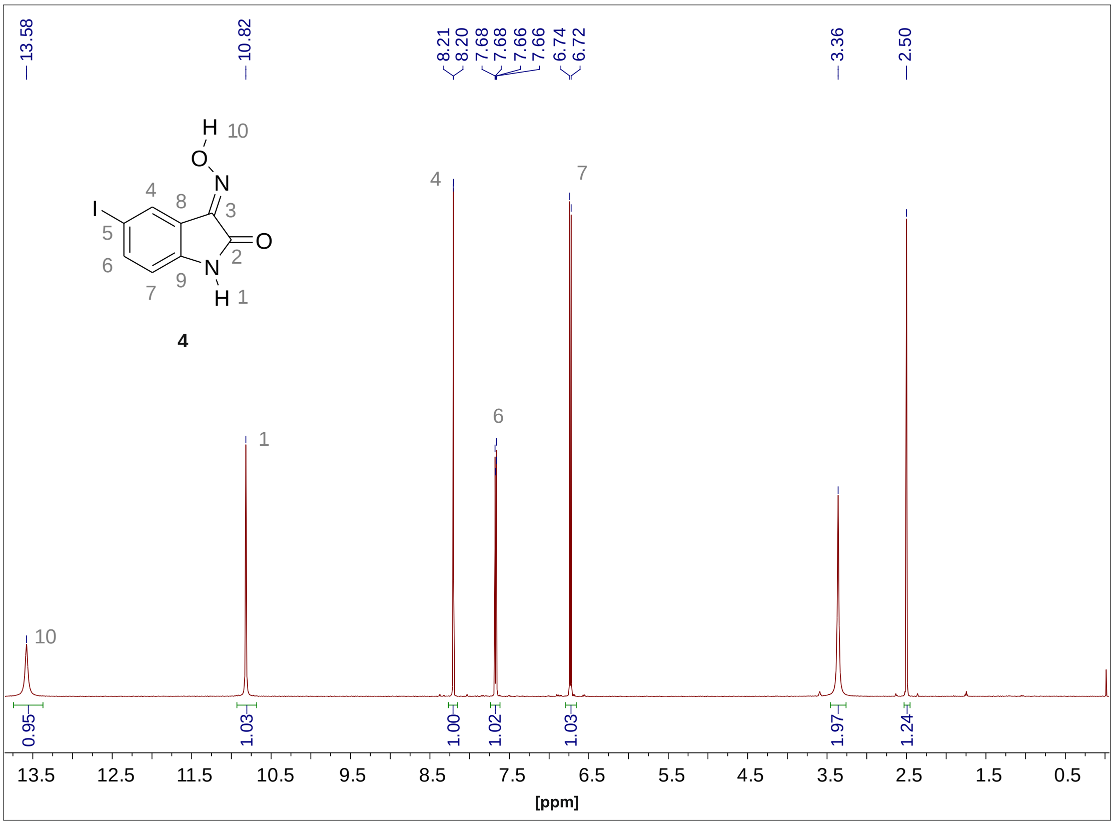

Figure S29. ^{1}H-NMR spectrum (500 MHz) of compound **4** in DMSO-d_6.

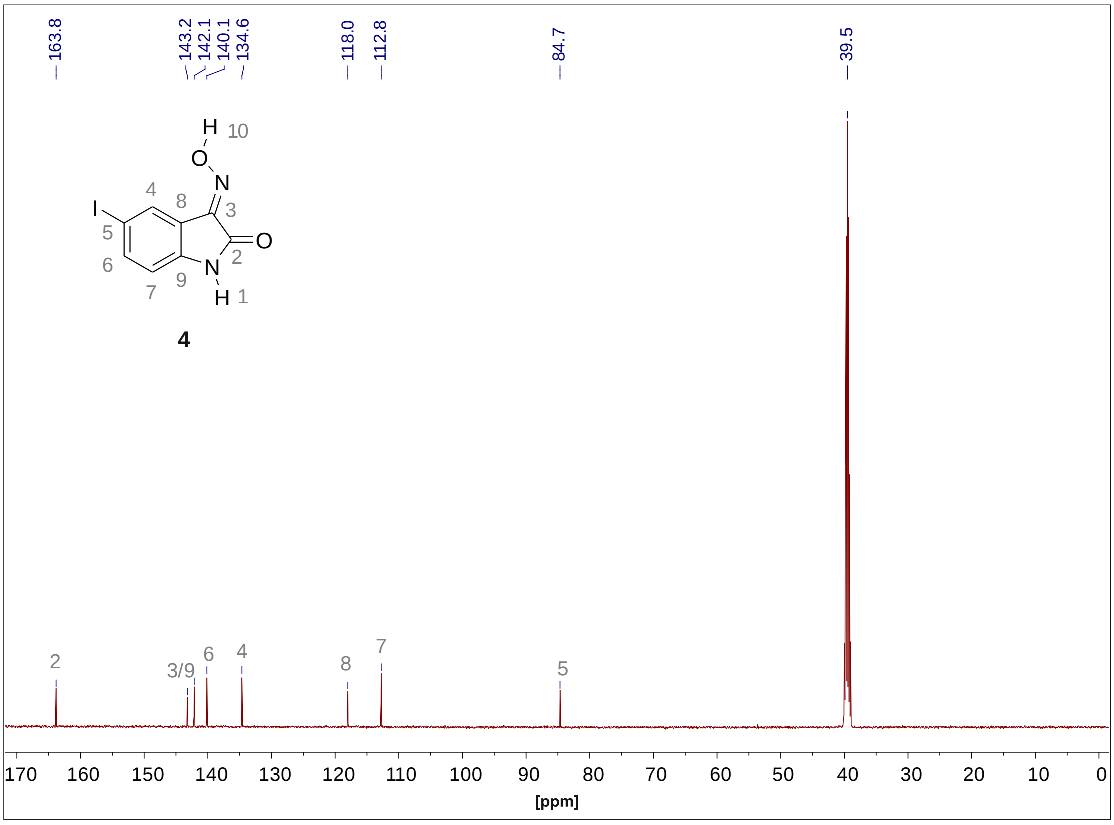

Figure S30. ^{13}C-NMR spectrum (125 MHz) of compound **4** in DMSO-d_6.

Synthetic procedures for 1-3 (further details)

*Synthesis of 1H-isatin-3-oxime (**1**):* Isatin (0.77 g, 5.23 mmol) and potassium carbonate (0.72 g, 5.21 mmol) were dissolved in a boiling mixture of ethanol (50 mL) and water (15 mL) and a solution of hydroxylamine hydrochloride (0.55 g, 7.85 mmol) in water (15 mL) was added. The mixture was refluxed for 6 h and stirred at room temperature for 14 h, during which time another portion of hydroxylamin hydrochloride (0.15 g, 2.09 mmol) was added. Afterward the solution's volume was reduced to about 10 mL by evaporation of the solvents and the precipitating solid was filtered off, washed with water and chloroform, recrystallized from water/ethanol (4:1 *v*/*v*) and dried *in vacuo* to obtain **1** as a bright yellow solid. Yield: 0.62 g (3.82 mmol, 74 %).

*Synthesis of 5-chloroisatin-3-oxime (**2**):* 5-Chloroisatin (0.70 g, 3.86 mmol) was dissolved in a boiling mixture of ethanol (40 mL) and tetrahydrofuran (5 mL). Afterwards, a solution of potassium carbonate (0.53 g, 3.86 mmol) and hydroxylamine hydrochloride (0.40 g, 5.78 mmol) in water (15 mL) was added. After refluxing for 5 h and diluting with water (20 mL), the precipitating solid was filtered off, washed with water, recrystallized from ethanol and dried *in vacuo* to obtain **2** as a yellow solid. Yield: 0.52 g (2.64 mmol, 69 %).

*Synthesis of 5-bromoisatin-3-oxime (**3**):* 5-Bromoisatin (0.50 g, 2.22 mmol) was dissolved in a mixture of ethanol (40 mL) and tetrahydrofuran (20 mL). Then a solution of potassium carbonate (0.26 g, 1.88 mmol) and hydroxylamine hydrochloride (0.23 g, 3.31 mmol) in water (20 mL) was added and the mixture stirred at room temperature for 1 d, during which time another portion of hydroxylamine hydrochloride (0.05 g, 0.72 mmol) was added. Thereafter the solution's volume was reduced to 20 mL via evaporation of the solvents and the precipitating solid filtered off. A second batch of solid was obtained by diluting the mother liquor with water, combined with the first batch, washed with water, recrystallized from water/tetrahydrofuran (1:1 *v*/*v*) and dried *in vacuo* to yield **3** as a yellow solid. Yield: 0.30 g (1.25 mmol, 56 %).

References

1. Sheldrick, G. M. Crystal structure refinement with SHELXL, *Acta Crystallogr. C* **2015**, *71*, 3–8, doi:10.1107/S2053229614024218.

2. *X-AREA*, version 1.75; Software package for data collection and data evaluation; Stoe & Cie: Darmstadt, **2015**.

3. *LANA*, version 1.63.1; Laue Analyzer; Stoe & Cie: Darmstadt, **2015**.

4. Spek, A.L. checkCIF validation ALERTS: what they mean and how to respond, *Acta Crystallogr. E* **2020**, *76*, 1–11, doi:10.1107/S2056989019016244.

5. Golushko, A.A.; Sandzhieva, M.A.; Ivanov, A.Y.; Boyarskaya, I.A.; Khoroshilova, O.V.; Barkov, A.Y.; Vasilyev, A.V. Reactions of 3,3,3-Trihalogeno-1-Nitropropenes with Arenes in the Superacid CF_3SO_3H: Synthesis of (*Z*)-3,3,3-Trihalogeno-1,2-diarylpropan-1-one Oximes and Study on the Reaction Mechanism. *J. Org. Chem.* **2018**, *83*, 10142–10157, doi:10.1021/acs.joc.8b01406.

6. Campbell, A.; Tasker, P.; Parsons, S. CCDC 1413226: Experimental Crystal Structure Determination, **2015**, doi:10.5517/cc1jfkyg.
